# Single‐Cell Mapping Identifies Aging‐Associated Neutrophil Remodeling Across Multiple Organs

**DOI:** 10.1111/acel.70735

**Published:** 2026-09-29

**Authors:** Yuman Wang, Teding Chang, Yangguang Fu, Guangyu Liang, Huicheng Liu, Le Zhang, Jinhua Yan, Man Liu, Anding Liu, Cuntai Zhang

**Affiliations:** ^1^ Department of Geriatrics, Tongji Hospital, Tongji Medical College Huazhong University of Science and Technology Wuhan China; ^2^ Key Laboratory of Vascular Aging, Ministry of Education, Tongji Hospital, Tongji Medical College Huazhong University of Science and Technology Wuhan China; ^3^ Department of Traumatic Surgery, Tongji Trauma Center, Tongji Hospital, Tongji Medical College Huazhong University of Science and Technology Wuhan China; ^4^ Department of Surgery University of Pittsburgh Pittsburgh Pennsylvania USA; ^5^ Experimental Medicine Center Tongji Hospital, Tongji Medical College, Huazhong University of Science and Technology Wuhan China; ^6^ Department of Otolaryngology‐Head and Neck Surgery, Tongji Hospital, Tongji Medical College Huazhong University of Science and Technology Wuhan China

**Keywords:** aging, bone marrow remodeling, inflammation, multi‐organ atlas, neutrophils

## Abstract

Aging is accompanied by progressive immune remodeling and functional decline across multiple organs. However, the role of neutrophils and their heterogeneous states in these processes remains incompletely understood. Here we integrated single‐cell RNA sequencing datasets from multiple organs of young and aged mice to characterize aging‐associated neutrophil remodeling. We identified organ‐dependent changes in neutrophil abundance and state composition, together with neutrophil subsets enriched for inflammatory, oxidative stress, complement, and IL‐6‐related transcriptional programs. In vitro, exposure to conditioned media from neutrophils isolated from aged mice or IL‐6‐stimulated neutrophil‐like cells increased inflammatory and senescence‐associated responses in vascular smooth muscle and hepatocyte‐derived cells. Bone marrow single‐cell analysis further revealed aging‐associated shifts in progenitor composition and transcriptional states, including an old‐enriched HPC11 population characterized by enhanced myeloid priming. Silico perturbation analysis nominated FGF didate regulators of HPC subtype‐specific transcriptional programs. Together, our study provides a multi‐organ single‐cell resource for defining neutrophil remodeling during aging and highlights candidate neutrophil states and potential signaling axes associated with age‐related inflammatory tissue environments.

## Graphical abstract

Single‐cell transcriptomic profiling across multiple organs identifies aging reshapes neutrophil differentiation from the bone marrow to peripheral tissues. Computation analysis identifies an age‐enriched Neu06‐IL6 neutrophil state and predicts IL‐6 as one of a candidate regulator for neutrophil remodeling. Functional validation supports a potential role for this pathway in tissue inflammatory and senescence‐associated changes.

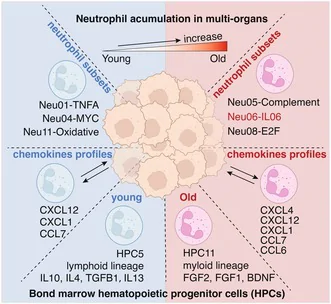

## 1 Introduction

In 2025, Kroemer and López‐Otín proposed the concept of precision geromedicine, which integrates the established hallmarks of aging and emphasizes the importance of identifying mechanisms that drive age‐associated functional decline (Kroemer et al. 2025). The immune‐cell states that are most consistently remodeled across aged tissues. However, their potential contribution to systemic inflammatory environments during multi‐organ aging remains incompletely defined.

The immune system undergoes dramatic changes with aging, including altered immune‐cell composition, increased cytokine secretion, and impaired recognition and clearance of senescent cells (Liu, Liang, et al. 2023). The propagation of senescence‐ associated signals within bone marrow has been reported in several cell types, including skeletal stem cells, bone marrow adipocytes, and bone marrow monocytes/macrophages (BMMs) (Ambrosi et al. 2021; Hou et al. 2024; Liu, Gu, et al. 2023). Neutrophils, one of the major innate immune cell types derived from bone marrow, eliminate microorganisms through phagocytosis, degranulation, and the formation of neutrophil extracellular traps (NETs) (Papayannopoulos 2018; Wigerblad and Kaplan 2023; Zhang, Xia, et al. 2024). Recent studies have shown that neutrophils contribute to pathology through disease‐context–dependent mechanisms, including excessive neutrophil extracellular trap formation and altered granulopoiesis, which can generate atypical neutrophil populations with altered inflammatory functions. These processes have been implicated, to varying extents, in stroke, Alzheimer's disease, atherosclerosis, and periodontitis (Gullotta et al. 2023; Van Avondt et al. 2023; Wang et al. 2017; Wang et al. 2025). Although changes in the proportion of peripheral‐blood neutrophils during aging remain debated, multiple studies indicate that neutrophil functional capacity declines or becomes dysregulated in older individuals (Valiathan et al. 2016; Wenisch et al. 2000). Neutrophils can also induce paracrine telomere dysfunction and cellular senescence in a reactive oxygen species (ROS)‐dependent manner (Lagnado et al. 2021). In addition, age‐related alterations in the microvasculature enhance leukocyte adhesion to the endothelium and disrupt normal diapedesis (Reglero‐Real et al. 2025). During inflammation, retrograde endothelial migration (rTEM) of neutrophils in aged mice promotes distal organ injury, such as in the lungs (Barkaway et al. 2021), suggesting that aged vascular environments may facilitate neutrophil redistribution across organs. These observations indicate substantial age‐associated differences in neutrophils from young and aged organisms. Therefore, systematic characterization of neutrophil alterations under steady‐state aging conditions is important for understanding how innate immune remodeling may contribute to multi‐organ aging.

High‐throughput analytical technologies have become powerful tools for aging research, enabling interrogation of biological processes across temporal and spatial dimensions (Adrover et al. 2019; Gullotta et al. 2023). Although many single‐cell transcriptomic datasets from aged mouse organs are now available, they remain underexplored with respect to neutrophil biology in multi‐organ aging (Adrover et al. 2019). In this study, we integrated multi‐organ RNA‐sequencing datasets from aged and young mice to systematically identify the aging‐related neutrophil subtype atlas and find the most aging‐related neutrophil subset, which may be a more potential neutrophil program that can indicate and contribute to multi‐organ aging.

## 2 Results

### 2.1 R1. Neutrophil Accumulation Is Increased Across Multiple Organs in Aged Mice

To systematically analyze the effects of immune cell populations in aged mice, we collected single‐cell datasets from multiple organs, including lungs, hearts, brains, vascular, peripheral blood, intestines, kidneys, and livers for a total of 8 tissues (Figure 1A). Each dataset underwent separate quality control, clustering, and cell‐type annotation (Figure S1A,B). Batch effects arising from disparate sequencing platforms were mitigated with reciprocal principal component analysis (RPCA) integration, yielding a unified atlas of 256,949 cells (Figure 1B). The atlas comprises 66 samples from 8 tissues, including lung (56,782 cells), heart (37,805 cells), brain (35,699 cells), vascular (28,014 cells), peripheral blood (14,588 cells), intestine (25,788 cells), kidney (43,636 cells), and liver (56,782 cells) (Figure 1C and Figure S1C).

**FIGURE 1:**
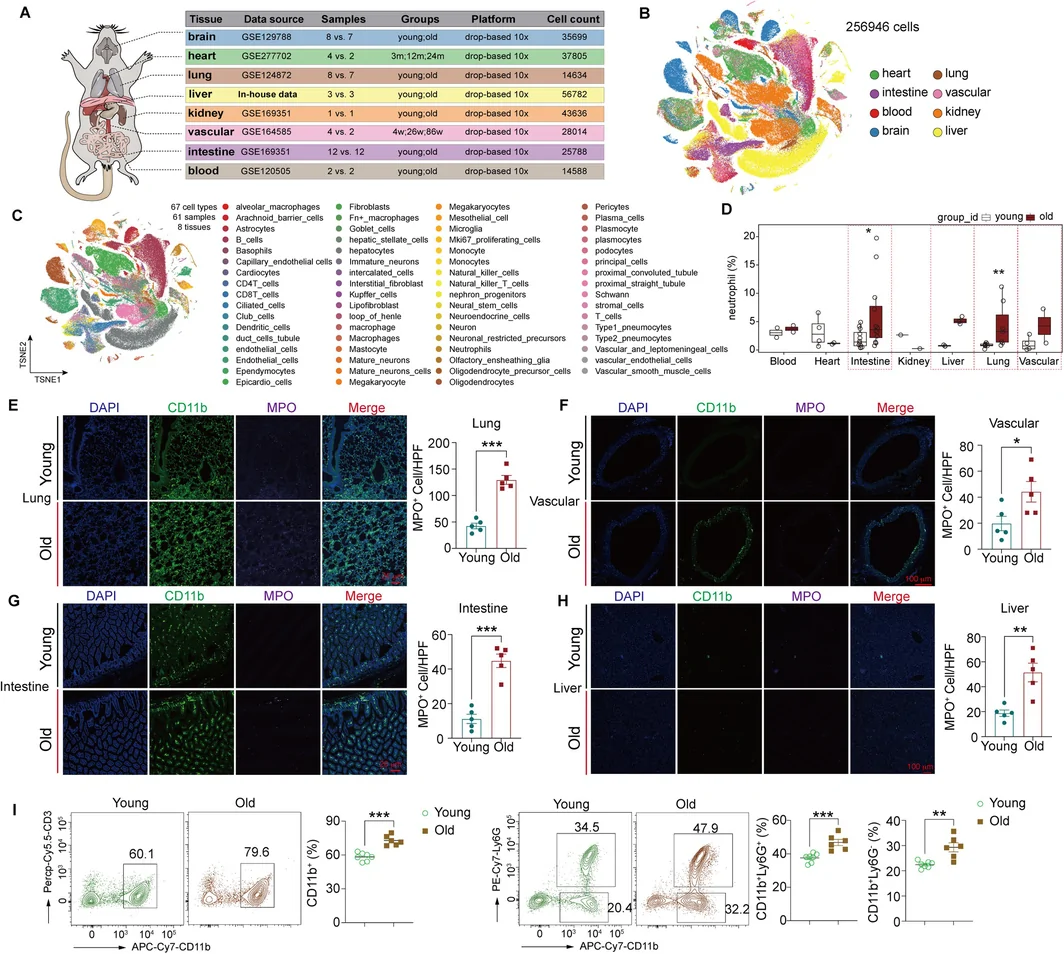
Neutrophil accumulation is increased across multiple organs in aged mice. (A) Schematic overview of scRNA‐seq sampling of multiple organs from young and aged mice. (B, C) t‐SNE plots of integrated scRNA‐seq data from multiple organs, colored by tissue origins (B) or annotated cell types (C). (D) Box plots showing the sample‐level proportions of neutrophils among all captured cells in each tissue from young and aged mice. Each dot represents one biological sample. Boxes indicate the interquartile range, center lines indicate the median, and whiskers extend to 1.5 × the interquartile range. (E–H) Immunofluorescence of indicated tissues in young and old mice. Green: CD11b, Purple: MPO, Blue: DAPI. Scale bars are shown in the picture. (I) Flow cytometric analysis of CD11b^+^CD3^−^ cells (left) and CD11b^+^Ly6G^+^ cells (right) in bone marrow from young and aged mice (*n* = 6 mice per group). Data are shown as the mean ± s.e.m., *p < 0.05; **p < 0.01; ***p < 0.001. Wilcox rank sum test was used in (D), and *t*‐test was used in (E–I).

Across several tissues, including peripheral blood, heart, intestine, liver, lung, and vascular tissue, aging was accompanied by altered immune‐cell composition (Figure S1C). Neutrophil abundance was increased in multiple tissues of aged mice compared with young counterparts. Notable elevations were observed in the liver (young vs. old, 0.75% vs. 5.26%), lung (young vs. old, 0.88% vs. 4.14%), vascular (young vs. old, 1.05% vs. 3.68%), and intestine (young vs. old, 1.22% vs. 3.57%) (Figure 1D). In contrast, no significant increase in neutrophil accumulation was detected in the brain, likely reflecting the restrictive nature of the blood–brain barrier, which limits the entry of circulating immune cells such as neutrophils. Sample‐level Bayesian compositional analysis using scCODA further supported an overall age‐associated increase in neutrophil abundance, with credible increase detected in the liver (inclusion probability = 0.968; log2 fold change = 2.61), whereas lung and intestine showed nonsignificant trends and no independent credible shifts were detected in the remaining aggregated non‐neutrophil populations.

To validate these findings, we performed fluorescence staining and revealed a marked increase in CD11b^+^MPO^+^ cells across multiple organs in aged mice, including lung, intestine, vascular, and liver (Figure 1E–H). Flow cytometry analysis of bone marrow further showed a higher proportion of neutrophils in aged mice than in young mice (Figure 1I). Hematoxylin and eosin (HE) staining of tissues from young and aged mice illustrated age‐associated morphological alterations (Figure S2). These results indicate that neutrophil accumulation is increased across multiple organs during aging.

### 2.2 R2. Neutrophils From Aged Mice Display Enhanced Inflammatory and Effector‐Associated Programs

To investigate the functional characteristics of neutrophils from aged mice, we isolated and constructed a single‐cell neutrophil atlas from the multi‐organ datasets above, comprising 5210 cells, including 2400 neutrophils from young mice and 2810 from aged mice (Figure 2A). Pseudobulk differential gene expression analysis identified 26 genes upregulated in neutrophils from aged mice and 985 genes enriched in neutrophils from young mice (Figure 2B), indicating age‐associated remodeling of neutrophil transcriptional states. Gene set enrichment analysis using the ranked pseudobulk differential‐expression results showed that glycolysis and reactive oxygen species pathways were enriched in age‐associated neutrophils, whereas TNF‐α signaling and IFN‐λ signaling pathways were enriched in young neutrophils (Figure 2C,D).

**FIGURE 2:**
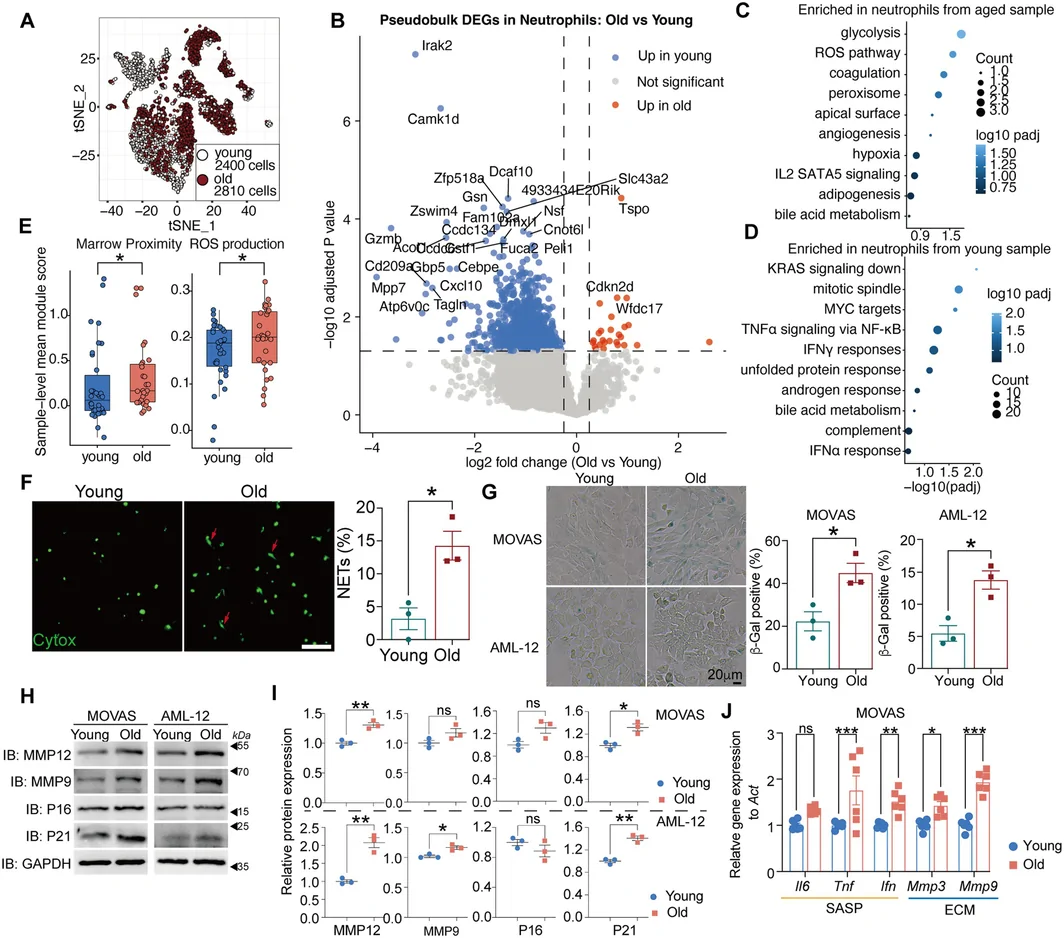
Neutrophils from aged mice display enhanced inflammatory and effector‐associated programs. (A) t‐SNE plot of the integrated neutrophil atlas from multiple organs, including blood, intestine, liver, lung, vascular tissue, heart, kidney, and brain, colored by age group. The number of neutrophils contributed by each group is shown in the accompanying bar plot. (B) Volcano plot showing differential gene expression in neutrophils from aged versus young mice. Genes with adjusted *p* value < 0.05 and log2 Fold Change > 0.5 were designated as differentially expressed genes. (C, D) Hallmark functional enrichment analysis of gene sets enriched in neutrophils from aged (C) or young (D) samples. Dot size represents gene count and color indicates adjusted significance. (E) Sample‐level comparison of marrow proximity and reactive oxygen species (ROS) module scores in neutrophils from young and aged mice. Each dot represents one biological sample. (F) Representative Cytox Green staining images showing NET formation in neutrophils isolated from young and aged mice. Quantification of NET‐positive cells is shown on the right, each data point represents one biological replicate. Scale bar, 50 μm. (G) Representative senescence‐associated β‐galactosidase (β‐Gal) staining images of MOVAS and AML‐12 cells following treatment with conditional medium derived from young or aged neutrophils. Quantification of β‐Gal‐positive cells is shown on the right, each data point represents one biological replicate. Scale bar, 20 μm. (H, I) Immunoblot (H) and its quantification analysis (I) of MMP12, MMP9, P16, and P21 expression in MOVAS and AML‐12 cells treated with conditioned medium from young or aged neutrophils. GAPDH served as a loading control. Relative protein intensities normalized to young group are indicated below each blot. Each plot represents an independent biological replicate. (J) Relative RNA expression of senescence‐associated secretory phenotype (SASP) genes (*Il6*, *Tnf*, and *Ifn*) and extracellular matrix remodeling genes (*Mmp3* and *Mmp9*) in MOVAS cells following treatment with conditioned medium from young or aged neutrophils. Data are presented as mean ± s.e.m. Statistical significance was determined using two‐sided unpaired *t*‐test (E–G, I) and two‐way ANOVA followed by Tukey's multiple‐comparisons test (J). *p < 0.05; **p < 0.01; ***p < 0.001.

To delineate functional reprogramming in age‐associated neutrophils (AANs), we compared key effector pathways with those of neutrophils from young mice. Neutrophils from aged tissues exhibited relatively high gene‐signature scores for ROS production and marrow‐proximity programs at the sample level (Figure 2E). Similar trends were observed across neutrophils isolated from several organs (Figure S3A–D), suggesting that inflammatory effector programs are recurrent features of aged neutrophils. These data support the interpretation that aging is associated with a hyper‐responsive neutrophil state characterized by increased oxidative transcriptional programs.

To further assess the effects of the AANs, we isolated neutrophils from bone marrow of either young or aged mice. After 4 h of in vitro culture, neutrophils from aged mice showed increased cell death and a higher propensity for NETs formation compared with those from young mice (Figure 2F). To further examine whether neutrophils from aged mice can promote senescence‐like and inflammatory responses in tissue cells, we then collected the culture medium of neutrophils from the indicated group and applied it to mouse vascular aortic smooth muscle cells (MOVAS) and alpha‐mouse liver 12 cells (AML‐12) for another 48 h. In both MOVAS and AML‐12 cells, conditioned medium from aged neutrophils increased the proportion of beta‐galactosidase (β‐Gal) positive cells (Figure 2G). Several, but not all, markers of extracellular matrix remodeling (MMP12 and MMP9) and cellular senescence‐associated signaling (P16 and P21) were modestly up‐regulated following exposure to aged neutrophil‐conditioned medium (Figure 2H,I). Quantitative PCR analysis further showed increased expression of selected SASP‐related genes in MOVAS after exposure to conditioned medium from aged neutrophils (Figure 2J).

Together, these findings indicate that neutrophils from aged mice exhibit enhanced NETs formation and ROS‐related generation programs. The secreted factors from aged neutrophils potentially further induce early stress‐associated responses in MOVAS and AML‐12 cells.

### 2.3 R3. Aging Is Associated With Expansion of Atypical Neutrophil States Across Tissues

To further delineate neutrophil heterogeneity and identify the aging‐associated neutrophils (AANs), we re‐clustered all neutrophils using the standard Seurat scRNA‐seq workflow. The clustering resolution was selected after evaluating cluster stability, marker specificity, and biological interpretability, leading to 16 transcriptional neutrophil subtypes (Neu01‐Neu16) (Figure 3A). To assess whether the identified clusters were influenced by integration‐related bias, we independently re‐integrated the dataset using Harmony. This analysis reproduced the major neutrophil subtype structure, supporting the robustness of the clustering results (Figure S4A–C). We used the clustree method to validate the stability of these 16 neutrophil subtypes (Figure S4D–G). Each cluster exhibited distinct marker‐gene expression and pathway activity profiles, which informed their Hallmark functional annotation and revised nomenclature (Figure 3B and Table S1). These neutrophil subtypes were distributed across tissues and broadly classified by tissue‐enrichment patterns as widespread, shared, or organ‐enriched states.

**FIGURE 3:**
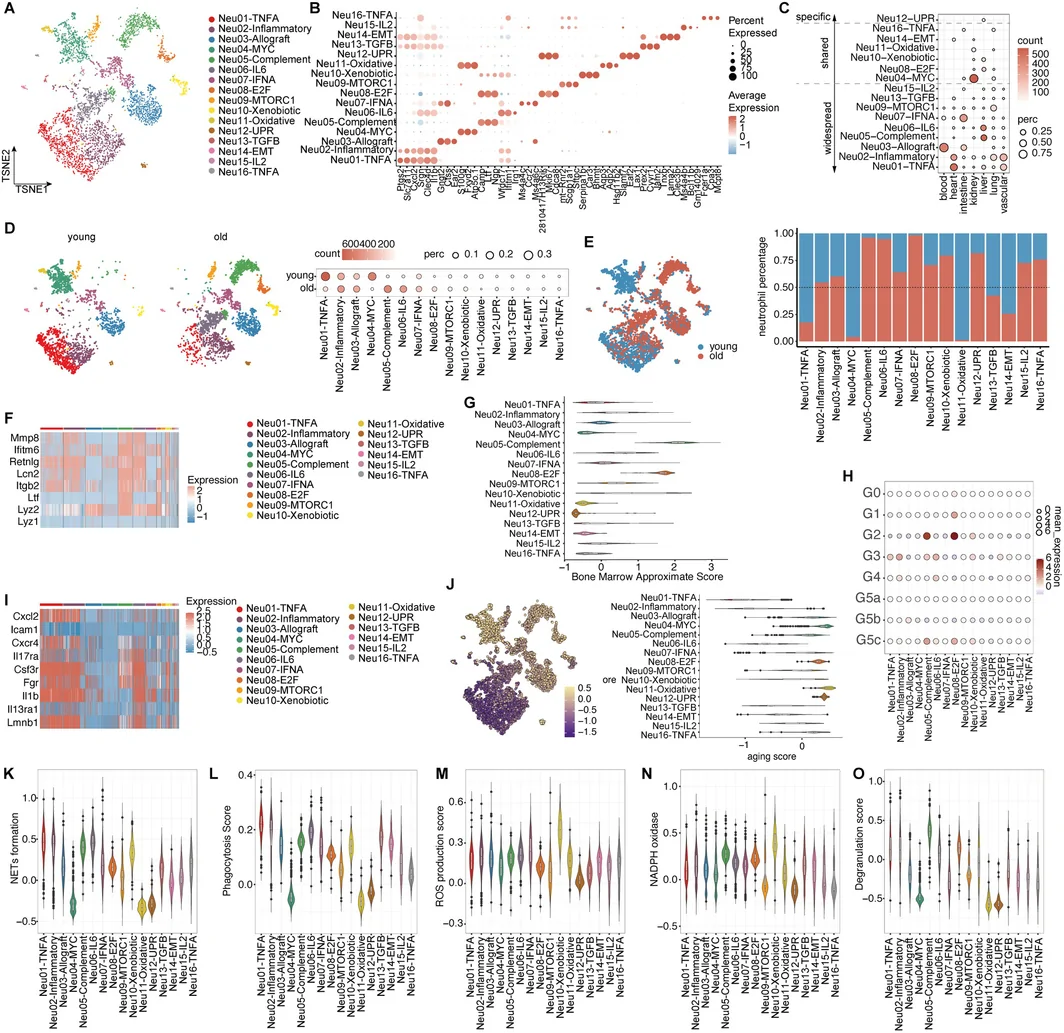
Aging is associated with expansion of atypical neutrophil states across tissues. (A) t‐SNE plot of integrated neutrophil scRNA‐seq data, colored by neutrophil subtypes. (B) Dot plot showing the top three marker genes for each neutrophil subtype. Dot size indicates the percentage of expressing cells; color reflects average expression levels. (C) Distribution of neutrophil subtypes across different tissues. Dot size represents the percentage of expressing cells; color denotes the number of expressing cells. (D) t‐SNE plot showing age‐specific distribution of neutrophils in young versus aged mice (left panel). Dot plot illustrating enrichment of neutrophil subtypes by age group; dot size indicates cell percentage, and color indicates cell count (right panel). (E) t‐SNE plot colored by indicated group (left panel). Bar plot showing the proportion of young and aged neutrophils within each subtype (right panel). (F) Heatmap showing row‐scaled expression levels of genes used to calculate the bone marrow (BM) proximity score. (G) Violin plot depicting BM proximity scores across neutrophil subtypes. Embedded boxplots indicate interquartile ranges (IQRs). Center line marks the median. (H) Dot plot showing the similarity score of each neutrophil subtype aligned to a reference scRNA‐seq dataset of neutrophil developmental stages. Dot size and color represent mean similarity scores. (I) Heatmap displaying row‐scaled expression of genes used to compute the neutrophil aging score. (J) t‐SNE plot colored by the aging score of neutrophils (left panel). Violin plot showing aging scores across neutrophil subtypes (right panel). Embedded boxplots represent IQRs; the center line indicates the median. (K–O) Violin plots showing functional module scores across neutrophil subtypes including NETs formation (K), phagocytosis (L), ROS production (M), NADPH oxidase activity (N), and degranulation (O). Embedded boxplots display IQRs and medians.

Widespread neutrophils, enriched in more than three tissues, represented the majority and included Neu01, Neu02, Neu03, Neu05, Neu06, Neu07, Neu09, Neu13, and Neu15. These clusters were characterized by high expression of pro‐inflammatory mediators, including *Tnfa*, *Il6*, *Il2*, *Ifna*, and complement components, suggesting a predominant pro‐inflammatory phenotype. Notably, subsets with immunosuppressive features also emerged within this group, exhibiting elevated expression of *Tgfb* and other inhibitory cytokines across multiple organs (Figure 3C). Shared neutrophils, enriched in two or three organs, were composed of Neu04, Neu08, Neu10, Neu11, Neu14, and Neu16. These subtypes were predominantly immunoregulatory, expressing genes associated with cell proliferation (*Myc* and *E2f*) and epithelial‐to‐mesenchymal transition (EMT), and were frequently localized to barrier tissues such as the intestine, kidney, and lung, which are commonly exposed to environmental stimuli (Figure 3C). Organ‐specific neutrophils were represented by a single cluster, Neu12, which was exclusively enriched in the liver. This cluster showed elevated expression of unfolded protein response (UPR) genes, indicating activation of endoplasmic reticulum stress pathways likely triggered by protein misfolding (Figure 3C).

To investigate age‐dependent changes in these neutrophil subsets, we performed cellular enrichment analysis and identified that the neutrophils from old mice were predominantly enriched in Neu05‐complement, Neu06‐IL06, and Neu08‐E2F, and neutrophils from young mice included Neu01‐TNFA and Neu04‐Myc (Figure 3D,E). These observations suggest that aging is associated with a shift in neutrophil‐state composition toward complement‐associated, IL‐6‐related, and E2F target gene expression programs.

To refine the functional and maturation context of each neutrophil state, we calculated signature‐based scores related to bone marrow‐associated maturation (BM proximity) and cellular aging. The BM proximity score was adapted from a prior single‐cell study of neutrophil diversification and reflects transcriptional similarity to bone marrow‐associated immature neutrophil programs (Vafadarnejad et al. 2020). Neu05‐Complement and Neu08‐E2F showed relatively high BM proximity scores and expressed several genes associated with immature neutrophil states (Figure 3F,G), suggesting that these aged‐enriched neutrophils retained transcriptional similarity to early marrow‐associated neutrophil states. To place these clusters along the canonical maturation continuum, we integrated our scRNA‐seq data with a published neutrophil developmental trajectory spanning several stages: hematopoietic stem and progenitor cells (HSPCs), CMP/GMP (G0), proNeu (G1), preNeu (G2), immature neutrophil (G3), marrow‐maturing neutrophil (G4), and peripheral mature neutrophils (G5a, G5b, and G5c) (Xie et al. 2020). This mapping suggested that Neu05‐Complement and Neu08‐E2F are most like the preNeu stage (G2) (Figure 3H).

Beyond maturation, circulating neutrophils continue to acquire transcriptional features associated with the neutrophil “aging” clock genes (Adrover et al. 2019). Aging‐score analysis showed that Neu04‐MYC, Neu11‐Oxidative, and Neu12‐UPR harbor the highest relative enrichment of this neutrophil aging‐associated signature, whereas Neu01‐TNF and Neu02‐Inflammatory showed the lowest enrichment (Figure 3I,J). Together, these results suggest that Neu05 and Neu08 show transcriptional features consistent with early marrow‐emigrant neutrophils, whereas Neu04, Neu11, and Neu12 display relatively higher enrichment of the previously reported neutrophil aging‐associated signature. In contrast, Neu01‐TNF and Neu02‐Inflammatory showed lower enrichment of this signature, suggesting heterogeneity in aging‐related transcriptional programs across neutrophil subsets.

To characterize putative effector transcriptional programs across neutrophil subsets, we calculated module scores for five signatures associated with NET formation, phagocytosis, ROS generation, NADPH oxidase‐related processes, and degranulation. Neu05‐Complement and Neu06‐IL‐6 exhibited relatively high scores across several of these signatures, whereas Neu01‐TNFα and Neu02‐Inflammatory showed relatively higher NET formation and phagocytosis scores but lower ROS generation and degranulation scores (Figure 3K–O). These patterns highlight functional transcriptional heterogeneity among neutrophil subsets and suggest that subsets more frequently observed in aged samples are characterized by distinct inflammatory and oxidative gene‐expression programs.

To evaluate whether the observed neutrophil remodeling is conserved across tissues and not solely driven by the structure of the pooled dataset, we re‐clustered neutrophils within each organ and re‐evaluated their functional states (Figures S5–S11). In vascular samples, five neutrophil subsets were identified (Figure S5A,B), including an age‐enriched Neu05 cluster that showed markedly high bone marrow‐associated maturation scores (Figure S5C–G). The Neu05 also exhibited aging signatures, elevated pro‐inflammatory cytokine levels, NET‐associated degranulation, and ROS‐related module scores (Figure S5H–N). SCENIC regulon analysis further indicated increased STAT3 activity in age‐enriched Neu05, suggesting this pathway underpins its age‐associated program (Figure S5O). Similar age‐associated enrichment of inflammatory and oxidative neutrophil programs was observed in several other organs (Figures S6–S11). Together, these tissue‐level analyses support the presence of recurrent aging‐associated neutrophil remodeling while reducing the likelihood that the major observations are solely explained by dataset composition.

To further assess cross‐tissue functional convergence, we compared neutrophil programs across organs (Figure S12). Neutrophils showed substantial tissue‐specific functional heterogeneity (Figure S12A), while age‐associated changes were most prominent in kidney and liver, particularly in inflammatory, degranulation, NETosis, and oxidative programs (Figure S12B). Cross‐tissue meta‐program analysis further identified recurrent maturation/aging, phagocytic, degranulation, and ROS‐related programs across independently defined neutrophil states, while preserving clear tissue‐specific features (Figure S12C). These findings support a shared core of aging‐associated neutrophil remodeling superimposed on tissue‐specific transcriptional programs.

To further validate these phenotypic features and assess their conservation, we analyzed neutrophils from the Tabula Sapiens multi‐organ single‐cell atlas with a human dataset (Tabula Sapiens et al. 2022) (GSE201333). Following ortholog mapping and integration of human neutrophil transcriptomes, we identified 14 transcriptionally distinct neutrophil states (Figure S13A–C). Several human neutrophil states exhibited transcriptional signatures similar to the age‐enriched mouse clusters, including complement‐associated, IL‐6‐associated, oxidative stress‐associated, IFNα‐associated, and mTOR‐related programs. To account for the non‐independence of cells derived from the same individual, neutrophil‐state proportions were quantified at the donor level, with each donor treated as the biological replicate. Donor‐level analysis showed higher proportions of several inflammatory and stress‐responsive neutrophil states in older compared with younger individuals (Figure S13D). Although direct one‐to‐one correspondence between mouse and human clusters cannot be assumed, the conservation of major transcriptional programs across species supports the presence of recurrent AAN states in mammals.

Collectively, these analyses demonstrate that aging is associated with substantial remodeling of neutrophil‐state composition across multiple tissues. Age‐enriched neutrophil states were characterized by complement activation, IL‐6 signaling, oxidative stress, and proliferative‐associated transcriptional programs, whereas young‐enriched states exhibited distinct inflammatory and homeostatic features. The recurrence of these transcriptional programs across tissues and their partial conservation in human datasets support the existence of broadly conserved aging‐associated neutrophil states.

### 2.4 R4. Cross‐Tissue Co‐Occurrence Analysis Reveals an Age‐Associated Neutrophil Module Associated With Chemokine Remodeling

To explore associations between aged tissue and neutrophil accumulation, we performed cross‐sample cell‐type co‐occurrence analysis across all tissues. This analysis included immune and non‐immune tissue‐resident cell types. We identified seven co‐varying multi‐cellular modules (CMs), each representing coordinated variation in cell‐type proportions across samples (Figure 4A). Among the seven identified CMs, CM3 was the most neutrophil‐enriched module and contained several aged‐enriched neutrophil states, including Neu05‐Complement and Neu06‐IL6 (Figure 4A). CM3 also contained endothelial cells and fibroblasts, suggesting that neutrophil abundance covaries with stromal and vascular cell compartments in some aged tissues. In contrast, CM2 was dominated by brain‐resident cell types, and CM6 consisted predominantly of kidney‐associated epithelial and immune cells, illustrating that some modules are strongly influenced by tissue composition. CM3 represents a cross‐sample co‐occurrence pattern that may capture tissue contexts in which neutrophil accumulation and stromal remodeling are jointly enriched.

**FIGURE 4:**
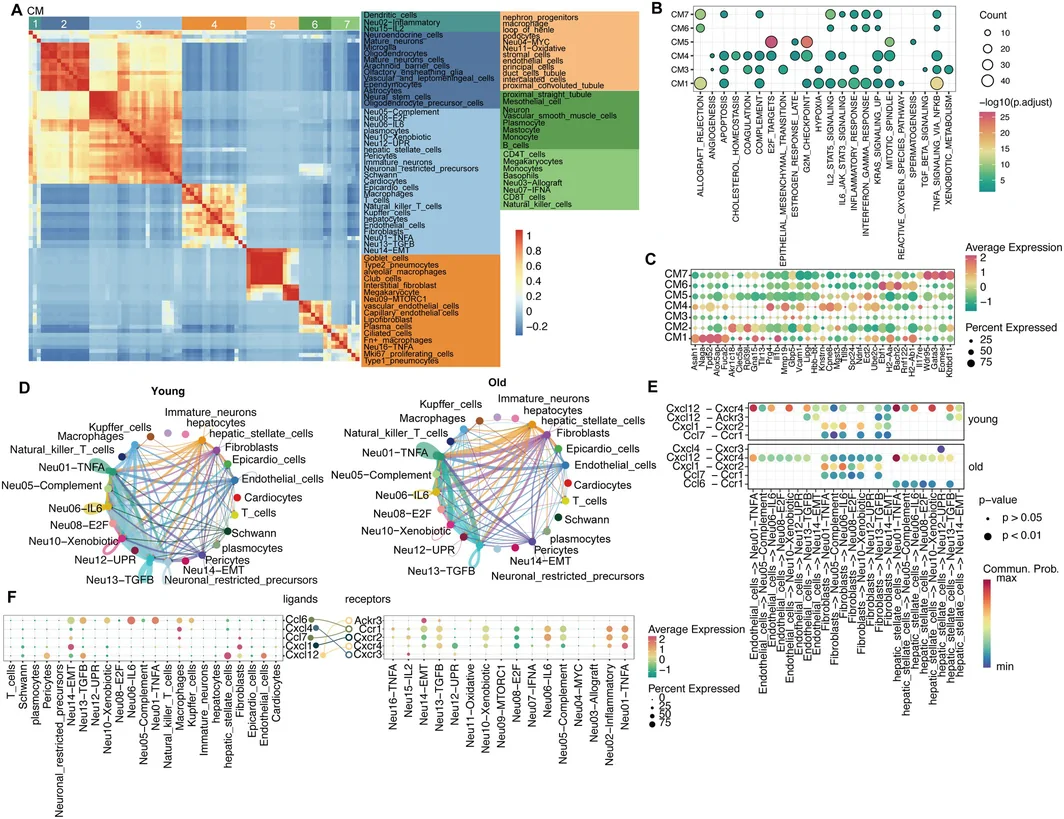
Cross‐tissue co‐occurrence analysis reveals an age‐associated neutrophil module associated with chemokine remodeling. (A) Heatmap displaying pairwise Pearson correlation coefficients of cell‐type proportions across tissue samples from different age groups. Cellular modules (CMs) are indicated by colored boxes above the heatmap. (B) Dot plot showing pathway enrichment analysis of different CMs. Dot size represents the number of enriched genes (hit count), and color indicates the statistical significance (−log_10_ adjusted *p* value). (C) Dot plot illustrating the marker genes of each CM. Dot size represents the percentage of neutrophils expressing each marker, and color reflects the average expression level. (D) Chord diagram visualizing the strength of cell–cell interactions mediated by CM3 across age groups. Line width represents interaction strength, and color denotes interacting cell types. (E) Dot plot comparing ligand–receptor interaction strengths between young and aged mice within CM3. Dot size indicates the *p* value, and color represents communication probability. (F) Dot plot showing expression levels of ligands (from neutrophils) and receptors (from other CM3‐associated cell types) involved in significant interactions. Dot size indicates the percentage of expressing cells; color reflects average gene expression.

To further assess the robustness of these modules and evaluate the potential influence of tissue composition, we performed a leave‐one‐tissue‐out (LOTO) analysis in which each tissue was sequentially excluded, and the co‐occurrence analysis was repeated. Variable module recurrence and tissue dependence, with some modules, including CM3, showing relatively robust recurrence across tissue‐removal conditions, whereas others are strongly influenced by individual tissues, particularly the brain‐, kidney‐, liver‐, lung‐, and heart‐associated modules (Figures S14 and S15A). Importantly, the major cellular features of CM3 were maintained after removal of individual tissues, suggesting that this module is not driven by any single tissue source but rather represents a recurrent cross‐tissue co‐occurrence pattern.

CM1 represented an immune‐enriched interaction module composed mainly of dendritic cells, Neu02‐Inflammatory, and Neu15‐IL2 neutrophils, suggesting potential coordinated roles in immune regulation. Modules CM4, CM5, and CM6 were classified as “oligo‐neutrophilic modules”, each containing only 1–2 neutrophil subtypes with limited functional engagement. Notably, CM3 housed the most diverse neutrophil repertoire, including neutrophils enriched in young mice (Neu01‐TNFA, Neu13‐TGFβ, and Neu14‐EMT) alongside multiple senescence‐associated subsets (Neu05‐Complement, Neu06‐IL6, Neu08‐E2F, Neu10‐Xenobiotic, and Neu12‐UPR). The co‐occurrence of these subtypes suggests that CM3 may represent a critical hub for neutrophil functional reprogramming in response to the aging microenvironment, potentially facilitating the acquisition of senescence‐related phenotypes. To investigate the biological features associated with each CM, we performed differential gene expression analysis for each CM and conducted pathway enrichment on the upregulated genes. These analyses suggested that aged tissues show altered neutrophil‐associated cellular composition and chemokine signaling (Figure 4B,C).

Neutrophil accumulation in aged tissues may depend on two coordinated processes: chemokine release by resident cells and neutrophil responsiveness to these chemokines (Capucetti et al. 2020). CellChat analysis suggested that chemokine signaling networks are upregulated in aged mice, with hepatic stellate cells and fibroblasts predicted to transmit stronger chemotactic signals to neutrophils than their counterparts in young mice (Figure 4D). Pathway‐level inspection showed that, in aged animals, endothelial cells, fibroblasts, and hepatocytes exhibited predicted increases in CXCL1‐, CCL7‐, CCL6‐, and CCL4‐associated signaling relative to CXCL12‐associated signaling (Figure 4E). Ligand‐receptor mapping further suggested that the young‐enriched Neu01‐TNFα subset preferentially engages the CXCL12‐CXCR4 axis, whereas the age‐expanded Neu06‐IL6 and Neu05‐Complement subsets are associated with the CXCL1‐CXCR2 pair (Figure 4F). Surface‐receptor expression within individual neutrophil subtypes was stable across ages (Figure S15B). Collectively, these data demonstrate the age‐associated chemokine landscapes.

Neutrophil chemotaxis exhibited tissue‐specific variability in aged mice. Chemotactic signaling toward neutrophils was globally increased across tissues (Figure S15C–I). Certain tissues, such as the heart (Figure S15D) and kidney (Figure S15F), showed reduced and weak chemotactic strength. This pattern reflects neutrophil infiltration levels observed in these tissues, with aged individuals exhibiting lower neutrophil accumulation compared to young mice. In contrast, vascular tissues demonstrated enhanced neutrophil‐attracting capacity, driven primarily by endothelial cells, fibroblasts, and other immune cells. Several vascular cell types in aged mice showed significantly elevated chemotactic activity toward neutrophils compared to other organs (Figure S16A). A similar trend was observed in the liver, where hepatic stellate cells, Kupffer cells, macrophages, and NKT cells contributed to the increased neutrophil recruitment observed in aged mice (Figure S16B). In the heart, neutrophil recruitment was mediated by endothelial cells, fibroblasts, macrophages, and pericytes; however, this chemotactic signaling was diminished in the aged heart relative to that in young hearts (Figure S16C). Conversely, lung tissue from aged mice exhibited heightened chemotaxis, particularly mediated by resident immune cells, including T and NK cells (Figure S16D). In peripheral blood and intestinal tissues, neutrophil recruitment relied heavily on signals from other immune cells. In these tissues, the chemotactic effect on neutrophils was significantly reduced in aged individuals (Figure S16E,F). Collectively, these data suggest that aging reshapes tissue chemokine landscapes and is associated with recurrent neutrophil‐stromal co‐occurrence patterns, providing a potential framework for understanding tissue‐specific neutrophil accumulation in aged organs.

### 2.5 R5. Age‐Enriched Neu06‐IL6 Neutrophils Are Associated With Inflammatory Tissue Remodeling

Given that neutrophil states are closely linked to their functional properties, we used Monocle 3 to reconstruct the inferred pseudotemporal ordering of neutrophil states and assigned a pseudotime score to each cell (Cao et al. 2019). Neu06‐IL6 cells were preferentially distributed within the distal, high‐pseudotime region of the inferred pseudotemporal ordering (Figure 5A and Figure 3A). The median pseudotime values were higher in aged mice than in young controls (Figure 5B), and the overall trajectory length was extended in aged individuals. Consistent with these observations, Neu06‐IL6 cells were enriched along the late pseudotime trajectory in aged mice but were scarce in young mice (Figure 5B,C).

**FIGURE 5:**
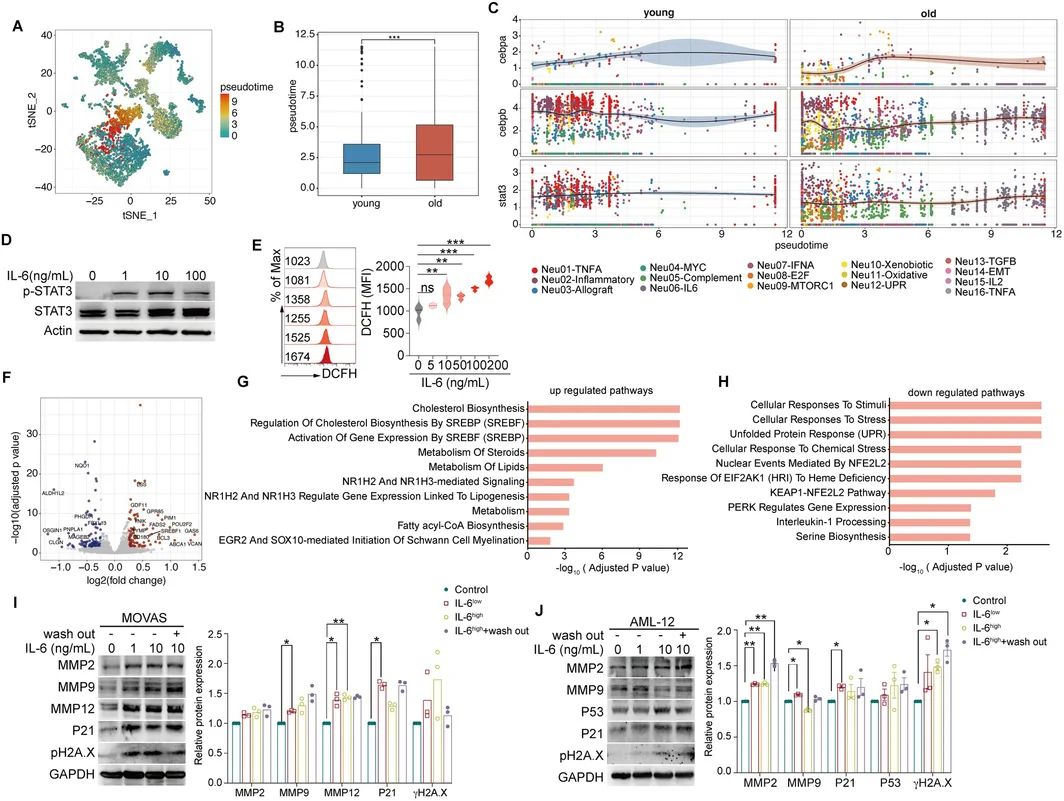
Age‐enriched Neu06‐IL6 neutrophils are associated with inflammatory tissue remodeling. (A) t‐SNE plot illustrating the pseudotime trajectory of neutrophils across age groups. (B) Box plot showing the distribution of pseudotime values among neutrophils from young and aged mice. Boxes represent the interquartile range (IQR), the center line indicates the median, and whiskers extend to 1.5× IQR. (C) Line plot with overlaid dot plots showing the dynamic expression of key transcription factors (TFs) along pseudotime in young and aged neutrophils. Dot colors indicate neutrophil subtypes. Trajectories were fitted using LOESS regression; shaded areas represent standard error. (D, E) STAT3 phosphorylation (D) and DCFH expression (E) were detected with WB and flow cytometry in HL‐60 after IL‐6 stimulation at the indicated dose. *F. volcano* plot showed the differentially expressed genes in HL‐60 after being stimulated with hIL‐6 at 50 ng/mL. (G, H) GSEA pathway analysis showed the signaling up‐ and downregulation in HL‐60 cells stimulated with hIL‐6. (I, J) Cell senescence and matrix metalloproteinase protein expression in MOVAs (I) and AML‐12 (J) after treatment with conditioned medium from HL‐60. GAPDH served as a loading control. Relative protein intensities normalized to control group are indicated. Each dot represents an independent biological replicate. One‐way ANOVA was used in (E). Two‐way ANOVA followed by Tukey's multiple comparisons test was used in (I, J). Data are shown as the mean ± s.e.m., *p < 0.05; **p < 0.01; ***p < 0.001.

The canonical homeostatic differentiation factor CEBPA showed stable expression across pseudotime in both groups. In contrast, CEBPB, which promotes emergency granulopoiesis (Akagi et al. 2008; Avellino and Delwel 2017), increased along the aged pseudotime ordering, suggesting a shift toward an activated, pro‐inflammatory program (Figure 5C). STAT3, a key downstream effector of IL‐6 (Johnson et al. 2018), was up‐regulated in Neu06‐IL6 cells and increased along the aged pseudotime ordering (Figure 5C).

To explore whether IL‐6 induces features resembling the Neu06‐IL6‐associated program, we treated HL‐60 cells, a widely used model for studying neutrophil phenotypic functions (Florentin et al. 2021), with recombinant IL‐6 at varying doses for 24 h. Consistent with the single‐cell transcriptomic findings, IL‐6 stimulation induced STAT3 activation and increased ROS production (Figure 5D,E). Differential gene expression analysis comparing IL‐6‐stimulated and unstimulated HL‐60 cells revealed 96 upregulated and 114 downregulated genes (Figure 5F). Reactome enrichment analysis indicated that cholesterol and lipid metabolism pathways were increased after IL‐6 stimulation (Figure 5G), whereas cellular stress‐response and protein‐homeostasis pathways were reduced (Figure 5H). These results suggest that IL‐6 can promote a transcriptional and functional state with increased inflammatory and oxidative features in this in vitro model.

IL‐6 is a major component of the SASP and can exert its biological effects through both autocrine and paracrine signaling. To determine whether IL‐6 directly affects tissue‐derived cells, MOVAS and AML‐12 cells were treated with IL‐6 for 48 h. Direct IL‐6 exposure increased the percentage of SA‐β‐gal‐positive cells and elevated the mRNA expression of senescence‐ and extracellular matrix‐related genes, as determined by qPCR (Figure S17A–C). Consistently, western blotting showed increased protein expression of senescence‐associated and extracellular matrix‐remodeling molecules, including P21, P53, and MMP2 or MMP9, in both cell types (Figure S17D,E). These results indicate that IL‐6 itself can directly induce stress‐ and senescence‐associated phenotypes in vascular smooth muscle cells and hepatocytes.

To further evaluate the effects of IL‐6‐conditioned neutrophil‐like cells on tissue‐derived cells, conditioned media from IL‐6‐stimulated HL‐60 cells were collected and then applied to MOVAS and AML‐12 cells for 48 h. Treatment with conditioned medium increased the protein and mRNA expression of senescence‐associated and extracellular matrix‐related molecules in both vascular smooth muscle cells and hepatocytes, including P21, P53, γ‐H2A.X, MMP2, and MMP9 (Figure 5I,J, Figure S17F). Notably, this effect persisted when IL‐6 was removed by washout before conditioned‐medium collection, suggesting that it was not solely attributable to residual IL‐6 in the medium.

Collectively, these results indicate that IL‐6 can induce inflammatory and oxidative features in HL‐60‐derived neutrophil‐like cells and further enhance inflammatory and senescence‐associated responses in tissue‐derived cell models.

### 2.6 R6. Deciphering the Potential Factors Associated With Neu06‐IL6 Neutrophils in Aged Tissue Environment

Given the complexity of the aged tissue microenvironment, factors beyond IL‐6 are likely to influence the Neu06 state. To identify candidate signaling inputs associated with the Neu06‐IL6 transcriptional program, we performed NicheNet analysis (Browaeys et al. 2020), a computational framework that predicts ligand‐target relationships by integrating ligand expression, receptor availability, and downstream target gene profiles. The analysis identified several candidate ligand‐receptor axes potentially associated with Neu06‐IL6, including ANGPTL4, BMP2, BMP4, IGF2, and CX3CL1 (Figure 6A). To elucidate the biological significance of the target genes associated with these five receptor‐ligand interactions, we performed Hallmark enrichment analysis. The top three enriched pathways were the P53 signaling pathway, the TNFα signaling pathway, and the TGFβ signaling pathway (Figure 6B). In aged mice, the increased abundance of Neu06 consequently potentially leads to a pronounced aggregate elevation in TNFα, TGFβ, and P53 signaling (Figure 6C and Figure S18A). These results suggest that multiple inflammatory and stress‐response pathways may converge on the Neu06‐IL6 state within aged tissue environments.

**FIGURE 6:**
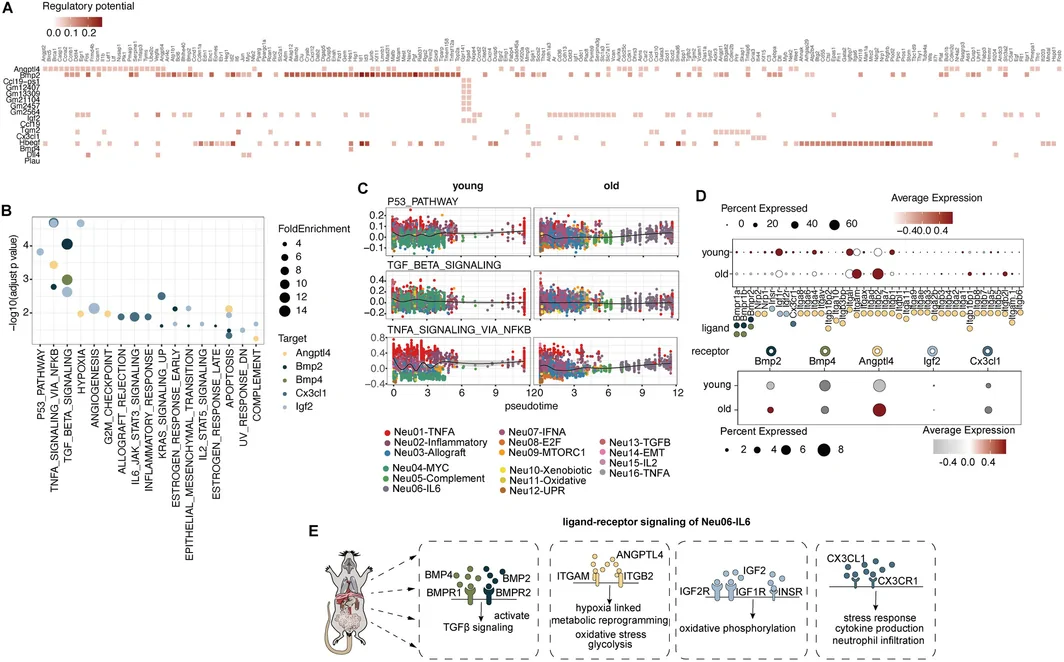
Deciphering the potential factors associated with Neu06‐IL6 neutrophils in aged tissue environment. (A) Heatmap displaying the ligand regulatory potential of the Neu06‐IL6 neutrophil subtype. Rows represent ligands, and columns represent target genes within Neu06‐IL6. Colors indicate row‐scaled regulatory potential scores. (B) Dot plot showing pathway enrichment analysis across various interacting cell types. Dot color represents the ligand identity, while dot size indicates the fold enrichment score of each pathway. (C) Line plots with overlaid dots showing pseudotime dynamics of key pathway module scores: P53, TGF‐β signaling, and TNF‐α signaling in neutrophils from young and aged mice. LOESS regression was used for trajectory fitting; shaded regions indicate standard error. (D) Dot plot showing average expression levels of receptor (top panel) and ligand (bottom panel) genes across age groups. Dot size indicates the proportion of expressing cells; color represents mean gene expression. Statistical significance in pathway enrichment analysis was assessed using the hypergeometric test. (E) Scheme of potential ligand‐receptor signaling of Neu06‐IL6.

Among the candidate ligands predicted to act on Neu06‐IL6, BMP2 and BMP4 are members of the TGF‐β superfamily (Massague 2012). Neu06‐IL6 cells expressed the cognate receptors BMPR1 and BMPR2, suggesting potential responsiveness to BMP2/4 signaling. Predicted BMP2/4 engagement was associated with activation of a TGFβ transcriptional program, which was also prominent in Neu13‐TGFβ and Neu04‐EMT states (Figure S18B). Closer inspection of BMP pathway activity suggested age‐related differences among neutrophil states: aged Neu04‐MYC neutrophils showed reduced BMP signaling relative to young counterparts, whereas aged Neu06‐IL6 neutrophils exhibited heightened BMP pathway activation (Figure S18C). Downstream of BMP2/4, canonical TGF‐β targets were upregulated in Neu06‐IL6, yet non‐canonical stress pathways (JNK, Rho GTPase, PI3K, and MAPK) were not appreciably induced (Figure S18D,E). Metabolic signatures showed subset‐specific and age‐dependent variation (Figure S18F,G). Collectively, these observations nominate the ANGPTL4‐integrin axis as a candidate pathway associated with metabolic and hypoxia‐linked features of Neu06‐IL6 neutrophils.

ANGPTL4, a secreted glycoprotein best known for its pro‐angiogenic activity, signals through multiple integrin receptors (Zuo et al. 2023). Analysis of receptor expression suggested an age‐associated pattern: ITGAM and ITGB2 (CD11b/CD18) were increased in aged mice, whereas ITGA4 and ITGAL (CD49d/CD11a) predominated in young neutrophils (Figure 6D). In Neu06‐IL6 cells, ANGPTL4‐integrin signaling coincided with an increased hypoxia‐related transcriptional program, consistent with a metabolic shift toward glycolysis and oxidative stress management (Figure 6E).

Metabolic signatures showed subset‐specific and age‐dependent variation. Metabolic rewiring downstream of ANGPTL4 extended to broader energy and lipid pathways (Chaube et al. 2023). Xenobiotic and general energy‐metabolism signatures were highest in Neu10‐Xenobiotic and Neu06‐IL6 neutrophils (Figure S18B). Among all subsets, age‐dependent elevation of integrated energy‐metabolism genes was restricted to Neu02‐Inflammatory, Neu13‐TGFβ, and Neu16‐TNFA, which showed a marked increase in aged mice (Figure S18F). Fatty‐acid metabolism exhibited subtler age effects. Significant, albeit modest, differences emerged in Neu02‐Inflammatory, Neu03‐Allograft, and Neu04‐MYC subsets (Figure S18G). The fatty‐acid response module itself displayed a clear dichotomy: expression rose in aged Neu02‐Inflammatory cells but was higher in young Neu08‐E2F cells (Figure S18H). Collectively, these observations nominate the ANGPTL4‐integrin axis as a candidate pathway associated with metabolic and hypoxia‐linked features of Neu06‐IL6 neutrophils.

IGF2, an insulin‐like growth factor, signals primarily through the receptors INSR, IGF1R, and IGF2R (Baxter 2023), whose expression levels did not differ significantly between neutrophils from aged and young mice, nor was IGF2 itself secreted by individual neutrophils (Figure 6E). The IGF2 signaling pathway is closely linked to oxidative phosphorylation, and its activity was notably enriched in Neu04‐MYC, Neu11‐Oxidative, and Neu12‐UPR neutrophil subsets (Figure S18B). Among these, Neu04‐MYC exhibited a significant age‐dependent increase in IGF1R signaling, highlighting enhanced IGF2 responsiveness with aging in this subset (Figure S18I). Conversely, oxidative phosphorylation scores showed significant age‐related changes in Neu02‐Inflammatory, Neu03‐Allograft, and Neu04‐MYC subsets, with Neu04‐MYC showing a marked decline in oxidative phosphorylation activity in aged mice compared to younger controls (Figure S18J). These findings suggest a complex, subset‐specific modulation of IGF2‐related metabolic pathways in neutrophils during aging.

CX3CL1, a key chemokine (Apostolakis and Spandidos 2013), facilitates neutrophil migration and tissue infiltration by binding to its receptor CX3CR1 on neutrophils, notably on the Neu06‐IL6 subset (Figure 6E). Chemokine signaling was prominently expressed in Neu03‐Allograft and Neu12‐UPR neutrophils (Figure S18B). Among these, Neu04‐MYC, Neu06‐IL6, and Neu16‐TNFA displayed significant age‐related differences, with Neu06‐IL6 from aged mice exhibiting elevated CX3CL1 signaling (Figure S18K). Additionally, neutrophil stress response and cytokine production were closely linked to CX3CL1 activity (Lesnik et al. 2003; Schwarz et al. 2010). Neu02‐Inflammatory and Neu04‐MYC subsets showed a strong positive correlation with stress response in aged organisms (Figure S18L). Interestingly, cytokine production scores declined with age in most neutrophil subsets, including Neu01‐TNFA, Neu02‐Inflammatory, and Neu06‐IL6, except for Neu04‐MYC, which maintained elevated cytokine production in aged mice (Figure S18M). These results highlight CX3CL1 signaling as a critical axis modulating neutrophil infiltration, stress responses, and cytokine secretion in aging tissues.

In conclusion, these analyses identify candidate ligand‐receptor and transcriptional pathways associated with Neu06‐IL6 neutrophils in aged tissue environments, including IL‐6/STAT3, BMP2/4‐TGFβ, ANGPTL4‐integrin, IGF2‐related metabolic signaling, and CX3CL1‐CX3CR1. These findings provide a hypothesis‐generating framework for future perturbation studies to define the upstream signals that shape aging‐associated neutrophil states.

### 2.7 R7. Aging Is Associated With an Old‐Enriched HPC11 State and Remodeling of Granulopoietic Programs

In aged organisms, neutrophil remodeling may be influenced not only by extrinsic tissue‐derived inflammatory signals but also by age‐associated changes in bone marrow hematopoiesis. To characterize the bone marrow granulopoiesis landscape during aging, we analyzed single‐cell transcriptomic profiles of bone marrow cells from aged and young mice (GSE17440) and mapped hematopoietic progenitor cell (HPC), granulocytopoietic progenitor (GP), and granulocyte populations by comparison with published bone marrow mononuclear cell reference datasets via a RPCA‐based reference mapping label transfer strategy (Tabula Muris Consortium et al. 2018) (Figure 7A). HPC proportions remained relatively stable with age, whereas GP cells and mature granulocytes were expanded in aged bone marrow (Figure S19A), consistent with age‐associated myeloid‐biased remodeling.

**FIGURE 7:**
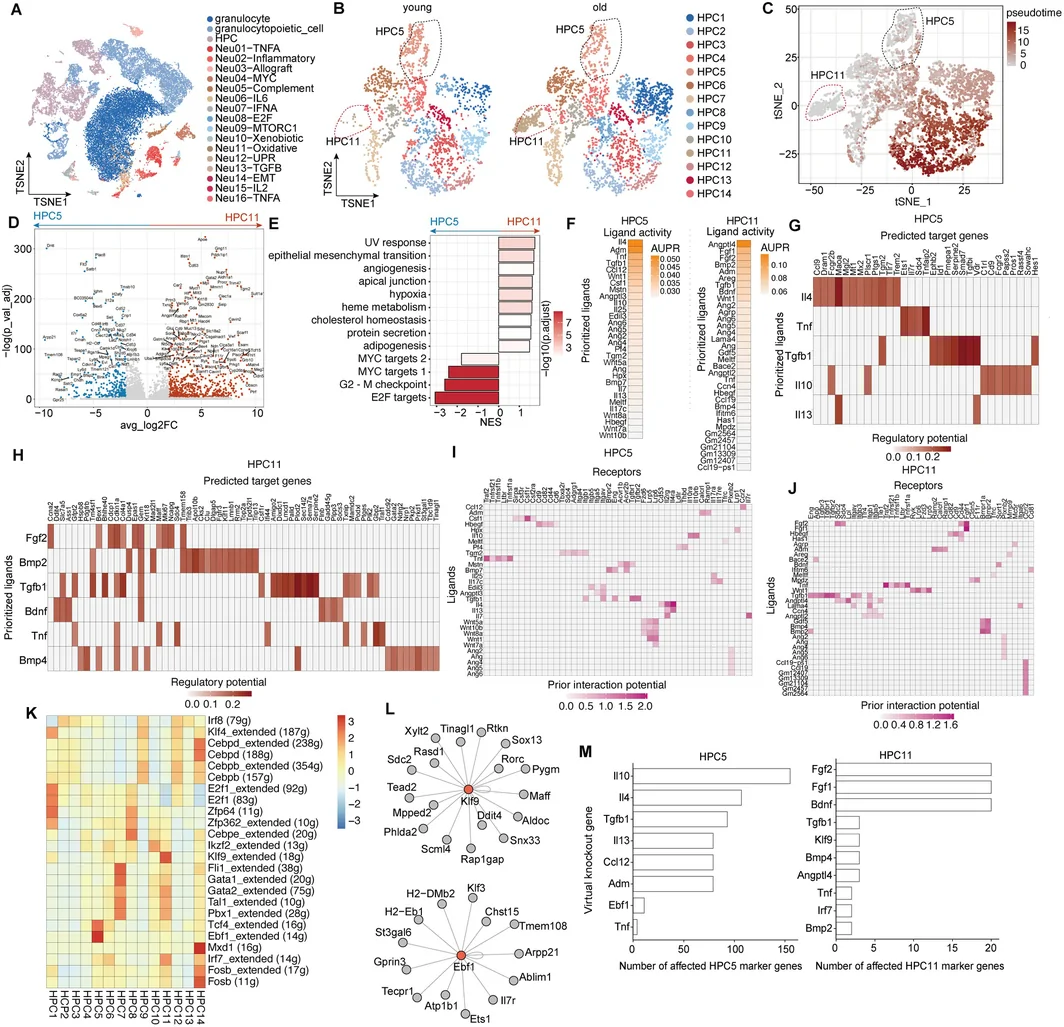
Aging is associated with myeloid‐biased remodeling of bone marrow progenitor and granulopoietic cell states. (A) t‐SNE plot showing integrated neutrophils and bone marrow‐derived cells, including hematopoietic progenitor cells (HPCs), granulocytopoietic cells, and mature granulocytes. (B) t‐SNE plot showing HPC subpopulations in young and aged mice. (C) t‐SNE plot illustrating pseudotime trajectory of HPCs. (D) Volcano plot showing differentially expressed genes between HPC11 and HPC5. Genes with adjusted *p* < 0.05 and |log_2_ fold change| > 1 were considered significant. (E) Bar plot of hallmark pathway enrichment scores based on over‐representation analysis. Bar color reflects −log_10_ adjusted *p* values. (F) Heatmap showing the area under the precision‐recall curve (AUPR) for predicted ligand activities in HPC5 (left) and HPC11 (right). (G, H) Heatmaps depicting regulatory potential of prioritized ligands and their predicted target genes in HPC5 (G) and HPC11 (H). (I, J) Heatmaps showing ligand‐receptor interaction potential in HPC5 (I) and HPC11 (J). (K) Heatmap of row‐scaled regulon activity scores across different HPC subtypes. (L) Transcriptional regulatory network of Klf9 and Ebf1, showing predicted target genes. (M) Bar plots showing the predicted number of marker genes affected by virtual knockout of candidate upstream regulators in HPC5 (left panel) and HPC11 (right panel). Statistical significance in pathway enrichment analysis (E) was determined using the hypergeometric test.

We classified bone marrow cells into HPCs, GPs, and granulocytes and observed subtle but distinct alterations in lineage trajectories between aged and young mice (Figure S19B). Using the neutrophil developmental‐stage signatures (Xie et al. 2020), our bone marrow and granulocyte clusters displayed stage‐specific enrichment patterns consistent with established neutrophil maturation states, supporting the overall validity of our annotations. Early‐stage HPCs clustered primarily in the G0 and G1 phases, corresponding to undifferentiated and early progenitor stages (Figure S19C). GP cells were enriched in the G1–G2 phases, aligning with the pro‐Neu and pre‐Neu developmental stages, where neutrophil identity begins to emerge. Notably, aged mice showed a marked expansion of cells at this intermediate stage.

Granulocytes, corresponding to the G3‐G4 phases, represented immature and maturing neutrophils poised to exit the bone marrow and enter the circulation (Figure S19D). Mapping neutrophil development across these defined stages suggested that age‐related differences were most apparent at later stages of differentiation. While early progenitor proportions were relatively comparable between groups, aged mice showed accumulation of late granulocyte populations from G2 to G4 (Figure S19E). These findings suggest that aging is associated with expanded granulopoiesis output, which may contribute to the increased abundance of neutrophils observed in peripheral tissues.

Given the expansion of granulocyte progenitors and mature granulocytes in aged mice, we further analyzed bone marrow‐derived HPCs and identified 11 transcriptionally distinct HPC subtypes (Figure 7B). Among them, HPC5 was predominantly enriched in young mice, whereas HPC1 and HPC11 were enriched in aged mice, with HPC11 showing minimal representation in the young group. These data indicate an age‐associated shift in progenitor‐cell composition.

To further characterize these populations, we performed pseudotime trajectory analysis using Monocle 3 (Cao et al. 2019). Both HPC5 and HPC11 localized to early stages of hematopoietic differentiation (Figure 7C), suggesting transcriptional differences among early progenitor states during aging. To elucidate the molecular basis of this shift, we conducted differential gene expression analysis between HPC11 and HPC5, identifying 2068 significantly altered genes, including 903 genes upregulated in HPC11 and 1165 in HPC5 (Figure 7D). Notably, HPC5 was enriched for genes associated with lymphoid lineage specification, including *Flt3*, *Satb1*, and *Il2ra*, which promote lymphopoiesis. In contrast, HPC11 displayed elevated expression of myeloid‐associated genes, including *Gata2*, *Car2*, *Prtn3*, and *Rbp1*, which are implicated in granulopoiesis and neutrophil lineage commitment (Figure 7D). Pathway enrichment analysis further supported these distinctions: HPC11 was enriched for angiogenesis, protein secretion, and heme metabolism, whereas HPC5 showed stronger MYC signaling, a central role in lymphoid development and proliferation (Figure 7E). These results suggest that HPC11 represents an aged‐enriched progenitor state characterized by a myeloid‐biased transcriptional program.

To identify candidate extrinsic signals associated with the age‐enriched HPC11 state, we investigated the extrinsic signals influencing the formation of HPC11 and HPC5 using the NicheNet framework. Among the predicted upstream ligands, Angptl4 emerged as a high‐ranking candidate associated with HPC11. In addition, FGF2, a ligand known to promote myelopoiesis (Tian et al. 2022), showed a predicted regulatory influence on HPC11 (Figure 7F). These results predict niche‐derived cues, including Angptl4 and FGF2, may be linked to the age‐enriched HPC11 transcriptional program, providing potential candidate pathways for functional validation.

In contrast, HPC5 was primarily regulated by cytokines such as TNF and IL4, with downstream targets associated with pathways involved in phagocytosis, coagulation, and lymphocyte development (Figure 7G, Table S2). In contrast, ligands influencing HPC11, including FGF2 and BMP2, were predicted to drive biological processes such as angiogenesis and the differentiation of various myeloid lineages, particularly macrophages (Figure 7H). These findings suggest that HPC11 may retain broader myeloid differentiation potential than HPC5.

Receptor analysis of HPC11 revealed elevated expression of FGF‐related receptors and co‐receptors, including *Fgfr1*, *Cd44*, *Sdc2*, and *Sdc4* (Figure 7I,J), supporting potential responsiveness to FGF signaling. SCENIC‐based transcription factor regulatory network analysis (Aibar et al. 2017) showed increased activity of Gata1 and Gata2, both associated with myeloid differentiation (Gao et al. 2015) (Figure 7K). Conversely, HPC5 exhibited high Ebf1 *expression*, a transcription factor essential for B‐cell lineage commitment (Nechanitzky et al. 2013). Additionally, *Klf9*, a transcription factor associated with myeloid cells (Xu et al. 2025; Zhang, Zhang, et al. 2024), was increased in HPC11 (Figure 7L). To further evaluate candidate factors predicted to be associated with the regulation of HPC5 and HPC11, we performed in silico gene perturbation analysis using scTenifoldKnk (Osorio et al. 2022). Virtual knockout of *Il10*, *Il4*, *Tgfb1*, *Il13*, *Ccl12*, and *Adm* had the strongest predicted effects on HPC5 marker genes, whereas virtual knockout of *Fgf2*, *Fgf1*, and *Bdhf* had the strongest predicted effects on HPC11 marker genes (Figure 7M).

Collectively, our results suggest that HPC11 represents an aged‐enriched progenitor state with a myeloid‐associated transcriptional program and is predicted to be responsive to candidate niche‐derived signals such as *Fgf2* and *Angptl4*. These findings provide a potential hematopoietic context for increased aging‐associated neutrophil output.

## 3 Discussion

In this study, we integrated multi‐organ single‐cell RNA‐sequencing datasets from young and aged mice with our own experimental validation to systematically characterize neutrophil remodeling during aging. We identified increased neutrophil accumulation in several aged tissues and multiple aging‐associated neutrophil states, each exhibiting distinct inflammatory, oxidative stress, complement‐related, extracellular matrix remodeling, and IL‐6‐associated transcriptional programs. Cross‐tissue comparison further revealed both shared and organ‐enriched features of these neutrophil states, while analysis of bone marrow datasets suggested that their emergence may be linked to age‐related alterations in neutrophil differentiation and maturation. Selected transcriptional features were also observed in human multi‐organ single‐cell data. Functionally, the in vitro experiments indicate that IL‐6 signaling promotes AAN‐like phenotypic changes in neutrophil‐like cells and that factors released by these cells can induce inflammatory, stress‐associated, and senescence‐associated responses in tissue‐derived cells. Collectively, these findings support an association among aging‐related tissue environments, altered neutrophil states, and tissue‐cell dysfunction, and identify IL‐6 as a potential mediator of this interaction.

To address neutrophil heterogeneity, we constructed an integrated neutrophil expression atlas and sub‐clustered them into 16 distinct transcriptional subtypes. These subtypes could be broadly categorized according to tissue distribution and functional signatures. Several neutrophil populations, including Complement‐associated Neu05, IL6‐expressing Neu06, and E2F‐associated Neu08, were enriched in aged tissues. Tissue‐level reanalysis and replicate‐level statistical approaches supported the recurrence of inflammatory and oxidative neutrophil programs across multiple aged organs. Complement‐related neutrophils (Neu05) may reflect an interaction between complement‐related signaling and aging‐associated neutrophil remodeling. As key components of innate immunity, the complement cascade and neutrophils are tightly interconnected: complement proteins can activate neutrophils, and neutrophils, in turn, can amplify complement activation (Camous et al. 2011; Liu et al. 2022). E2F‐related neutrophils (Neu08) also emerged as a distinct subtype enriched in aged mice. E2F transcription factors are well‐known regulators of cell‐cycle progression and DNA replication (Gaubatz et al. 2000), and their expression has been implicated in modulating neutrophil infiltration and inflammatory responses (Chen et al. 2021). Downstream of E2F, cyclin‐dependent kinases (CDKs), beyond their canonical roles in cell cycle regulation, have been shown to influence mature neutrophil functions, including NET formation, migration, and degranulation (Syahirah et al. 2022).

IL‐6 is a pleiotropic cytokine that plays a complex and critical role in aging (Puzianowska‐Kuznicka et al. 2016). Elevated circulating IL‐6 levels are commonly observed in older individuals and are associated with several age‐related diseases (Puzianowska‐Kuznicka et al. 2016). Consistent with this concept, previous work has proposed that IL‐6 contributes to age‐related vascular pathology by linking extrinsic immune‐cell remodeling with intrinsic tissue and vascular dysfunction, including mitochondrial stress and chronic inflammatory activation (Tyrrell and Goldstein 2021). This framework is particularly relevant to our findings, as the aged tissue microenvironment was associated with expansion of IL‐6‐related neutrophil states and enhanced neutrophil–stromal co‐occurrence across tissues.

IL‐6 also influences neutrophil biology during inflammation by regulating neutrophil mobilization from the bone marrow, tissue migration, and survival, largely through the gp130/STAT3 signaling axis (Hashizume et al. 2011; Fielding et al. 2008). Neutrophils can also produce IL‐6 after stimulation by factors such as Fc‐receptor cross‐linking, G‐CSF, or TLR8 agonists (Ericson et al. 1998; Zimmermann et al. 2015; Zimmermann et al. 2016). In our study, IL‐6 stimulation activated STAT3 signaling and increased ROS production in HL‐60‐derived neutrophil‐like cells. Conditioned medium from IL‐6‐stimulated neutrophil‐like cells further enhanced inflammatory and senescence‐associated responses in MOVAS and AML‐12 cells. Together, these findings support a model in which IL‐6‐associated neutrophil states may amplify inflammatory tissue environments during aging, while direct in vivo causality remains to be established.

The generation of aging‐associated neutrophil states may also be influenced by age‐related hematopoietic remodeling. Our single‐cell transcriptomic analysis of bone marrow revealed expansion of granulopoietic populations and an aged‐enriched HPC11 state with a myeloid‐biased transcriptional program. This observation is consistent with the well‐established myeloid skewing of hematopoiesis during aging. Predicted FGF‐ and Angptl4‐related signaling, together with increased activity of myeloid‐associated transcription factors such as Gata2, may provide candidate mechanisms linking aged bone marrow niches to myeloid‐biased output. However, the functional significance of this process and the mechanisms driving age‐associated atypical neutrophil differentiation remain to be elucidated and require further experimental validation.

Several limitations should be considered. First, many of the single‐cell analyses are based on integrated public or multi‐tissue datasets, and differences in tissue identity, dataset origin, and cell numbers may influence the observed cellular composition and transcriptional states. We therefore performed replicate‐level, tissue‐aware, and pseudobulk analyses where possible, but residual confounding cannot be fully excluded. Second, the term aging‐associated neutrophils refers to neutrophil states enriched in aged organisms and senescence‐associated tissue environments, not necessarily neutrophils undergoing canonical cellular senescence. Third, the in vitro conditioned‐medium experiments indicate inflammatory and senescence‐associated responses in target cells, but they do not prove stable cellular senescence or establish direct in vivo causality. Fourth, NicheNet, ligand–receptor, SCENIC, and pseudotime analyses are computationally inferred and should be interpreted as hypothesis‐generating. Although our findings suggest that aging is associated with increased neutrophil accumulation and AAN‐like inflammatory states, the current in vivo data remain primarily associative and do not establish a direct causal role for these cells in accelerating tissue aging. Future studies using intervention‐based models, such as neutrophil depletion, adoptive transfer, or genetic/pharmacological blockade of AAN‐associated pathways, will be needed to determine whether AAN‐like neutrophils directly contribute to tissue injury, cellular senescence, and age‐related organ dysfunction.

An additional limitation is that the AAN‐like subcluster was defined primarily at the transcriptomic level, and validated protein‐level markers for its prospective identification and isolation remain to be established. Although our single‐cell analyses identified several candidate surface molecules, developing a robust and specific marker panel would require systematic candidate screening, antibody validation, and optimization across multiple tissues and experimental conditions, which extends beyond the scope and primary focus of the present study. Future work should therefore prioritize the comprehensive phenotypic characterization and protein‐level validation of this subcluster, enabling its prospective isolation and direct functional assessment in aging‐related tissue injury.

In conclusion, we identified aging‐associated neutrophil states across multiple tissues and observed increased neutrophil accumulation in several aged organs, accompanied by inflammatory, oxidative stress, complement‐related, and IL‐6‐associated transcriptional programs. Through integrated single‐cell analyses, tissue‐level validation, and in vitro conditioned‐medium experiments, our findings suggest that aging‐associated neutrophil states may contribute to inflammatory tissue environments. These findings provide a resource and a hypothesis‐generating framework for investigating innate immune remodeling during aging.

## 4 Analyzing Detail and Experimental Model

### 4.1 Single Cell RNA‐Seq Data Processing and Integration

scRNA‐seq datasets were analyzed using the Seurat R package (version 5.3.0). To integrate multiple datasets originating from different experimental batches and biological conditions, we employed the anchor‐based integration workflow provided by Seurat. Each dataset was processed independently prior to integration. Briefly, cells with fewer than 100 genes detected, more than 30% mitochondrial gene content, or more than 10,000 UMIs were excluded. Gene expression matrices were normalized using the NormalizeData() function, followed by identification of highly variable features using FindVariableFeatures(). After preprocessing, we selected the integration features using SelectIntegrationFeatures(), typically with 5000 variable genes shared across datasets. The integration anchors were identified using FingIntegrationAnchors(), specifying the list of individual Seurat objects and selecting the first dataset as the reference. Finally, datasets were combined into a single corrected object using IntegrateData().

### 4.2 Dimensionality Reduction and Clustering

The integrated dataset was scaled using ScaleData() again, and principal component analysis (PCA) was performed using RunPCA(). Significant principal components (PCs) were selected based on the elbow plot and JackStraw analysis and used as input for Uniform Manifold Approximation and Projection (UMAP) via RunUMAP() and t‐distributed stochastic neighbor embedding (t‐SNE) via RunTSNE(). Cells were clustered using FindNeighbors() and FindClusters(), with the resolution parameter optimized empirically. The re‐cluster procedure, such as the neutrophil subtype analysis, follows the same procedure (resolution = 0.8).

### 4.3 Cell Type Annotation

In each dataset, cluster‐specific markers were identified using FindAllMarkers() with the Wilcoxon rank‐sum test. Cell type identities were assigned based on canonical marker gene expression and, when available, confirmed by comparison with published single‐cell references.

### 4.4 Cell Percentage Analysis

To investigate differences in cell abundance across samples and conditions, we performed cell percentage analysis using the metadata in the integrated Seurat object. After clustering and annotation, the cell identity and sample information were extracted from the metadata slot of the Seurat object. For each annotated cell type, we quantified the number of cells originating from each individual sample and condition using the group_by() and summarize() functions in R. This allowed us to compare absolute and relative cell proportions across conditions and tissues. For visualization, we employed box plots and stacked proportional bar plots using ggplot2 (version 3.5.2), and statistical comparisons were performed using Fisher's exact test, depending on the sample size and distribution. To account for varying total cell counts per sample, proportions were calculated as the number of cells of a given type divided by the total number of cells in the respective sample.

### 4.5 Differential Neutrophil Abundance Analysis Using scCODA

Differential neutrophil abundance between young and aged samples was assessed using single‐cell Compositional Data Analysis (scCODA), a Bayesian framework designed for compositional analysis of single‐cell data (Buttner et al. 2021). Analyses were performed independently for each tissue to account for tissue‐specific differences in neutrophil composition. For each tissue, cell counts were aggregated at the biological‐sample level to generate a sample‐by‐cell‐type count matrix. Bayesian inference was performed using Hamiltonian Monte Carlo sampling. Posterior effects were estimated for each cell population, and neutrophils with credible non‐zero effects according to the scCODA model‐based inclusion criterion were considered to exhibit significant age‐associated compositional changes.

### 4.6 Pseudobulk Differential Expression Analysis of Aged and Young Neutrophils

To minimize potential inflation of statistical significance arising from cell‐level differential expression testing, we performed pseudobulk differential expression analysis using biological replicates as the unit of inference. Neutrophils were first extracted from the integrated single‐cell dataset and then grouped by biological sample and age group. Raw UMI counts from all neutrophils originating from the same biological sample were aggregated to generate sample‐level pseudobulk expression profiles. Genes expressed in fewer than three pseudobulk samples or with low overall abundance were excluded prior to analysis. Differential expression between aged and young neutrophils was then assessed using the DESeq2 package (v1.46.0). Size factors were estimated using the median‐of‐ratios method, and gene‐wise dispersion parameters were fitted using the standard DESeq2 negative binomial framework. Statistical significance was evaluated using Wald tests. Genes with an adjusted *p* value < 0.05 (Benjamini–Hochberg correction) and an absolute log2 fold change > 0.5 were considered differentially expressed. To investigate biological processes associated with aging, significantly upregulated genes in aged and young neutrophils were subjected to pathway enrichment analysis using the Hallmark gene sets from the Molecular Signatures Database (MSigDB v2024.1). Enrichment significance was calculated using over‐representation analysis implemented in clusterProfiler (v4.14.6), and pathways with a false discovery rate (FDR) < 0.05 were considered significantly enriched.

### 4.7 Patient‐Level Pseudobulk Module Score Analysis

To assess signature activity across patient groups, we calculated module scores from single‐cell RNA‐seq data and aggregated them at the patient level. For each predefined gene signature, genes detected in the expression matrix were retained, and a module score was computed for each cell based on the average normalized expression of the corresponding signature genes. To avoid treating individual cells as independent biological replicates, module scores were summarized for each patient within each annotated cell population by calculating the mean score across all cells from the same patient and cell type. These patient‐level pseudobulk module scores were then used for statistical comparison across clinical or experimental groups. Each patient contributed one aggregated value per cell type and module, thereby preserving the patient as the unit of biological replication.

### 4.8 Hallmark Pathway Enrichment Analysis

To interpret the biological significance of neutrophils' DEGs, we performed pathway enrichment analysis using the Hallmarker gene sets from the Molecular Signatures Database (MsigDB, v25.1.0). DEGs identified by Seurat's FindMarkers() function were ranked by average log fold change. Enrichment analysis was conducted using the clusterProfiler R package (version 4.14.6) and the msigdbr package to retrieve human Hallmarker gene sets. Gene set enrichment was performed using the enricher() or GSEA() function, depending on whether a ranked gene list or DEG set was used. Enrichment results were filtered based on false discovery rate (FDR) < 0.05. Significant pathways were visualized using bar plots generated with ggplot2.

### 4.9 Pathway Signature Scoring and Visualization

To assess the activity of specific biological pathways at the single‐cell level, we calculated module scores using the AddModuleScore() function in the Seurat package. Some gene sets representing pathway signatures (ROS generation, degranulation, phagocytosis, and maturation) were obtained from the MsigDB. Some gene sets representing pathway signatures (NADPH oxidation, ROS production, aging clock genes, bone marrow genes, and different stages of neutrophil signatures) were obtained from Gullotta et al. (2023) and Xie et al. (2020). The NETs‐associated gene signature was derived from Wang et al. (2020). Gene lists corresponding to selected pathways were input into AddModuleScore(), which computes an average expression score for each cell based on the expression pathway genes, relative to control gene sets matched by expression bin. The resulting module scores were stored in the metadata of the Seurat object and compared across different age groups. Visualization of pathway activity was performed using box and violin plots generated with ggplot2, enabling comparison of pathway activity distributions at the single‐cell level. Statistical significance was evaluated using the Wilcoxon rank‐sum test for pairwise comparisons, and adjusted *p* values were reported where appropriate.

### 4.10 BM Proximity and Cellular Aging Scoring

BM proximity scores were calculated using a previously reported single‐cell scoring strategy for neutrophil maturation states. Briefly, we used a published BM proximity gene signature associated with bone marrow‐derived immature neutrophil programs, including Mmp8, Mmp25, Lcn2, Olfm4, Itgb2, Fpr1, Ltf, and Camp. Module scores were calculated using the Seurat AddModuleScore function. The resulting BM proximity score reflects the relative expression of this bone marrow‐associated immature neutrophil signature in each cell or cluster. This score was used to estimate transcriptional similarity to immature bone marrow‐associated neutrophil states, rather than anatomical proximity to the bone marrow or the precise time elapsed since neutrophil egress. Cellular aging scores were calculated using the corresponding published aging‐related neutrophil gene signature with the same module‐scoring approach.

### 4.11 Harmony Integration of Single‐Cell RNA‐Seq Data

Principal components were integrated using Harmony, with sequencing batch specified as the covariate. Harmony‐corrected embeddings were used to construct a shared nearest‐neighbor graph, perform unsupervised clustering, and generate UMAP visualizations. Clusters were annotated based on canonical lineage markers and previously reported immune cell marker genes.

### 4.12 Clustree‐Based Clustering Stability Analysis

To evaluate the robustness of neutrophil clustering and determine an appropriate clustering resolution, we performed a clustering stability analysis using the clustree R package. Briefly, graph‐based clustering was conducted across a series of resolution parameters ranging from 0.1 to 1.5 using the shared nearest‐neighbor graph generated from the integrated single‐cell RNA‐seq dataset. Cluster relationships across consecutive resolutions were visualized with clustree, in which nodes represent clusters at each resolution and edges indicate the proportion of cells shared between clusters across adjacent resolutions. We assessed cluster stability based on the consistency of cluster inheritance, the presence of clear parent–child relationships, and the absence of excessive fragmentation at higher resolutions. Based on the clustree topology, together with marker gene specificity and biological interpretability, a resolution of 0.8 was selected for downstream analyses. This resolution provided a balanced clustering structure, preserving major neutrophil subtypes while avoiding artificial subdivision of transcriptionally similar populations.

### 4.13 Leave‐One‐Tissue‐Out Sensitivity Analysis

To assess whether the identified aging‐associated neutrophil programs were driven disproportionately by any single tissue, we performed a leave‐one‐tissue‐out (LOTO) sensitivity analysis. Briefly, the integrated single‐cell dataset was iteratively reanalyzed after excluding all cells derived from one tissue at a time (blood, intestine, liver, lung, vascular tissue, heart, kidney, or brain). For each iteration, the remaining cells were subjected to the same preprocessing, integration, clustering, and downstream analyses used in the full dataset. To evaluate the robustness of aging‐associated neutrophil states, we recalculated cluster composition and co‐occurrence meta‐programs for each LOTO dataset. The similarity between the full dataset and each LOTO iteration was assessed by comparing the relative abundance of neutrophil subtypes. In addition, the recurrence of co‐occurrence meta‐programs was quantified across iterations to determine whether specific tissue compartments disproportionately contributed to the observed cross‐tissue programs. The persistence of aging‐associated neutrophil signatures following removal of individual tissues was interpreted as evidence that the identified programs represent broadly conserved features of neutrophil aging rather than tissue‐specific effects. Conversely, substantial changes in subtype composition or pathway activity following exclusion of a particular tissue were interpreted as evidence of tissue‐specific contributions to the integrated atlas.

### 4.14 Quantification of CM Recurrence Using the Jaccard Index

To quantify the recurrence of each full‐data CM after removal of an individual tissue, each reference CM was compared with all modules reconstructed in the corresponding LOTO iteration. For a reference CM and a LOTO‐derived module, membership similarity was quantified using the Jaccard index: where represents the number of cell types shared between the full‐data CM and the LOTO‐derived module, and represents the total number of unique cell types present in either module. For each reference CM, the LOTO‐derived module with the highest Jaccard index was designated as its best‐matched module. The corresponding maximum Jaccard index was used as the measure of CM recurrence for that tissue‐removal iteration. A Jaccard index of 1 indicates identical membership between the full‐data and LOTO‐derived modules, whereas values approaching 0 indicate little or no membership overlap. CM recurrence was summarized across all tissue‐removal iterations to evaluate whether individual modules represented broadly recurrent co‐abundance structures or were sensitive to exclusion of particular tissues.

### 4.15 Quantification of Tissue Dependence of CM Stability

To identify CMs whose stability was disproportionately influenced by a specific tissue, we calculated a tissue‐dependency score for each CM–tissue pair. For a given CM and tissue, the dependency score was defined as: where is the Jaccard similarity of CM after removal of tissue, and the first term represents the median Jaccard similarity for the same CM across all other LOTO iterations. Positive dependency scores therefore indicate that removal of the specified tissue reduced CM recurrence relative to its typical stability across other tissue‐removal conditions, consistent with greater dependence of the module on that tissue. Scores near zero indicate little tissue‐specific effect, whereas negative values indicate that CM recurrence after removal of that tissue was greater than the median recurrence observed following removal of other tissues.

### 4.16 Cell Composition Correlation and Cellular Module Analysis

To investigate relationships between cell type distributions across tissues, we first quantified the relative abundance (percentage) of each cell type within each tissue and sample. Cell percentages were calculated from the Seurat metadata by dividing the number of cells of a given type by the total number of cells per tissue. This generated a cell‐type and tissue‐matrix matrix with proportional cell abundances. To identify co‐occurrence patterns between cell types, we computed pairwise Pearson correlation coefficients across tissues based on the cor() function with method set to Pearson. The resulting correlation matrix was used to assess the similarity of cell type abundance profiles across tissues. We then applied hierarchical clustering using complete‐linkage on the correlation matrix to group cell types into cellular modules exhibiting coordinated variation across tissues. Clustering and visualization of the correlation matrix were performed using the p‐heatmap package. The clustering patterns were used to define cellular modules, reflecting shared tissue distribution trends and potential coordinated biological functions.

### 4.17 Cell‐Chat Analyzing

To infer cellular communication networks among cellular modules, we used the Cell‐Chat R package (version 2.1.2), which predicts ligand‐receptor interactions from single‐cell transcriptomic data. The normalized and annotated Seurat object was first converted into a Cell‐Chat object using the “creatCellChat” function. The group by parameter was set to the cellular model (CM) labels, previously defined through hierarchical clustering of cell‐type abundances. Cell‐Chat's built‐in human ligand‐receptor interaction database (CellChatDB.mouse) was used to identify signaling interactions. After preprocessing and filtering lowly expressed genes, the communication probability between CM pairs was computed using the computeCommunProb function, and pathway‐level communication was inferred using computeCommunProbPathway(). We specifically focused on the CXCL and CCL signaling pathways, given their relevance to chemokine‐mediated immune cell recruitment and inflammation. The inferred interactions were visualized using circle plots, hierarchical network diagrams, and heatmaps, highlighting both the strength and directionality of communication between modules. The overall communication probability, incoming and outgoing signaling strength, and centrality measures were used to characterize the dominant sender and receiver cells within the CXCL and CCL pathways.

### 4.18 Pseudotime Inference of Neutrophils Using Monocyte Reference Trajectory

To explore the developmental or activation continuum of neutrophil subtypes in relation to monocyte‐driven signaling, we inferred pseudotime trajectories of neutrophils using monocytes as a reference population. Monocytes and neutrophils were first extracted from the integrated Seurat object using cell type annotations; we then performed trajectory inference with Monocle3 (version 1.3.7), using a design that anchors pseudotime progression to monocyte positioning. Dimensionality reduction was conducted using PCA followed by UMAP, and cell clustering was performed with the Leiden or Louvain algorithm, depending on the tool used. To construct the trajectory, monocytes were designated as the root (starting) node, based on the hypothesis that they represent an upstream immune activation state. Neutrophil subtypes were then ordered along a continuous pseudotime trajectory relative to this root.

### 4.19 Ligand‐Receptor Interaction Analysis With NicheNet

To identify potential ligand‐receptor interactions driving neutrophil subtype‐specific gene expression, we applied the NicheNet framework (version 2.2.0), which predicts ligand activities based on downstream gene expression changes in target cells. The analysis was performed using the nichenetr R package in combination with Seurat objects containing annotated neutrophil subtypes. We defined receiver cells as specific neutrophil subtypes of interest, and sender cells as all other immune and stromal populations identified in the dataset. The expressed genes in sender and receiver cells were determined using Seurat's AverageExpression() and FilterGenes() functions, typically requiring expression in at least 10% of cells within a group. A list of DEGs in the receiver neutrophil subtype of interest to other neutrophil subsets (Neu06‐IL6 vs. other neutrophil subsets, HPC11 vs. HPC5). NicheNet's built‐in ligand‐receptor network (based on prior knowledge of signaling and gene regulatory interactions) was used to compute ligand activity scores, ranking ligands based on their ability to predict the observed DEG profile in the receiver population. Top‐ranked ligands were selected based on Pearson correlation coefficients between predicted and observed gene expression. Corresponding receptors expressed in the neutrophil subtypes were also extracted to support the inferred interactions. Visualization was performed using heatmaps to highlight key signaling pathways and ligand‐receptor pairs.

### 4.20 Cell‐Level Differential Gene Expression Analysis

To identify DEGs in neutrophils between different age groups, we subset the integrated Seurat object to retain only neutrophil populations based on cell type annotation. Differential expression analysis was performed using the FindMarkers() function in Seurat (version), comparing cells from different conditions (age vs. young). The Wilcoxon rank‐sum test was used as the default statistical method; only genes expressed in at least a minimum fraction of cells (default, min.pct = 0.1) in either group were considered. Genes with an average log fold change greater than 0.5 and an adjusted *p* value (Benjamin‐Hochberg method) less than 0.05 were considered significantly differentially expressed. Prior to DEG analysis, the dataset was normalized and scaled using Seurat's standard preprocessing pipeline. DEGs were visualized using volcano plots and heatmaps, and further interpreted through functional enrichment analysis.

### 4.21 Gene Regulatory Network Inference With SCENIC

To infer gene regulatory networks (GRNs) underlying neutrophil subtype heterogeneity, we employed the SCENIC workflow (Single‐Cell rEgulatory Network Inference and Clustering; version 1.3.1). Neutrophil populations were first subset from the integrated Seurat object based on cell‐type annotations. The resulting gene expression matrix was exported and processed using R SCENIC, which involves three main steps: GRN inference, regulon prediction, and regulon activity scoring. First, transcription factor (TF)‐target co‐expression modules were identified using GENIE3, based on the expression matrix filtered for highly variable genes. Next, each module was refined by motif enrichment analysis using a species‐specific cis‐regulatory motif database (mm10_refseq‐r80_500bp_up_and_100bp_down_tss.mc9nr.feather).

Regulon activity scoring across individual cells was calculated using AUCell, which computes the Area Under the Curve (AUC) for each regulon's gene set within the ranked expression profile of each cell. Regulon AUC scores were integrated back into the neutrophil Seurat object, enabling visualization across neutrophil subtypes and experimental groups. To identify transcriptional regulators associated with aging, we performed differential regulon activity analysis between young and aged groups using the Wilcoxon rank‐sum test on regulon AUC scores. Visualization of regulon activity was conducted using pheatmap (version 1.0.13).

### 4.22 Pathway and Critical TFs Along Pseudotime

To investigate the dynamic regulation of biological pathways during neutrophil progression, we assessed the relationship between pathway activity scores (P53, TGFB, and TNFA), critical TFs (*Cebpa*, *Cebpb*, and *Stat3*) and pseudotime values using locally weighted scatterplot smoothing (LOESS) regression. Pathway activity scores were computed for each cell using Seurat's AddModuleScore () function based on curated gene sets, and pseudotime values were derived as described above using Monocle3. For each pathway, we modeled the trend of its activity across pseudotime using LOESS regression implemented via the geom_smooth (method = “loess”) function from the ggplot2 package. This approach allowed visualization of nonlinear trends and smooth changes in pathway activity along the pseudotime continuum. Smoothed trajectories were plotted with 95 confidence intervals to highlight statistically robust patterns.

### 4.23 Reference Mapping and Cell‐Type Annotation of Bone Marrow Single‐Cell Data

The reference and query datasets were processed using Seurat. Briefly, raw count matrices were independently quality‐controlled, normalized, and scaled. Highly variable genes were identified within each dataset and used for downstream integration. To minimize technical differences between datasets while preserving biological variation, we applied Seurat's reciprocal principal component analysis (RPCA)‐based anchor integration strategy.

For RPCA‐based integration, principal component analysis was first performed independently on the reference and query datasets using shared variable features. Integration anchors between the two datasets were then identified using the FindIntegrationAnchors function with the reduction parameter set to “rpca”. The identified anchors were used to align the query dataset with the reference atlas and project both datasets into a shared transcriptional space. This approach allows transcriptionally similar cells from the query and reference datasets to be co‐embedded while reducing dataset‐specific batch effects.

Cell‐type labels in the query bone marrow dataset were assigned based on their alignment with annotated reference populations in the integrated space. Cluster identities were further verified by examining canonical marker gene expression for major bone marrow lineages and neutrophil maturation states. Ambiguous clusters or clusters with mixed marker expression were manually inspected and annotated according to the dominant reference‐matched population and canonical lineage markers.

This RPCA‐based reference mapping and label transfer strategy enabled annotation of our bone marrow single‐cell atlas using an external, well‐characterized reference while maintaining the transcriptional structure of the query dataset. The final cell‐type annotations were used for downstream analyses of bone marrow immune composition, neutrophil maturation states, and aging‐associated transcriptional programs.

### 4.24 In Silico Perturbation Analysis Using scTenifoldKnk

Candidate‐gene perturbation analysis was performed using scTenifoldKnk. Normalized single‐cell expression matrices were extracted from selected cell populations after quality control and annotation. Candidate genes were selected based on ligand–receptor analysis, transcription factor enrichment, and relevance to age‐associated neutrophil or progenitor‐cell remodeling. For each candidate gene, scTenifoldKnk reconstructed a single‐cell gene regulatory network from the unperturbed expression matrix, generated a virtual knockout by removing the outgoing regulatory edges of the target gene, and compared the original and perturbed networks using manifold alignment. Genes with large manifold distance scores were considered strongly affected by the virtual perturbation and were used for downstream pathway enrichment and signature‐level interpretation. Because this approach is computational and inference‐based, the results were interpreted as hypothesis‐generating and require experimental validation.

### 4.25 Mice and Cell Line

Young mice were 2–4 months old, and old mice were 18–20 months old. The AML‐12 (cultured with F12+10% FBS+ penicillin/streptomycin) and HL‐60 (cultured with DMEM+20% FBS+ penicillin/streptomycin) cell lines were purchased from Procell; MOVAs (cultured with DMEM+10% FBS+ penicillin/streptomycin) were purchased from ATCC.

### 4.26 Histological Staining (Hematoxylin–Eosin (HE) and Immunofluorescence (IF))

The fresh tissue was fixed with 4% paraformaldehyde for 24 h and then sliced into 4 μm‐thick sections after paraffin embedding. The tissue slices were then deparaffinized and rehydrated with diluted ethanol and distilled water. Hematoxylin solution (Harris) and eosin solution were used for HE staining, and observations were performed with a Nexcope (NE900) microscope. The fixed vascular tissue was embedded in an OCT embedding medium (Sakura, Japan) and sliced into 5 μm thick transverse sections. HE, EVG, and Oil Red O staining were performed as previously reported. Frozen sections were permeabilized with 0.1% Triton X‐100 (BioFroxx 1139ML100) for 15 min. Non‐specific binding sites were blocked with 10% serum from the secondary antibody species at 37°C for 30 min. The primary antibody working solution was prepared and incubated overnight at 4°C. Thawed sections were subsequently incubated with the secondary antibody solution at 37°C for 1 h. Subsequently, the sections were stained with a DAPI solution for 5 min in the dark to label the nuclei, followed by washing with TBST. The samples were imaged using a confocal laser‐scanning microscope (Nikon C2+, Tokyo, Japan). Image‐Pro Plus 7.0 software (Media Cybernetics, USA) was used for image analysis.

### 4.27 Neutrophil Isolation and Co‐Culture

Bone marrow from mice was isolated, followed by neutrophil isolation via Percoll density gradient centrifugation (17‐0891‐09, GE). A million neutrophils were cultured in complete RPMI medium (RPMI +10% FBS + penicillin/streptomycin) and stimulated as indicated. After 4 h of culture, neutrophils were stained with CytoX (Invitrogen), and the medium was collected for incubation with the MOVAS or AML‐12 cell line. Stimulated cells were collected for WB and qPCR.

### 4.28 Western Blot

Cells were washed with PBS, and RIPA was used for protein extraction. Protein density was calculated using the BCA method, and 10 μg of protein was loaded onto an SDS‐PAGE gel, followed by transfer to a PVDF membrane (0.2 μm, GE). The membrane was blocked with 5% milk for 1 h at room temperature. The first antibody was incubated at 4°C overnight. The rabbit or mouse second antibody was incubated for 1 h at room temperature after being washed 3 times with TBS containing 0.1% Tween. MMP2, MMP9, MMP12, P16, and GAPDH were purchased from Proteintech; P53 was purchased from Affinity; P21 was from Santa Cruz. STAT3 (79D7) and p‐STAT3 (Tyr705, D3A7) were purchased from Cell Signaling Technology.

### 4.29 Quantitative PCR

Total RNA was extracted using RNA isolator Total RNA Extraction Reagent (Vazyme) according to the instructions. 1 μg RNA was used for cDNA synthesis with HiScript IV All‐in‐One Ultra RT SuperMix (Vazyme). Quantitative PCR was performed with Taq Pro SYBR qPCR Master Mix (Vazyme). Gene expression was normalized to the house gene (β‐actin). Relative gene expression was calculated using 2‐DDCT, and the fold change relative to the control group was presented. Primer sequence: Il6 (F‐ACCACGGCCTTCCCTACTTC, R‐TTGGGAGTGGTATCCTCTGTGA). Il1a (F‐AAGAGACCATCCAACCCAGATC, R‐CCTGACGAGCTTCATCAGTTTG). Il1b (F‐TCAGGCAGGCAGTATCACTCA, R‐CACGGGAAAGACACAGGTAGCT). Tnf (F‐GTTCTGCAAAGGGAGAGTGG, R‐GCACCTCAGGGAAGAGTCTG). Ifn (F‐TTGGCTTTGCAGCTCTTCCT, R‐ATGACTGTGCCGTGGCAGTA). Mmp3 (F‐TTGACGATGATGAACGATGGA, R‐GAGCAGCAACCAGGAATAGGTT). Mmp9 (F‐TGAGTCCGGCAGACAATCCT, R‐CCCTGGATCTCAGCAATAGCA). Mmp12 (F‐GTGCCCGATGTACAGCATCTT, R‐GGTACCGCTTCATCCATCTTG).

### 4.30 Cell Senescence β‐Gal Staining

Cells were washed twice with PBS after stimulation under the indicated conditions. 2% formaldehyde was used to fix the cells. β‐gal staining solution was made following the instructions (Cell Signaling Technology, 9860). Cells were washed with PBS after fixation and stained with β‐gal for at least 4 h before being imaged with a phase‐contrast microscope. The images were analyzed with ImageJ software.

## Author Contributions

Yuman Wang and Teding Chang designed the study and wrote the manuscript. Teding Chang performed the analysis of the single‐cell transcriptomic data. Yuman Wang carried out the experiments. Yangguang Fu, Guangyu Liang, and Man Liu contributed to data interpretation and discussion. Huicheng Liu, Le Zhang, and Jinhua Yan were involved in revising the project. Anding Liu and Cuntai Zhang critically revised and supervised the manuscript. All authors reviewed and approved the final version of the manuscript.

## Funding

This work was supported by National Natural Science Foundation of China, 82271596, 82501916; Hubei Provincial Natural Science Foundation of China, 2025AFB133; the Postdoctoral Project of Hubei Province, 2025HBBSHCXB035; Noncommunicable Chronic Diseases‐National Science and Technology Major Project, N2024ZD0539100.

## Conflicts of Interest

The authors declare no conflicts of interest.

## Supporting information


**Figure S1:** Neutrophil infiltration is increased in multiple organs of aged mice. (A) t‐SNE plot displaying the distribution of single‐cell transcriptomes across various tissues in young and aged mice. (B) Dot plot showing the expression of canonical marker genes used for cell type annotation across tissues. Dot color indicates the average expression level of each marker gene, and dot size represents the percentage of cells expressing the marker within each cluster. (C) Stacked bar plot illustrating the relative proportions of major cell types across tissues in young versus aged mice. Bar segments are colored by cell type, and the size of each segment reflects the percentage of that cell type within the tissue.
**Figure S2:** Morphology of tissues from young and old mice. (A) HE stains of multiple organs (lung, intestine, kidney, liver, brain, and heart) from young and old mice. Bar = 20 μm.
**Figure S3:** Aging‐associated neutrophils (AANs) exhibit enhanced pro‐ inflammatory activity across multiple tissues. (A–D) Violin plots showing module scores for ROS generation (A), glycolytic activity NADPH score (B), NETs formation (C), and degranulation (D) in neutrophils from different tissues in young versus aged mice. The shape of each violin reflects the distribution density of scores; embedded box plots display the interquartile range (IQR) and median. Statistical significance was assessed using the Wilcoxon rank‐sum test. ns, not significant.
**Figure S4:** Resolution optimization and clustering stability analysis identify age‐associated neutrophil subpopulations. (A) Uniform Manifold Approximation and Projection (UMAP) visualization of Harmony‐integrated neutrophils from multiple tissues. Graph‐based clustering identified 16 neutrophil populations (Neu01–Neu16), which are colored according to cluster identity. Cluster numbers are overlaid on the corresponding cellular populations. Batch correction and data integration were performed using Harmony to minimize tissue‐specific efifects while preserving biological variation. (B) UMAP visualization of the same Harmony‐integrated neutrophils colored by datasets, including blood, heart, intestine, kidney, liver, lung, and vascular tissues. Cells from different datasets were broadly intermingled within clusters, indicating successful integration and shared neutrophil transcriptional programs across organs. (C) UMAP visualization colored by annotated neutrophil states. Cluster identities were assigned based on the dominant transcriptional programs identified by differential gene expression and pathway enrichment analyses. Annotated neutrophil states included TNFA signaling (Neu01 and Neu16), inflammatory neutrophils (Neu02), allograft rejection–associated neutrophils (Neu03), MYC signaling (Neu04), complement‐associated neutrophils (Neu05), IL‐6 signaling (Neu06), IFNA response (Neu07), E2F target programs (Neu08), MTORC1 signaling (Neu09), xenobiotic metabolism (Neu10), oxidative phosphorylation (Neu11), unfolded protein response (UPR; Neu12), TGF‐β signaling (Neu13), epithelial–mesenchymal transition (EMT; Neu14), IL‐2 signaling (Neu15), and TNFA signaling (Neu16). Data were integrated using Harmony following standard single‐cell RNA‐sequencing preprocessing, dimensionality reduction, and graph‐based clustering. Cell‐state annotations were assigned based on the dominant enriched Hallmark pathways within each cluster. (D) Clustree visualization illustrating cluster transitions across a range of graph‐based clustering resolutions (0.1–1.5) following dimensionality reduction and neighborhood graph construction. Each node represents a cluster at a given resolution, with node size proportional to the number of cells and color indicating cluster abundance. Edges connect cells shared between clusters across adjacent resolutions, enabling visualization of cluster stability and subdivision as resolution increases. (E) Relationship between clustering resolution and the total number of identified neutrophil clusters. Increasing resolution led to a progressive subdivision of cellular populations, with the number of clusters rising from 11 at low resolution to 22 at the highest resolution tested. (F) Mean silhouette score across clustering resolutions, used to evaluate cluster compactness and separation. The highest silhouette scores were observed at intermediate resolutions, indicating improved cluster stability and biological coherence compared with higher‐resolution over‐partitioning. (G) Uniform manifold approximation and projection (UMAP) visualization of neutrophils clustered at representative resolutions (0.4, 0.6, 0.8, and 1.0). Cells are colored by cluster identity and labeled with their cluster numbers. Increasing clustering resolution progressively resolved additional neutrophil subpopulations while preserving the overall manifold structure. Based on the combined assessment of cluster stability, silhouette score, and biological interpretability, the selected resolution was used for downstream analyses. Data were analyzed using graph‐based community detection following single‐cell RNA sequencing preprocessing, dimensionality reduction, and nearest‐neighbor graph construction. Cluster robustness was assessed by clustree analysis and silhouette scores across multiple clustering resolutions.
**Figure S5:** Functional characterization of aging‐related neutrophils in vascular tissue. (A) UMAP plot showing neutrophil subsets in vascular tissue, with colors representing distinct neutrophil populations. (B) Bar plot showing the relative proportions of neutrophil subsets. Bar height indicates the percentage of cells, and colors denote different subsets. (C) Heatmap displaying the top 10 marker genes for each neutrophil subset. Colors in the bar indicate subsets, while heatmap colors represent normalized gene expression levels. (D) Dot plot showing the top three marker genes for each neutrophil subset. Dot size indicates the percentage of cells expressing the gene, and color reflects average expression levels. (E) Dot plot showing the similarity scores of Xie's neutrophil signatures across different subsets. Both color and dot size represent mean similarity scores. (F) Violin plot showing the maturation scores of neutrophil subsets. Embedded boxplots indicate the median values, with whiskers extending to the minimum and maximum non‐outlier values. (G) Heatmap (left) showing the expression of bone marrow similarity–related genes in individual cells, and violin plot (right) showing the corresponding similarity scores across subsets. Embedded boxplots display median scores, with whiskers denoting non‐outlier ranges. (H) UMAP plot (left) showing the distribution of aging‐related neutrophils, with colors representing cell origins from mice of different ages. Bar plot (right) showing the proportion of aging‐related neutrophils in each subset. (I) Heatmap showing the expression of aging‐related clock genes across cells. Color scale represents expression intensity. (J) UMAP plot (left) showing aging score distribution across neutrophils. Violin plot (right) showing aging scores across subsets. Embedded boxplots indicate median values with whiskers denoting non‐outlier ranges. (K) Violin plot showing neutrophil extracellular trap (NET) formation scores across subsets. Embedded boxplots indicate medians with whiskers denoting non‐outlier ranges. (L) Violin plot showing reactive oxygen species (ROS) generation scores (left) and NADPH oxidase activity scores (right) across neutrophil subsets. Embedded boxplots indicate medians with whiskers denoting non‐outlier ranges. (M) Violin plot showing phagocytosis scores across neutrophil subsets, with embedded boxplots showing median values and whiskers denoting non‐outlier ranges. (N) Violin plot showing degranulation scores across neutrophil subsets, with embedded boxplots showing median values and whiskers denoting non‐outlier ranges. (O) Heatmap showing transcription factor activity across neutrophil subsets as determined by SCENIC analysis. Color represents relative transcription factor activation levels.
**Figure S6:** Functional characterization of aging‐related neutrophils in liver tissues. (A) The overall design of the mouse single‐cell liver analysis. (B) UMAP plot showing cell populations in liver, with colors representing distinct cell populations. (C) UMAP plot showing neutrophil subsets in liver of different ages, with colors representing distinct cell populations (left panel). Bar plot showing the relative proportions of cell populations. Bar height indicates the percentage of cells, and colors denote different cell subsets (right panel). (D, E) UMAP plot showing neutrophil subsets in liver, with colors representing distinct cell populations. Bar plot showing the relative proportions of neutrophil subsets. Bar height indicates the percentage of neutrophil subsets, and colors denote different neutrophil subsets (right panel). (F) Dot plot showing the top three marker genes for each neutrophil subset. Dot size indicates the percentage of cells expressing the gene, and color reflects average expression levels. (G) Heatmap displaying the top 10 marker genes for each neutrophil subset. Colors in the bar indicate subsets, while heatmap colors represent normalized gene expression levels. (H) Violin plot showing the maturation scores of neutrophil subsets. Embedded boxplots indicate the median values, with whiskers extending to the minimum and maximum non‐outlier values. (I) Violin plots showing reactive oxygen species (ROS) generation scores (left) and NADPH oxidase activity scores (right) across neutrophil subsets. Embedded boxplots indicate medians with whiskers denoting non‐outlier ranges. (J) Violin plot showing degranulation scores across neutrophil subsets, with embedded boxplots showing median values and whiskers denoting non‐outlier ranges. (K) Violin plot showing neutrophil extracellular trap (NET) formation scores across subsets. Embedded boxplots indicate medians with whiskers denoting non‐outlier ranges. (L) Violin plots showing reactive oxygen species (ROS) generation scores (left) and NADPH oxidase activity scores (right) across neutrophil subsets. Embedded boxplots indicate medians with whiskers denoting non‐outlier ranges. (M) Violin plot showing phagocytosis scores across neutrophil subsets, with embedded boxplots showing median values and whiskers denoting non‐outlier ranges. (N) Heatmap (left) showing the expression of bone marrow similarity–related genes in individual cells, and violin plot (right) showing the corresponding similarity scores across subsets. Embedded boxplots display median scores, with whiskers denoting non‐outlier ranges. (O) Dot plot showing the similarity scores of Xie's neutrophil signatures across different subsets. Both color and dot size represent mean similarity scores. (P) UMAP plot (left) showing aging score distribution across neutrophils. Violin plot (right) showing aging scores across subsets. Embedded boxplots indicate median values with whiskers denoting non‐outlier ranges.
**Figure S7:** Functional characterization of aging‐related neutrophils in lung tissues. (A) Bar plot showing the relative proportions of neutrophil subsets. Bar height indicates the percentage of neutrophil subsets, and colors denote different neutrophil subsets. (B) Dot plot showing the top three marker genes for each neutrophil subset. Dot size indicates the percentage of cells expressing the gene, and color reflects average expression levels. (C) Heatmap displaying the top 3 marker genes for each neutrophil subset. Colors in the bar indicate subsets, while heatmap colors represent normalized gene expression levels. (D) violin plot showing the corresponding BM similarity scores across subsets. Embedded boxplots display median scores, with whiskers denoting non‐outlier ranges. (E) Heatmap showing the expression of aging‐related clock genes across cells. Color scale represents expression intensity. (F) Violin plot (right) showing aging scores across neutrophil subsets. Embedded boxplots indicate median values with whiskers denoting non‐outlier ranges. (G) Dot plot showing the similarity scores of Xie's neutrophil signatures across different subsets. Both color and dot size represent mean similarity scores. (H) Violin plot showing degranulation scores across neutrophil subsets, with embedded boxplots showing median values and whiskers denoting non‐outlier ranges. (I) Violin plots showing NADPH oxidase activity scores across neutrophil subsets. Embedded boxplots indicate medians with whiskers denoting non‐outlier ranges. (J) Violin plot showing neutrophil extracellular trap (NET) formation scores across subsets. Embedded boxplots indicate medians with whiskers denoting non‐outlier ranges. (K) Violin plot showing phagocytosis scores across neutrophil subsets, with embedded boxplots showing median values and whiskers denoting non‐outlier ranges. (L) Violin plots showing reactive oxygen species (ROS) generation scores across neutrophil subsets. Embedded boxplots indicate medians with whiskers denoting non‐outlier ranges.
**Figure S8:** Functional characterization of aging‐related neutrophils in heart tissue. (A) Dot plot showing the top three marker genes for each neutrophil subset. Dot size indicates the percentage of cells expressing the gene, and color reflects average expression levels. (B) Bar plot showing the relative proportions of neutrophil subsets. Bar height indicates the percentage of neutrophil subsets, and colors denote different neutrophil subsets. (C) Heatmap displaying the top 3 marker genes for each neutrophil subset. Colors in the bar indicate subsets, while heatmap colors represent normalized gene expression levels. (D) violin plot showing the corresponding BM similarity scores across subsets. Embedded boxplots display median scores, with whiskers denoting non‐outlier ranges. (E) Heatmap showing the expression of aging‐related clock genes across cells. Color scale represents expression intensity. (F) Violin plot (right) showing aging scores across neutrophil subsets. Embedded boxplots indicate median values with whiskers denoting non‐outlier ranges. (G) Dot plot showing the similarity scores of Xie's neutrophil signatures across different subsets. Both color and dot size represent mean similarity scores. (H) Violin plot showing degranulation scores across neutrophil subsets, with embedded boxplots showing median values and whiskers denoting non‐outlier ranges. (I) Violin plots showing NADPH oxidase activity scores across neutrophil subsets. Embedded boxplots indicate medians with whiskers denoting non‐outlier ranges. (J) Violin plot showing neutrophil extracellular trap (NET) formation scores across subsets. Embedded boxplots indicate medians with whiskers denoting non‐outlier ranges. (K) Violin plot showing phagocytosis scores across neutrophil subsets, with embedded boxplots showing median values and whiskers denoting non‐outlier ranges. (L) Violin plots showing reactive oxygen species (ROS) generation scores across neutrophil subsets. Embedded boxplots indicate medians with whiskers denoting non‐outlier ranges.
**Figure S9:** Functional characterization of aging‐related neutrophils in blood. (A) Bar plot showing the relative proportions of neutrophil subsets. Bar height indicates the percentage of neutrophil subsets, and colors denote different neutrophil subsets. (B) Dot plot showing the top three marker genes for each neutrophil subset. Dot size indicates the percentage of cells expressing the gene, and color reflects average expression levels. (C) Heatmap displaying the top 3 marker genes for each neutrophil subset. Colors in the bar indicate subsets, while heatmap colors represent normalized gene expression levels. (D) violin plot showing the corresponding BM similarity scores across subsets. Embedded boxplots display median scores, with whiskers denoting non‐outlier ranges. (E) Heatmap showing the expression of aging‐related clock genes across cells. Color scale represents expression intensity. (F) Violin plot (right) showing aging scores across neutrophil subsets. Embedded boxplots indicate median values with whiskers denoting non‐outlier ranges. (G) Dot plot showing the similarity scores of Xie's neutrophil signatures across different subsets. Both color and dot size represent mean similarity scores. (H) Violin plot showing degranulation scores across neutrophil subsets, with embedded boxplots showing median values and whiskers denoting non‐outlier ranges. (I) Violin plots showing NADPH oxidase activity scores across neutrophil subsets. Embedded boxplots indicate medians with whiskers denoting non‐outlier ranges. (J) Violin plot showing neutrophil extracellular trap (NET) formation scores across subsets. Embedded boxplots indicate medians with whiskers denoting non‐outlier ranges. (K) Violin plot showing phagocytosis scores across neutrophil subsets, with embedded boxplots showing median values and whiskers denoting non‐outlier ranges. (L) Violin plots showing reactive oxygen species (ROS) generation scores across neutrophil subsets. Embedded boxplots indicate medians with whiskers denoting non‐outlier ranges.
**Figure S10:** Functional characterization of aging‐related neutrophils in intestine. (A) Bar plot showing the relative proportions of neutrophil subsets. Bar height indicates the percentage of neutrophil subsets, and colors denote different neutrophil subsets. (B) Dot plot showing the top three marker genes for each neutrophil subset. Dot size indicates the percentage of cells expressing the gene, and color reflects average expression levels. (C) Heatmap displaying the top 3 marker genes for each neutrophil subset. Colors in the bar indicate subsets, while heatmap colors represent normalized gene expression levels. (D) violin plot showing the corresponding BM similarity scores across subsets. Embedded boxplots display median scores, with whiskers denoting non‐outlier ranges. (E) Heatmap showing the expression of aging‐related clock genes across cells. Color scale represents expression intensity. (F) Violin plot (right) showing aging scores across neutrophil subsets. Embedded boxplots indicate median values with whiskers denoting non‐outlier ranges. (G) Dot plot showing the similarity scores of Xie's neutrophil signatures across different subsets. Both color and dot size represent mean similarity scores. (H) Violin plot showing degranulation scores across neutrophil subsets, with embedded boxplots showing median values and whiskers denoting non‐outlier ranges. (I) Violin plots showing NADPH oxidase activity scores across neutrophil subsets. Embedded boxplots indicate medians with whiskers denoting non‐outlier ranges. (J) Violin plot showing neutrophil extracellular trap (NET) formation scores across subsets. Embedded boxplots indicate medians with whiskers denoting non‐outlier ranges. (K) Violin plot showing phagocytosis scores across neutrophil subsets, with embedded boxplots showing median values and whiskers denoting non‐outlier ranges. (L) Violin plots showing reactive oxygen species (ROS) generation scores across neutrophil subsets. Embedded boxplots indicate medians with whiskers denoting non‐outlier ranges.
**Figure S11:** Functional characterization of aging‐related neutrophils in kidney. (A) Bar plot showing the relative proportions of neutrophil subsets. Bar height indicates the percentage of neutrophil subsets, and colors denote different neutrophil subsets. (B) Dot plot showing the top three marker genes for each neutrophil subset. Dot size indicates the percentage of cells expressing the gene, and color reflects average expression levels. (C) Heatmap displaying the top 3 marker genes for each neutrophil subset. Colors in the bar indicate subsets, while heatmap colors represent normalized gene expression levels. (D) violin plot showing the corresponding BM similarity scores across subsets. Embedded boxplots display median scores, with whiskers denoting non‐outlier ranges. (E) Heatmap showing the expression of aging‐related clock genes across cells. Color scale represents expression intensity. (F) Violin plot (right) showing aging scores across neutrophil subsets. Embedded boxplots indicate median values with whiskers denoting non‐outlier ranges. (G) Dot plot showing the similarity scores of Xie's neutrophil signatures across different subsets. Both color and dot size represent mean similarity scores. (H) Violin plot showing degranulation scores across neutrophil subsets, with embedded boxplots showing median values and whiskers denoting non‐outlier ranges. (I) Violin plots showing NADPH oxidase activity scores across neutrophil subsets. Embedded boxplots indicate medians with whiskers denoting non‐outlier ranges. (J) Violin plot showing neutrophil extracellular trap (NET) formation scores across subsets. Embedded boxplots indicate medians with whiskers denoting non‐outlier ranges. (K) Violin plot showing phagocytosis scores across neutrophil subsets, with embedded boxplots showing median values and whiskers denoting non‐outlier ranges. (L) Violin plots showing reactive oxygen species (ROS) generation scores across neutrophil subsets. Embedded boxplots indicate medians with whiskers denoting non‐outlier ranges.
**Figure S12:** Tissue‐specific enrichment of aging‐associated neutrophil programs and neutrophil states across organs. (A) Heatmap showing the relative enrichment of neutrophil functional signatures across tissues. Functional programs, including reactive oxygen species (ROS) production, degranulation, NETosis, complement activation, interferon (IFN) response, IL‐6/STAT3 signaling, inflammation, and cellular aging/senescence, were scored in neutrophils from each tissue and averaged at the tissue level. Colors represent scaled signature scores (Z‐scores), with red indicating higher enrichment and blue indicating lower enrichment. Hierarchical clustering was performed on both tissues and functional signatures. (B) Dot plot illustrating age‐associated changes in neutrophil functional programs across tissues. Dot color represents the difference in signature scores between aged and young mice (Old − Young), whereas dot size indicates the absolute magnitude of the change. Positive values (red) denote increased pathway activity in aged neutrophils, and negative values (purple) indicate reduced activity. Notably, NETosis, IL‐6/STAT3 signaling, inflammatory responses, and ROS‐related programs were preferentially increased in aged neutrophils across multiple tissues. (C) Identification of cross‐tissue neutrophil functional meta‐programs. Neutrophil subclusters from individual tissues were grouped according to their functional signature profiles, including maturation, aging score, NETosis, phagocytosis, bone marrow proximity, degranulation, ROS production, and NADPH oxidase activity. Each row represents a tissue‐specific neutrophil subtype, and columns represent functional programs. Values are shown as scaled signature scores (Z‐scores). Hierarchical clustering revealed conserved functional meta‐programs shared across tissues, highlighting common neutrophil activation states characterized by inflammatory, oxidative, and maturation‐associated signatures.
**Figure S13:** Human single‐cell atlas analysis validates conserved aging‐associated neutrophil states across tissues. (A) UMAP visualization of integrated human neutrophils following Harmony‐based batch correction and graph‐based clustering. Fourteen transcriptionally distinct neutrophil states were identified and numbered according to cluster identity. (B) Hallmark pathway enrichment analysis of cluster‐specific marker genes. Dot size represents the number of enriched genes within each pathway, and color intensity indicates enrichment significance (−log10 adjusted *p* value). Distinct neutrophil states were characterized by pathways involved in inflammatory signaling, oxidative stress, complement activation, the unfolded protein response, cell‐cycle regulation, interferon signaling, and metabolic remodeling. (C) Functional annotation of human neutrophil states based on dominant enriched pathways. Identified states included IL6‐associated neutrophils (IL6–Neu01), TNFA‐associated neutrophils (TNFA–Neu02), PI3K‐signaling neutrophils (PI3K–Neu03), secretory neutrophils (Secretion–Neu04), inflammatory neutrophils (Inflammation–Neu05), MYC‐associated neutrophils (MYC–Neu06), MTOR‐associated neutrophils (MTOR–Neu07), apoptosis‐associated neutrophils (Apoptosis–Neu08), oxidative stress‐associated neutrophils (Oxidative–Neu09), interferon‐responsive neutrophils (IFN–Neu10), complement‐associated neutrophils (Complement–Neu11), Neu12, unfolded protein response‐associated neutrophils (UPR–Neu13), and E2F‐associated neutrophils (E2F–Neu14). (D) Age‐dependent distribution of human neutrophil states. Each point represents the percentage of a given neutrophil state within an individual donor. Several aging‐associated neutrophil populations, including Complement–Neu11, Oxidative–Neu09, IL6–Neu01, and UPR–Neu13, showed increased abundance with advancing age, whereas other states remained relatively stable.
**Figure S14:** Leave‐one‐tissue‐out analysis demonstrates the robustness of neutrophil transcriptional programs across organs. (A–H) Pairwise correlation matrices of aging‐associated neutrophil transcriptional programs generated using a leave‐one‐tissue‐out strategy. For each analysis, neutrophils from one tissue were excluded before recalculating gene–gene correlation networks: brain (A), kidney (B), intestine (C), blood (D), lung (E), heart (F), liver (G), and vascular tissue (H). Genes are ordered by hierarchical clustering based on the correlation structure. Red indicates positive correlations, whereas blue indicates negative correlations. (I) Tissue‐dependency scores for each full‐data co‐abundance module (CM) following sequential removal of individual tissues. The tissue‐dependency score represents the reduction in CM recurrence associated with removal of the indicated tissue relative to the other LOTO iterations; larger positive values indicate greater dependence of a CM on that tissue. (J) Recurrence of full‐data CMs across LOTO iterations. For each iteration, one tissue was excluded, CMs were independently reconstructed using the same clustering procedure, and each full‐data CM was matched to the LOTO‐derived module with the greatest membership overlap. Values indicate the Jaccard similarity between the full‐data CM and its best‐matched LOTO module.
**Figure S15:** The cell–cell communication networks across different neutrophils. (A) The dot plot showing different cell component expression across different tissues. The size of the dot represent cell count in tissues. The color of the dot represents the gene expression in each tissue. (B) The violin plot of the chemokine expression in different cell populations and neutrophil subsets. Color representing the old and young groups. (C–I) The chordal plot of different cell populations across different groups in blood (C), heart (D), intestine (E), kidney (F), liver (G), lung (H), and vascular (I) tissues. Color representing the different cell types. The width of the edges shows the strength of the communication among cells.
**Figure S16:** The communication of chemokines between different cell types in tissues. (A–F) the dot plot showing cell–cell communication strength of chemokines in vascular (A), liver (B), heart (C), lung (D), blood (E), and intestine (F). The color represents chemokine strength between two cell types.
**Figure S17:** IL‐6 stimulation induces MOVAS and AML‐12 cell senescence‐associated response. (A) β‐Gal staining of MOVAS and AML‐12 cells after treatment with IL‐6 at indicated dose. Bar = 20 μm. (B, C) RNA levels of SASP and ECM‐related genes (*Il1*, *Tnf*, *Ifn*, *Il17*, *Mmp3*, *Mmp9*, and *Mmp12*) in MOVAS following treatment with IL‐6. IL‐6^high^ = 10 ng/mL, IL‐6^low^ = 1 ng/mL. (D, E) Protein expression of indicated markers in MOVAS and AML‐12 cells after treatment with IL‐6 at the indicated dose. (F) RNA levels of SASP and ECM‐related genes (*Il6*, *Il1a*, *Il1b*, *Tnf*, *Ifn*, *Mmp3*, and *Mmp12*) in MOVAS following treatment with conditioned medium from HL‐60 after stimulation with IL‐6 at the indicated dose. Data are shown as the mean ± s.e.m., *p < 0.05; **p < 0.01; ***p < 0.001. *t*‐test was used in (A) *Two‐way ANOVA* followed by Tukey's multiple comparisons test was used in (B–F).
**Figure S18:** The important aging‐related pathway alterations in neutrophil subtypes. (A) The violin plot showing p53 (left), TGFβ (middle), and TNFα (right) alterations in Neu06‐IL6 from young and aged neutrophils, with embedded boxplots showing median values and whiskers denoting non‐outlier ranges. (B) The dot plot showing important pathway alterations in different neutrophil subtypes. The size indicates the percentage of pathway‐positive cells. The color represents the pathway average expression of neutrophil subtypes. (C) The violin plot showing BMP signaling expression in Neu04‐MYC and Neu06‐IL6, with embedded boxplots showing median values and whiskers denoting non‐outlier ranges. (D) The violin plot showing TGFβ signaling expression in Neu03‐Allograft and Neu08‐E2F, with embedded boxplots showing median values and whiskers denoting non‐outlier ranges. (E) The dot plot showing different inflammation pathway expression in different neutrophil subtypes. The size represents the expression of the inflammatory pathway, and the color shows the average expression across different neutrophil subtypes. (F) The violin plot showing integration of energy metabolism in Neu02‐inflammatory and Neu13‐TGFB, and Neu16‐TNFA, with embedded boxplots showing median values and whiskers denoting non‐outlier ranges. (G) The violin plot showing fatty acid metabolism in Neu02‐inflammatory and Neu3‐Allograft, and Neu04‐MYC, with embedded boxplots showing median values and whiskers denoting non‐outlier ranges. (H) The violin plot showing cellular response to fatty acid in Neu02‐inflammatory and Neu08‐E2F, with embedded boxplots showing median values and whiskers denoting non‐outlier ranges. (I) The violin plot showing IGF1R pathway in Neu04‐MYC, with embedded boxplots showing median values and whiskers denoting non‐outlier ranges. (J) The violin plot showing oxidative phosphorylation score in Neu02‐inflammatory, Neu3‐Allograft and Neu04‐MYC, with embedded boxplots showing median values and whiskers denoting non‐outlier ranges. (K) The violin plot showing CX3C chemokine binding score in Neu04‐MYC, Neu06‐IL6, and Neu16‐TNFA, with embedded boxplots showing median values and whiskers denoting non‐outlier ranges. (L) The violin plot showing integrated stress response signaling score in Neu02‐inflammatory, Neu04‐MYC, and Neu08‐E2F, with embedded boxplots showing median values and whiskers denoting non‐outlier ranges. (M) The violin plot showing chemokine signaling score in Neu01‐TNFA, Neu02‐inflammatory, Neu04‐MYC, Neu06‐IL6, Neu07‐IFNA, Neu13‐TGFB, Neu10‐Xenobiotic, and Neu12‐UPR, with embedded boxplots showing median values and whiskers denoting non‐outlier ranges.
**Figure S19:** Bone marrow single‐cell landscape of young and aged mice. (A) UMAP plot showing cell population distribution across different groups. Bar plot showing the cell populations in young and aged mice. Color represents the cell populations in a mouse's bone marrow. (B) UMAP plot showing hematopoietic precursor cells, granulocytopoiesis cells, and granulocytes in bone marrow of a mouse (left panel). Bar plot showing the relative proportions of cell populations. Bar height indicates the percentage of cells, and colors denote different cell subsets (right panel). (C) Dot plot showing the similarity scores of Xie's neutrophil signatures across different subsets in HPC, granulocyte progenitor, and granulocyte. Both color and dot size represent mean similarity scores. (D) The pie chart showing the cell percentages across different neutrophil stages in young and aged mice, with colors representing the different bone marrow cell types. (E) The landscape of neutrophil development in bone marrow.

**Table S1:** Biological function annotation of neutrophil subtypes based on Hallmark gene set enrichment. Over‐representation analysis was performed using the Hallmark database to identify subtype‐specific marker gene–associated pathways. *p* Values were calculated using Fisher's exact test, and adjusted *p* values were obtained using the Benjamini–Hochberg (BH) multiple‐testing correction.

**Table S2:** Gene Ontology Biological Process (GO BP) enrichment analysis of HPC5. Over‐representation analysis of GO BP terms was conducted to characterize the biological functions associated with HPC5‐targeted genes. *p* Values were calculated using Fisher's exact test, and adjusted *p* values were obtained using the Benjamini–Hochberg (BH) method.

## Data Availability

The data that support the findings of this study are available on request from the corresponding author. The data are not publicly available due to privacy or ethical restrictions.
