# Supplementary material for: Single‐Cell Mapping Identifies Aging‐Associated Neutrophil Remodeling Across Multiple Organs
Source: Aging Cell. 2026 Sep 29;25(10):e70735. doi: 10.1111/acel.70735 (PMC13624379; doi:10.1111/acel.70735)
Supplement: Supplementary file 1 [file ACEL-25-e70735-s003.pdf]

Supplementary Figures:

Single-cell mapping identifies aging-associated neutrophil remodeling across multiple organs

Yuman Wang[1,2#], Teding Chang[3,4#], Yangguang Fu[2,5], Guangyu Liang[2,5], Huicheng Liu[6], Le Zhang[1,2], Jinhua Yan[1,2], Man Liu[1,2*], Anding Liu[2,5*], Cuntai Zhang[1,2*]

[1]Department of Geriatrics, Tongji Hospital, Tongji Medical College, Huazhong University of Science and Technology, 1095 Jiefang Avenue, Wuhan 430030, China.

[2] Key Laboratory of Vascular Aging, Ministry of Education, Tongji Hospital, Tongji Medical College, Huazhong University of Science and Technology, 1095 Jiefang Avenue, Wuhan 430030, China

[3]Department of Traumatic Surgery, Tongji Trauma Center, Tongji Hospital, Tongji Medical College, Huazhong University of Science and Technology, 1095 Jiefang Avenue, Wuhan 430030, China

[4]Department of Surgery, University of Pittsburgh, Pittsburgh, PA, USA

[5]Experimental Medicine Center, Tongji Hospital, Tongji Medical College, Huazhong University of Science and Technology, Wuhan, China.

[6]Department of Otolaryngology-Head and Neck Surgery, Tongji Hospital, Tongji Medical College, Huazhong University of Science and Technology

[#]These authors contributed equally.

***Corresponding author:**

Cuntai Zhang: ctzhang@tjh.tjmu.edu.cn

Anding Liu: andingliu@tjh.tjmu.edu.cn

Man Liu: manliu@hust.edu.cn

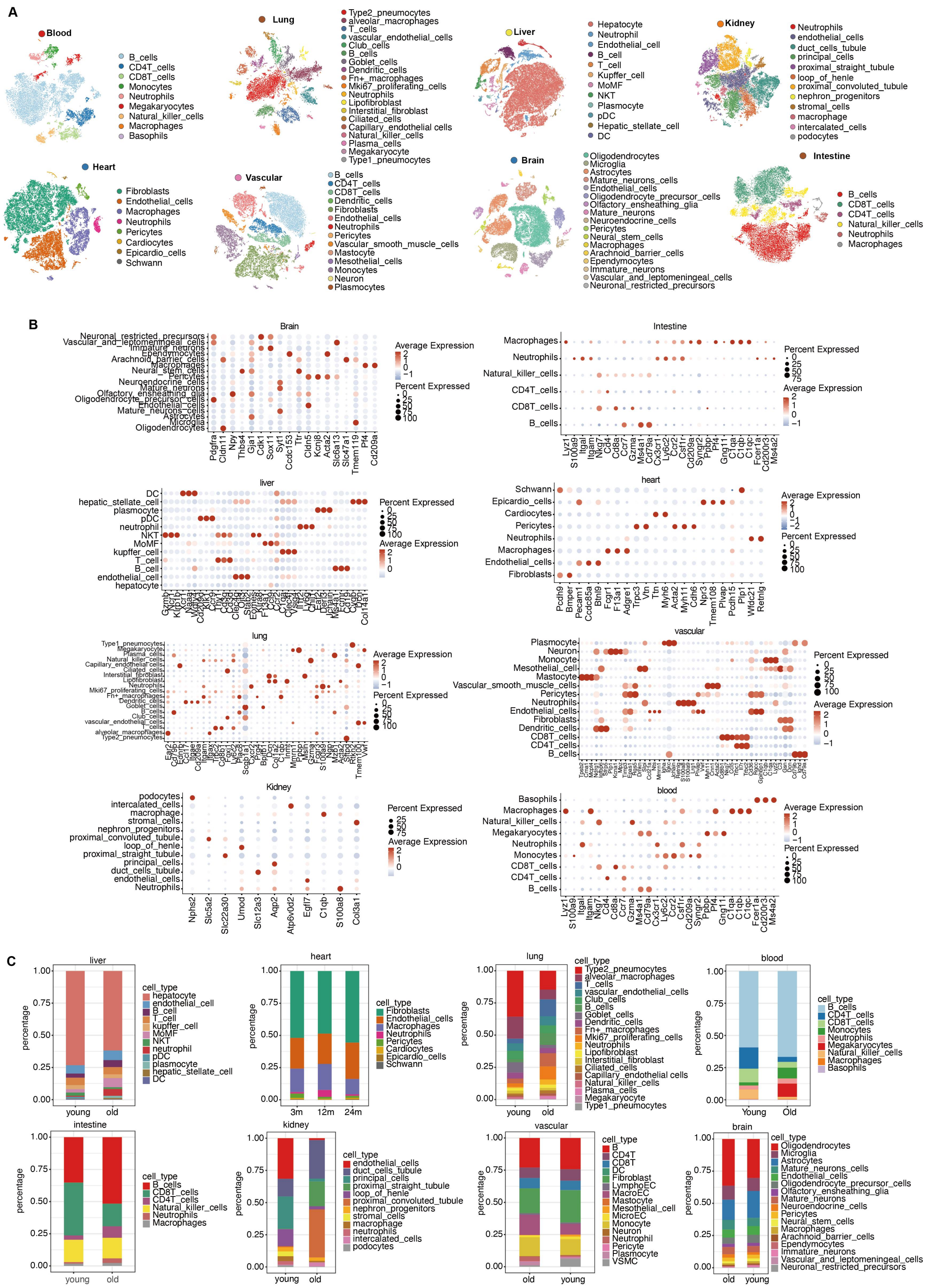

Supplementary Figure 1. Neutrophil infiltration is increased in multiple organs of aged mice. **A**, t-SNE plot displaying the distribution of single-cell transcriptomes across various tissues in young and aged mice. **B**, Dot plot showing the expression of canonical marker genes used for cell type annotation across tissues. Dot color indicates the average expression level of each marker gene, and dot size represents the percentage of cells expressing the marker within each cluster. **C**, Stacked bar plot illustrating the relative proportions of major cell types across tissues in young versus aged mice. Bar segments are colored by cell type, and the size of each segment reflects the percentage of that cell type within the tissue.

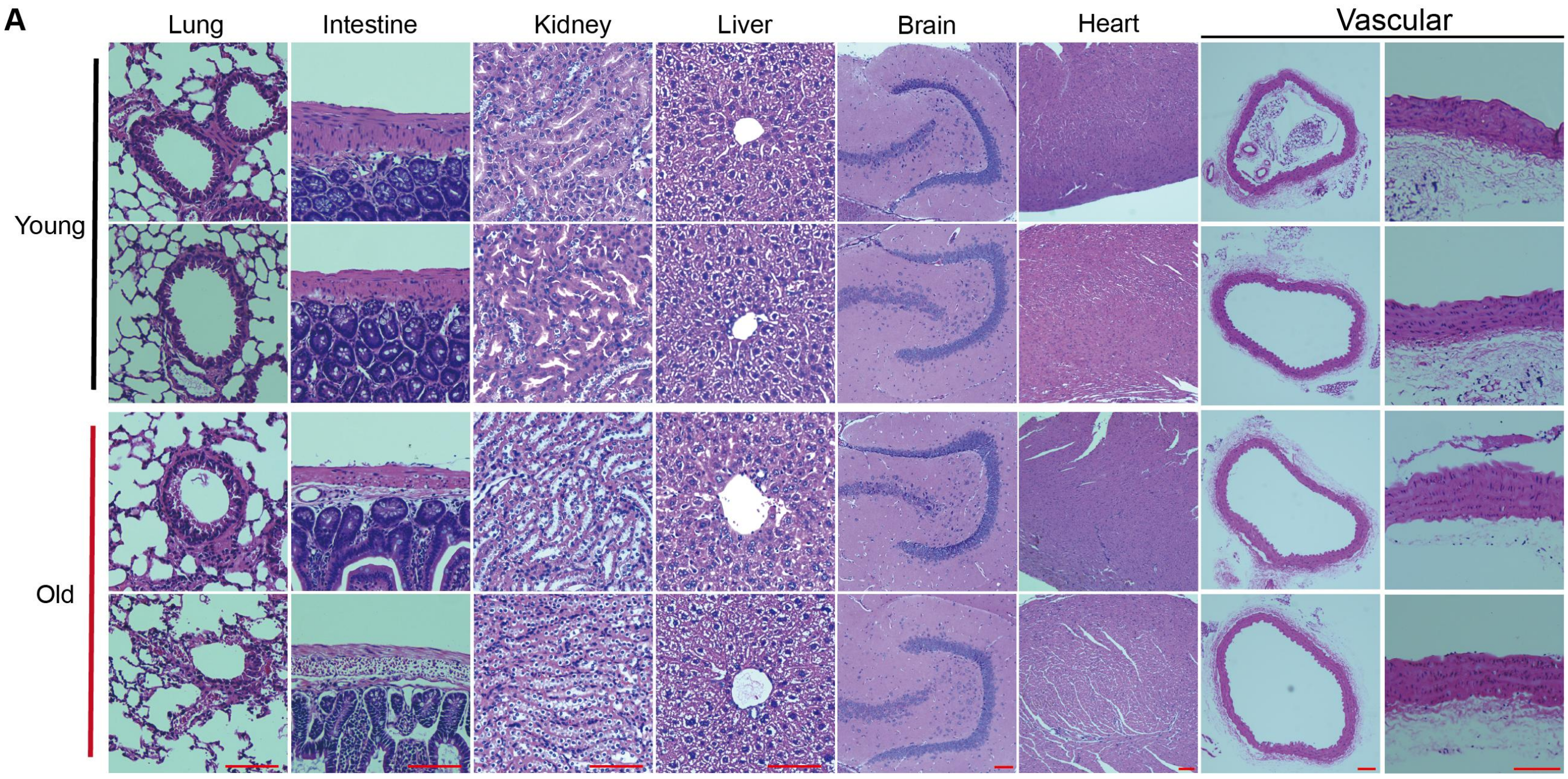

Supplementary Figure 2. Morphology of tissues from young and old mice. A, HE stains of multiple organs (lung, intestine, kidney, liver, brain, and heart) from young and old mice. Bar = 20 mm.

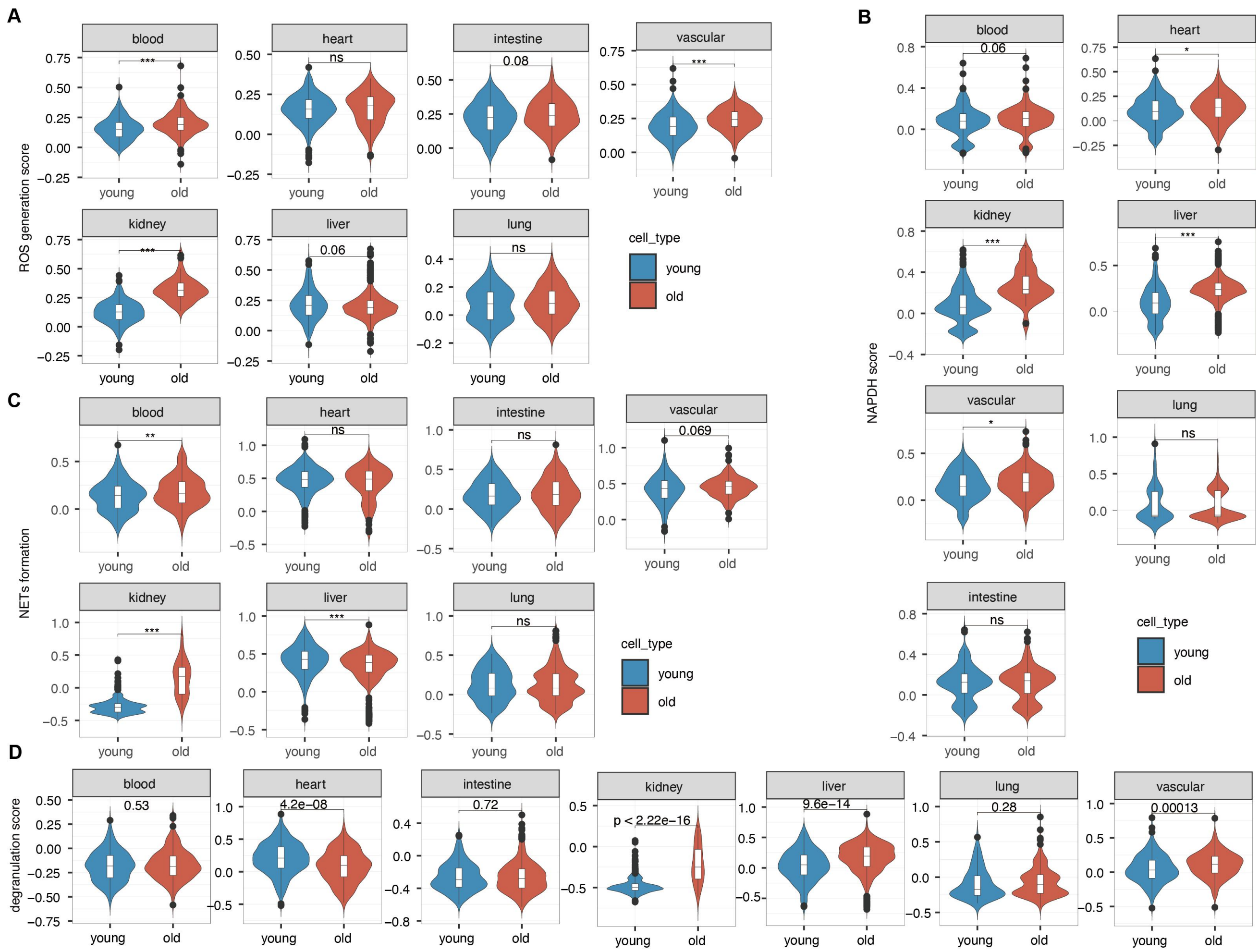

Supplementary Figure 3. Aging-associated neutrophils (AANs) exhibit enhanced pro- inflammatory activity across multiple tissues. A-D, Violin plots showing module scores for ROS generation (**A**), glycolytic activity (NADPH score, **B**), NETs formation (**C**), and degranulation (**D**) in neutrophils from different tissues in young versus aged mice. The shape of each violin reflects the distribution density of scores; embedded box plots display the interquartile range (IQR) and median. Statistical significance was assessed using the Wilcoxon rank-sum test. ns, not significant.

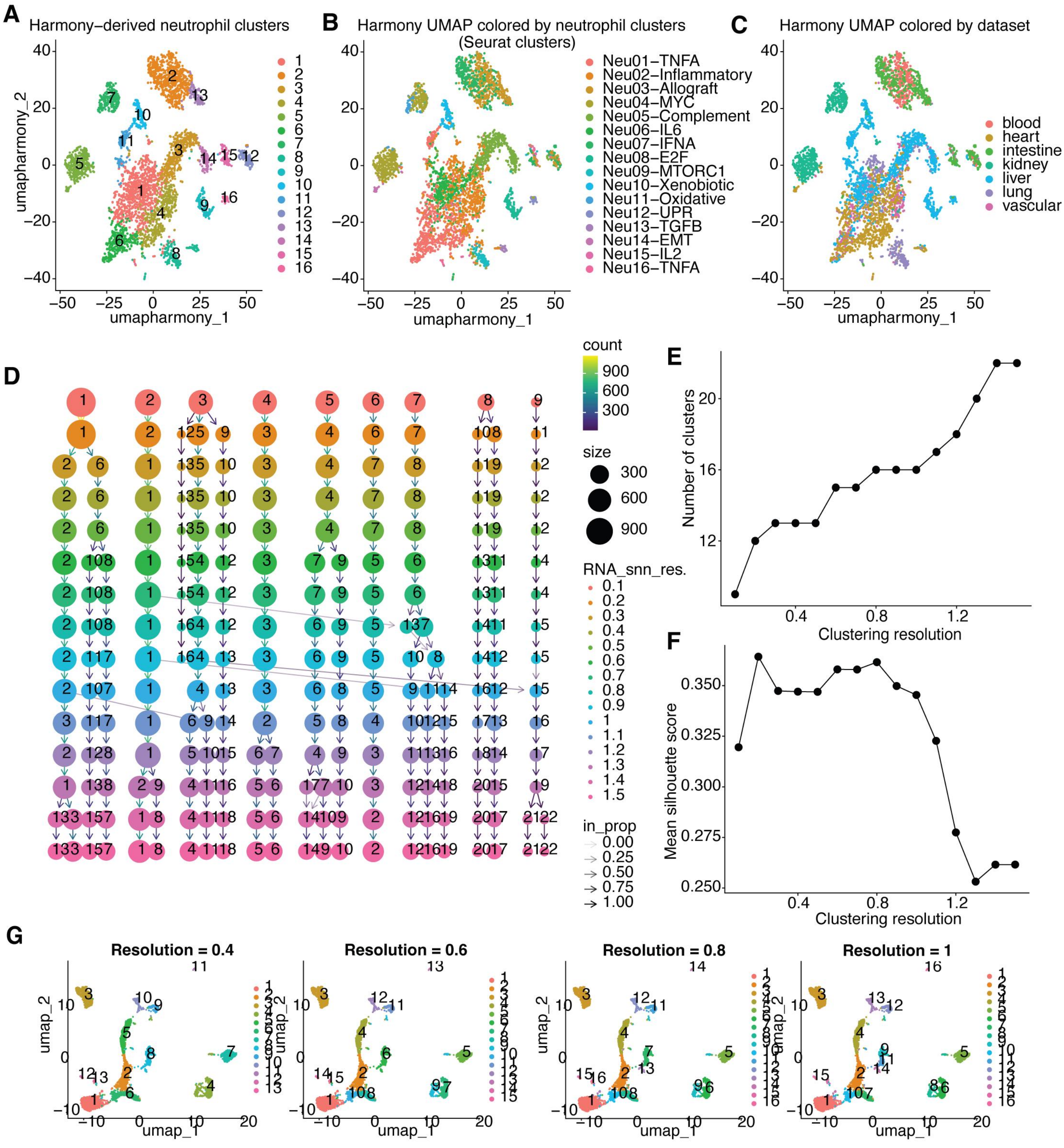

Supplementary Figure 4. Resolution optimization and clustering stability analysis identify age-associated neutrophil subpopulations. A. Uniform Manifold Approximation and Projection (UMAP) visualization of Harmony-integrated neutrophils from multiple tissues. Graph-based clustering identified 16 neutrophil populations (Neu01–Neu16), which are colored according to cluster identity. Cluster numbers are overlaid on the corresponding cellular populations. Batch correction and data integration were performed using Harmony to minimize tissue-specific efifects while preserving biological variation. **B.** UMAP visualization of the same Harmony-integrated neutrophils colored by datasets, including blood, heart, intestine, kidney, liver, lung, and vascular tissues. Cells from different datasets were broadly intermingled within clusters, indicating successful integration and shared neutrophil transcriptional programs across organs. **C.** UMAP visualization colored by annotated neutrophil states. Cluster identities were assigned

based on the dominant transcriptional programs identified by differential gene expression and pathway enrichment analyses. Annotated neutrophil states included TNFA signaling (Neu01 and Neu16), inflammatory neutrophils (Neu02), allograft rejection–associated neutrophils (Neu03), MYC signaling (Neu04), complement-associated neutrophils (Neu05), IL-6 signaling (Neu06), IFNA response (Neu07), E2F target programs (Neu08), MTORC1 signaling (Neu09), xenobiotic metabolism (Neu10), oxidative phosphorylation (Neu11), unfolded protein response (UPR; Neu12), TGF-β signaling (Neu13), epithelial–mesenchymal transition (EMT; Neu14), IL-2 signaling (Neu15), and TNFA signaling (Neu16). Data were integrated using Harmony following standard single-cell RNA-sequencing preprocessing, dimensionality reduction, and graph-based clustering. Cell-state annotations were assigned based on the dominant enriched Hallmark pathways within each cluster. **D.** Clustree visualization illustrating cluster transitions across a range of graph-based clustering resolutions (0.1–1.5) following dimensionality reduction and neighborhood graph construction. Each node represents a cluster at a given resolution, with node size proportional to the number of cells and color indicating cluster abundance. Edges connect cells shared between clusters across adjacent resolutions, enabling visualization of cluster stability and subdivision as resolution increases. **E.** Relationship between clustering resolution and the total number of identified neutrophil clusters. Increasing resolution led to a progressive subdivision of cellular populations, with the number of clusters rising from 11 at low resolution to 22 at the highest resolution tested. **F.** Mean silhouette score across clustering resolutions, used to evaluate cluster compactness and separation. The highest silhouette scores were observed at intermediate resolutions, indicating improved cluster stability and biological coherence compared with higher-resolution over-partitioning. **G.** Uniform manifold approximation and projection (UMAP) visualization of neutrophils clustered at representative resolutions (0.4, 0.6, 0.8, and 1.0). Cells are colored by cluster identity and labeled with their cluster numbers. Increasing clustering resolution progressively resolved additional neutrophil subpopulations while preserving the overall manifold structure. Based on the combined assessment of cluster stability, silhouette score, and biological interpretability, the selected resolution was used for downstream analyses. Data were analyzed using graph-based community detection following single-cell RNA sequencing preprocessing, dimensionality reduction, and nearest-neighbor graph construction. Cluster robustness was assessed by clustree analysis and silhouette scores across multiple clustering resolutions.

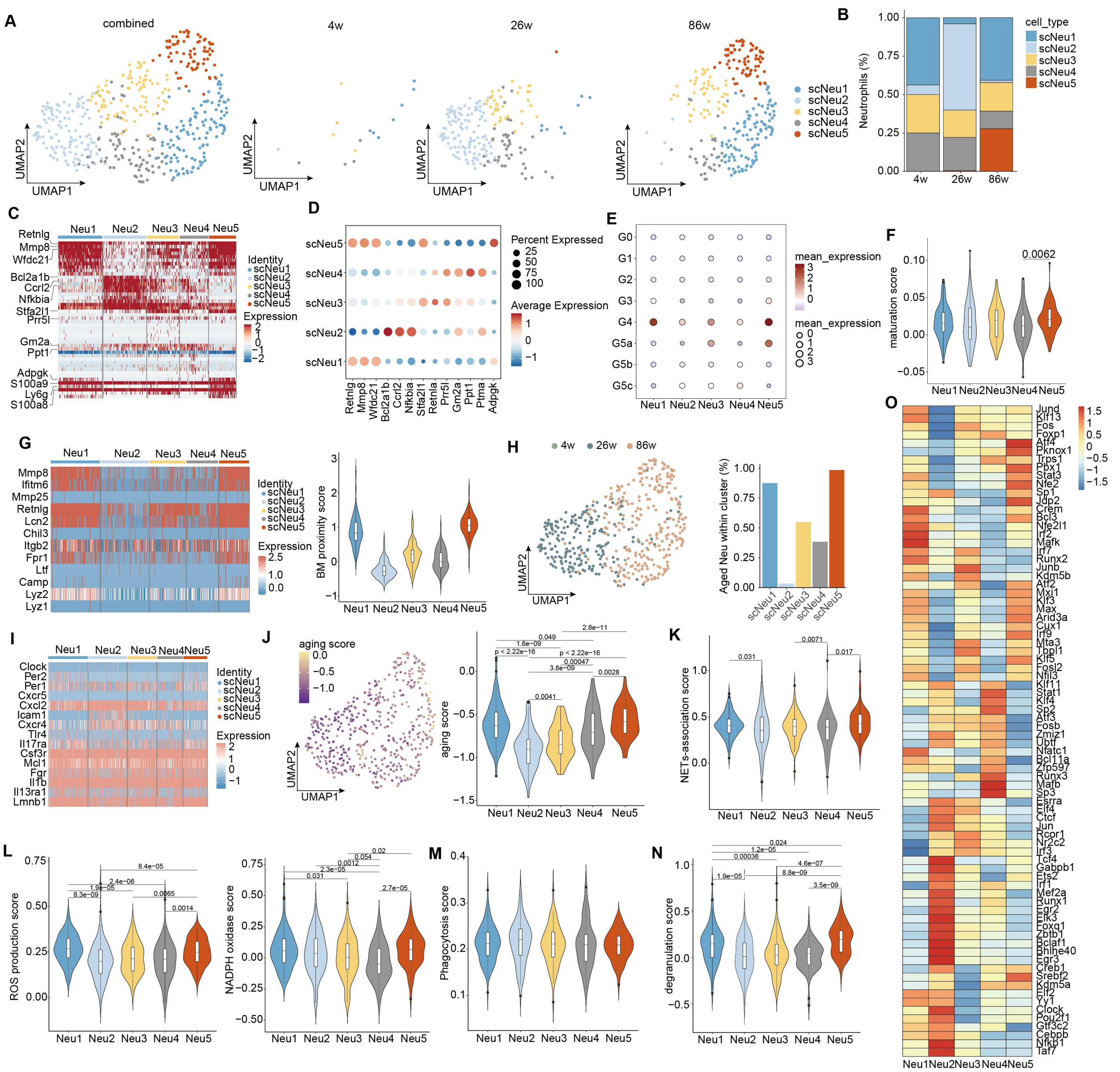

Supplementary Figure 5. Functional characterization of aging-related neutrophils in vascular tissue. **A**, UMAP plot showing neutrophil subsets in vascular tissue, with colors representing distinct neutrophil populations. **B**, Bar plot showing the relative proportions of neutrophil subsets. Bar height indicates the percentage of cells, and colors denote different subsets. **C**, Heatmap displaying the top 10 marker genes for each neutrophil subset. Colors in the bar indicate subsets, while heatmap colors represent normalized gene expression levels. **D**, Dot plot showing the top three marker genes for each neutrophil subset. Dot size indicates the percentage of cells expressing the gene, and color reflects average expression levels. **E**, Dot plot showing the similarity scores of Xie's neutrophil signatures across different subsets. Both color and dot size represent mean similarity scores. **F**, Violin plot showing the maturation scores of neutrophil subsets. Embedded boxplots indicate the median values, with whiskers extending to the minimum and maximum non-outlier values. **G**, Heatmap (left) showing the expression of bone marrow similarity–related genes in individual cells, and violin plot (right) showing the corresponding similarity scores across subsets. Embedded boxplots display median scores, with whiskers denoting non-outlier ranges. **H**, UMAP plot (left) showing the distribution of aging-related

neutrophils, with colors representing cell origins from mice of different ages. Bar plot (right) showing the proportion of aging-related neutrophils in each subset. **I**, Heatmap showing the expression of aging-related clock genes across cells. Color scale represents expression intensity. **J**, UMAP plot (left) showing aging score distribution across neutrophils. Violin plot (right) showing aging scores across subsets. Embedded boxplots indicate median values with whiskers denoting non-outlier ranges. **K**, Violin plot showing neutrophil extracellular trap (NET) formation scores across subsets. Embedded boxplots indicate medians with whiskers denoting non-outlier ranges. **L**, Violin plot showing reactive oxygen species (ROS) generation scores (left) and NADPH oxidase activity scores (right) across neutrophil subsets. Embedded boxplots indicate medians with whiskers denoting non-outlier ranges. **M**, Violin plot showing phagocytosis scores across neutrophil subsets, with embedded boxplots showing median values and whiskers denoting non-outlier ranges. **N**, Violin plot showing degranulation scores across neutrophil subsets, with embedded boxplots showing median values and whiskers denoting non-outlier ranges. **O**, Heatmap showing transcription factor activity across neutrophil subsets as determined by SCENIC analysis. Color represents relative transcription factor activation levels.

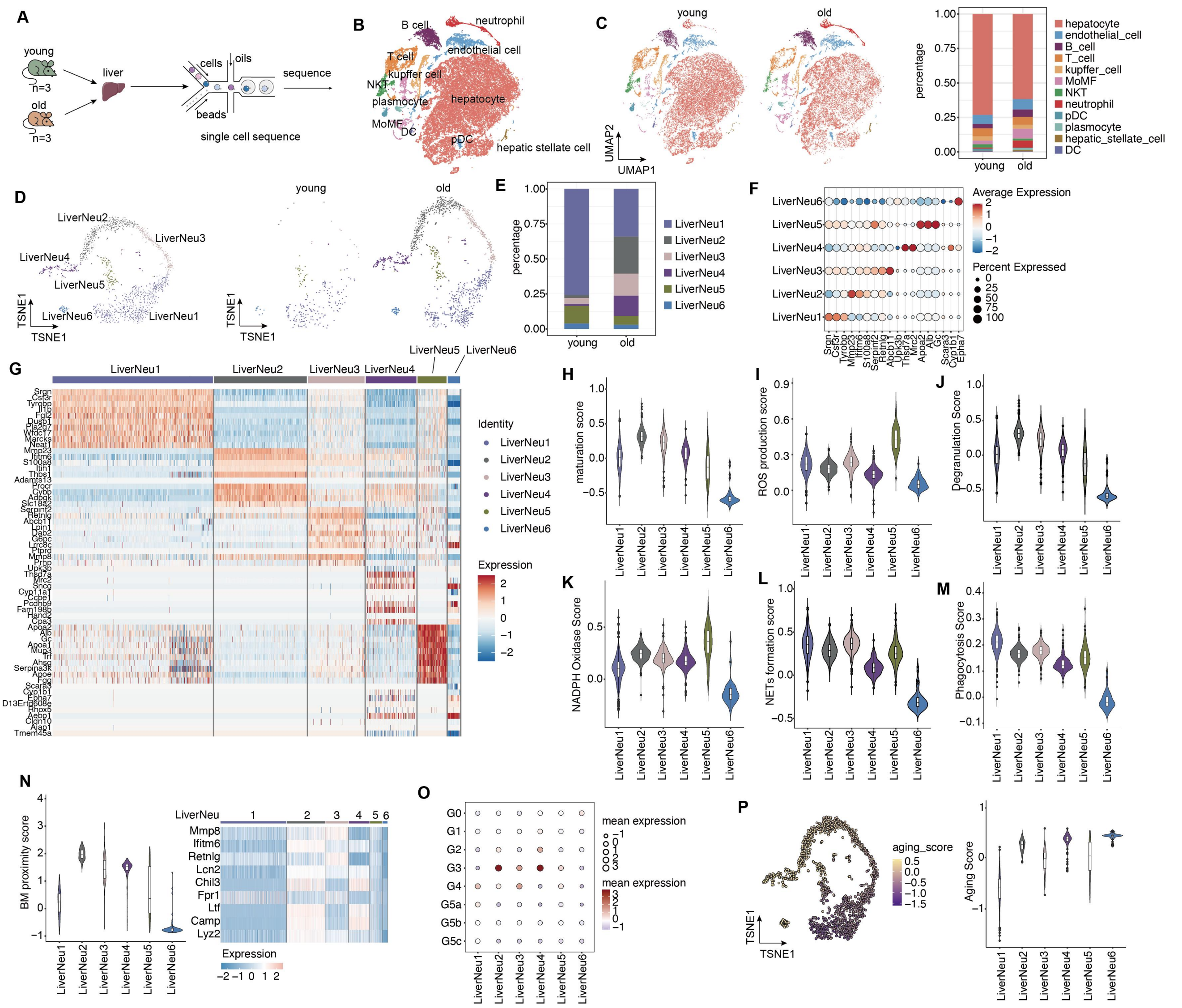

Supplementary Figure 6. Functional characterization of aging-related neutrophils in liver tissues. A, The overall design of the mouse single-cell liver analysis. **B**, UMAP plot showing cell populations in liver, with colors representing distinct cell populations. **C**, UMAP plot showing neutrophil subsets in liver of different ages, with colors representing distinct cell populations (left panel). Bar plot showing the relative proportions of cell populations. Bar height indicates the percentage of cells, and colors denote different cell subsets (right panel). **D-E**, UMAP plot showing neutrophil subsets in liver, with colors representing distinct cell populations. Bar plot showing the relative proportions of neutrophil subsets. Bar height indicates the percentage of neutrophil subsets, and colors denote different neutrophil subsets (right panel). **F**, Dot plot showing the top three marker genes for each neutrophil subset. Dot size indicates the percentage of cells expressing the gene, and color reflects average expression levels. **G**, Heatmap displaying the top 10 marker genes for each neutrophil subset. Colors in the bar indicate subsets, while heatmap colors represent normalized gene expression levels. **H**, Violin plot showing the maturation scores of neutrophil subsets. Embedded boxplots indicate the median values, with whiskers extending to the minimum and maximum non-outlier values. **I**, Violin plots showing reactive oxygen species (ROS) generation scores (left) and NADPH oxidase activity scores (right) across neutrophil subsets. Embedded boxplots indicate medians with whiskers denoting non-outlier ranges. **J**, Violin plot showing degranulation scores across neutrophil subsets, with embedded boxplots showing median values and whiskers denoting non-outlier ranges. **K**, Violin plot showing neutrophil extracellular trap (NET) formation scores across subsets. Embedded boxplots indicate medians with whiskers denoting non-outlier ranges. **L**, Violin plots showing reactive oxygen species (ROS)

generation scores (left) and NADPH oxidase activity scores (right) across neutrophil subsets. Embedded boxplots indicate medians with whiskers denoting non-outlier ranges. **M**, Violin plot showing phagocytosis scores across neutrophil subsets, with embedded boxplots showing median values and whiskers denoting non-outlier ranges. **N**, Heatmap (left) showing the expression of bone marrow similarity–related genes in individual cells, and violin plot (right) showing the corresponding similarity scores across subsets. Embedded boxplots display median scores, with whiskers denoting non-outlier ranges. **O**, Dot plot showing the similarity scores of Xie's neutrophil signatures across different subsets. Both color and dot size represent mean similarity scores. **P**, UMAP plot (left) showing aging score distribution across neutrophils. Violin plot (right) showing aging scores across subsets. Embedded boxplots indicate median values with whiskers denoting non-outlier ranges.

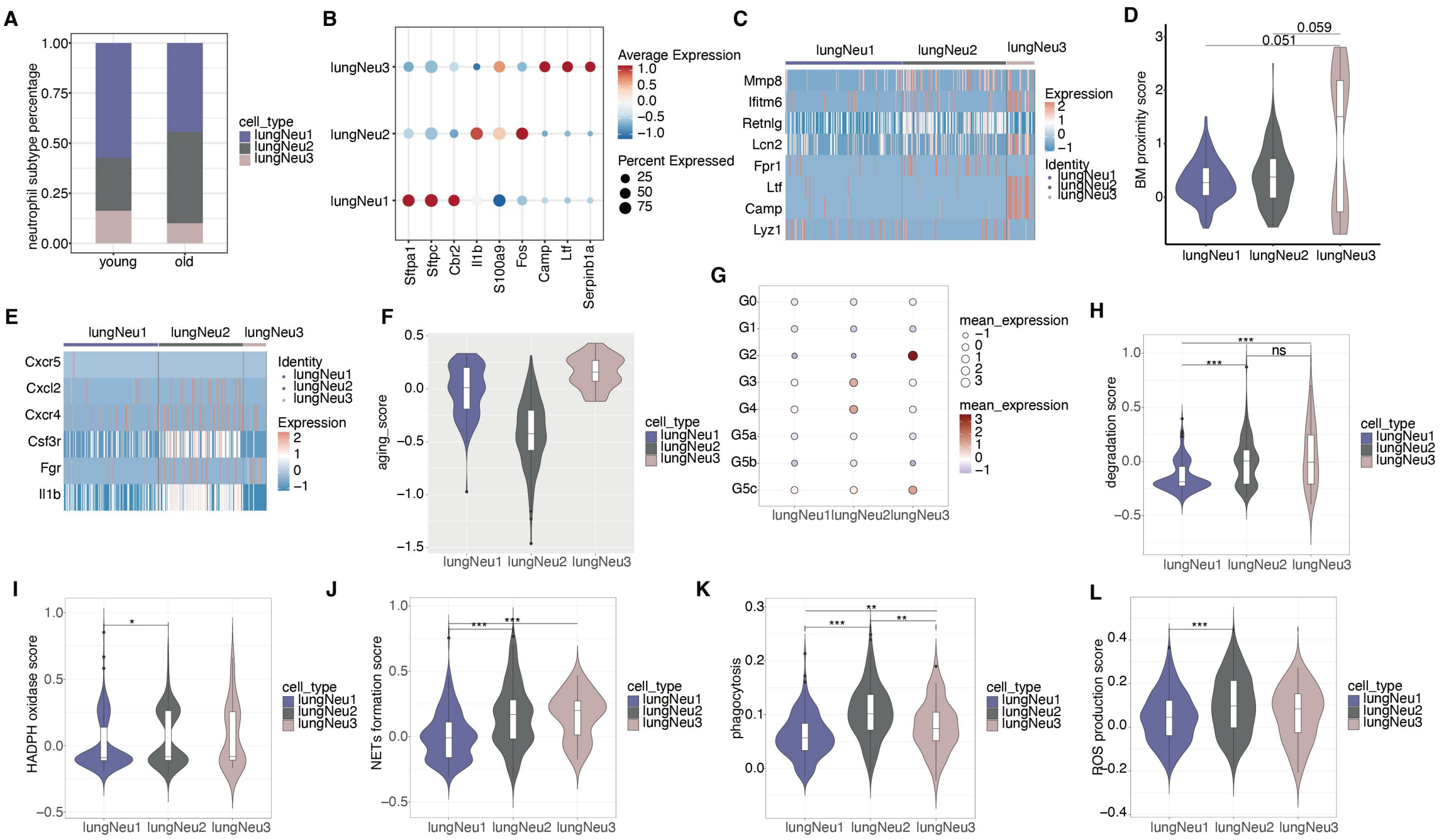

Supplementary Figure 7. Functional characterization of aging-related neutrophils in lung tissues. A, Bar plot showing the relative proportions of neutrophil subsets. Bar height indicates the percentage of neutrophil subsets, and colors denote different neutrophil subsets. **B**, Dot plot showing the top three marker genes for each neutrophil subset. Dot size indicates the percentage of cells expressing the gene, and color reflects average expression levels. **C**, Heatmap displaying the top 3 marker genes for each neutrophil subset. Colors in the bar indicate subsets, while heatmap colors represent normalized gene expression levels. **D**, violin plot showing the corresponding BM similarity scores across subsets. Embedded boxplots display median scores, with whiskers denoting non-outlier ranges. **E**, Heatmap showing the expression of aging-related clock genes across cells. Color scale represents expression intensity. **F**, Violin plot (right) showing aging scores across neutrophil subsets. Embedded boxplots indicate median values with whiskers denoting non-outlier ranges. **G**, Dot plot showing the similarity scores of Xie's neutrophil signatures across different subsets. Both color and dot size represent mean similarity scores. **H**, Violin plot showing degranulation scores across neutrophil subsets, with embedded boxplots showing median values and whiskers denoting non-outlier ranges. **I**, Violin plots showing NADPH oxidase activity scores across neutrophil subsets. Embedded boxplots indicate medians with whiskers denoting non-outlier ranges. **J**, Violin plot showing neutrophil extracellular trap (NET) formation scores across subsets. Embedded boxplots indicate medians with whiskers denoting non-outlier ranges. **K**, Violin plot showing phagocytosis scores across neutrophil subsets, with embedded boxplots showing median values and whiskers denoting non-outlier ranges. **L**, Violin plots showing reactive oxygen species (ROS) generation scores across neutrophil subsets. Embedded boxplots indicate medians with whiskers denoting non-outlier ranges.

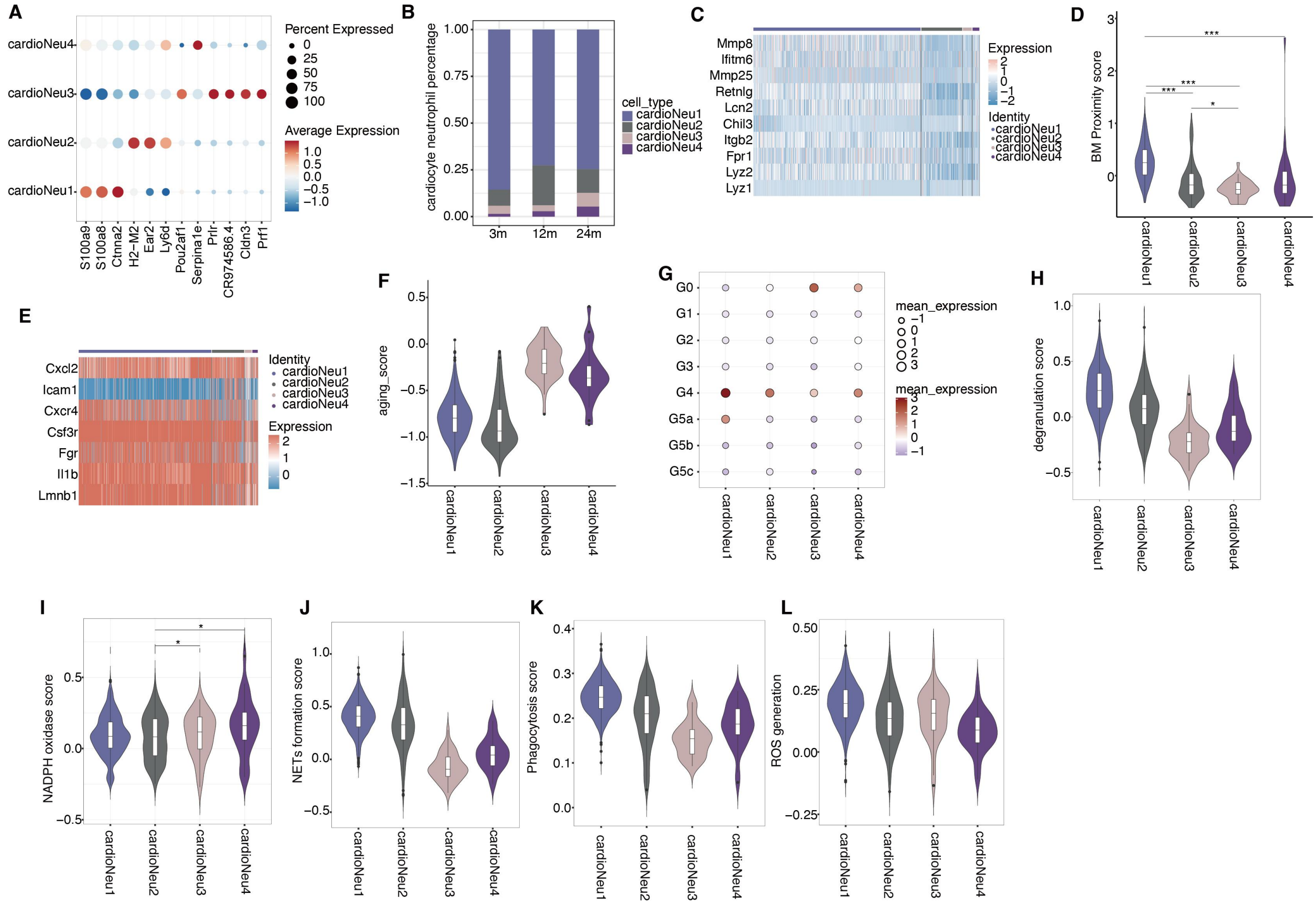

Supplementary Figure 8. Functional characterization of aging-related neutrophils in heart tissue. A, Dot plot showing the top three marker genes for each neutrophil subset. Dot size indicates the percentage of cells expressing the gene, and color reflects average expression levels. **B,** Bar plot showing the relative proportions of neutrophil subsets. Bar height indicates the percentage of neutrophil subsets, and colors denote different neutrophil subsets. **C,** Heatmap displaying the top 3 marker genes for each neutrophil subset. Colors in the bar indicate subsets, while heatmap colors represent normalized gene expression levels. **D,** violin plot showing the corresponding BM similarity scores across subsets. Embedded boxplots display median scores, with whiskers denoting non-outlier ranges. **E,** Heatmap showing the expression of aging-related clock genes across cells. Color scale represents expression intensity. **F,** Violin plot (right) showing aging scores across neutrophil subsets. Embedded boxplots indicate median values with whiskers denoting non-outlier ranges. **G,** Dot plot showing the similarity scores of Xie's neutrophil signatures across different subsets. Both color and dot size represent mean similarity scores. **H,** Violin plot showing degranulation scores across neutrophil subsets, with embedded boxplots showing median values and whiskers denoting non-outlier ranges. **I,** Violin plots showing NADPH oxidase activity scores across neutrophil subsets. Embedded boxplots indicate medians with whiskers denoting non-outlier ranges. **J,** Violin plot showing neutrophil extracellular trap (NET) formation scores across subsets. Embedded boxplots indicate medians with whiskers denoting non-outlier ranges. **K,** Violin plot showing phagocytosis scores across neutrophil subsets, with embedded boxplots showing median values and whiskers denoting non-outlier ranges. **L,** Violin plots showing reactive oxygen species (ROS) generation scores across neutrophil subsets. Embedded boxplots indicate medians with whiskers denoting non-outlier ranges.

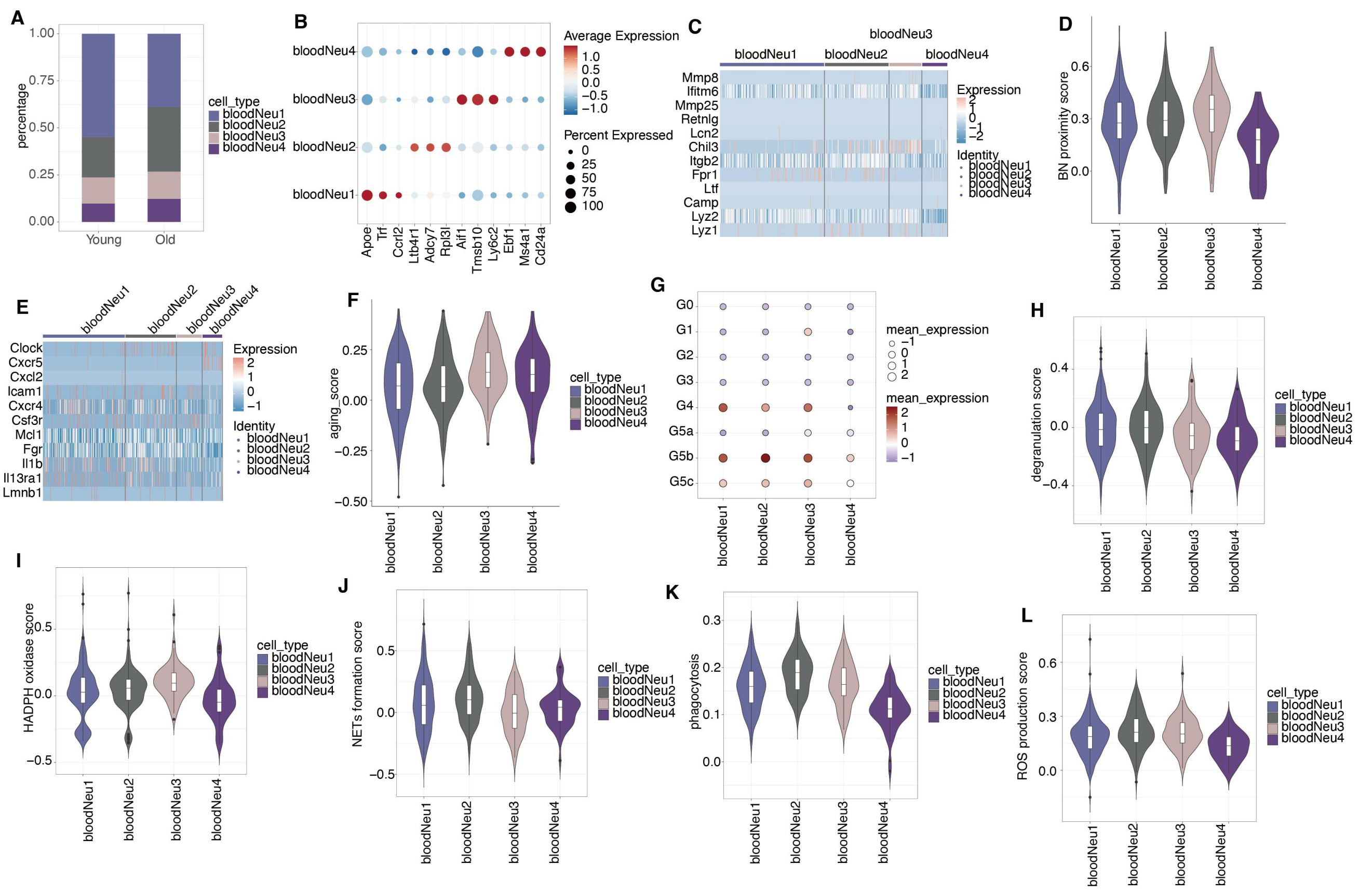

Supplementary Figure 9. Functional characterization of aging-related neutrophils in blood. A, Bar plot showing the relative proportions of neutrophil subsets. Bar height indicates the percentage of neutrophil subsets, and colors denote different neutrophil subsets. **B**, Dot plot showing the top three marker genes for each neutrophil subset. Dot size indicates the percentage of cells expressing the gene, and color reflects average expression levels. **C**, Heatmap displaying the top 3 marker genes for each neutrophil subset. Colors in the bar indicate subsets, while heatmap colors represent normalized gene expression levels. **D**, violin plot showing the corresponding BM similarity scores across subsets. Embedded boxplots display median scores, with whiskers denoting non-outlier ranges. **E**, Heatmap showing the expression of aging-related clock genes across cells. Color scale represents expression intensity. **F,** Violin plot (right) showing aging scores across neutrophil subsets. Embedded boxplots indicate median values with whiskers denoting non-outlier ranges. **G,** Dot plot showing the similarity scores of Xie's neutrophil signatures across different subsets. Both color and dot size represent mean similarity scores. **H,** Violin plot showing degranulation scores across neutrophil subsets, with embedded boxplots showing median values and whiskers denoting non-outlier ranges. **I,** Violin plots showing NADPH oxidase activity scores across neutrophil subsets. Embedded boxplots indicate medians with whiskers denoting non-outlier ranges. **J,** Violin plot showing neutrophil extracellular trap (NET) formation scores across subsets. Embedded boxplots indicate medians with whiskers denoting non-outlier ranges. **K,** Violin plot showing phagocytosis scores across neutrophil subsets, with embedded boxplots showing median values and whiskers denoting non-outlier ranges. **L,** Violin plots showing reactive oxygen species (ROS) generation scores across neutrophil subsets. Embedded boxplots indicate medians with whiskers denoting non-outlier ranges.

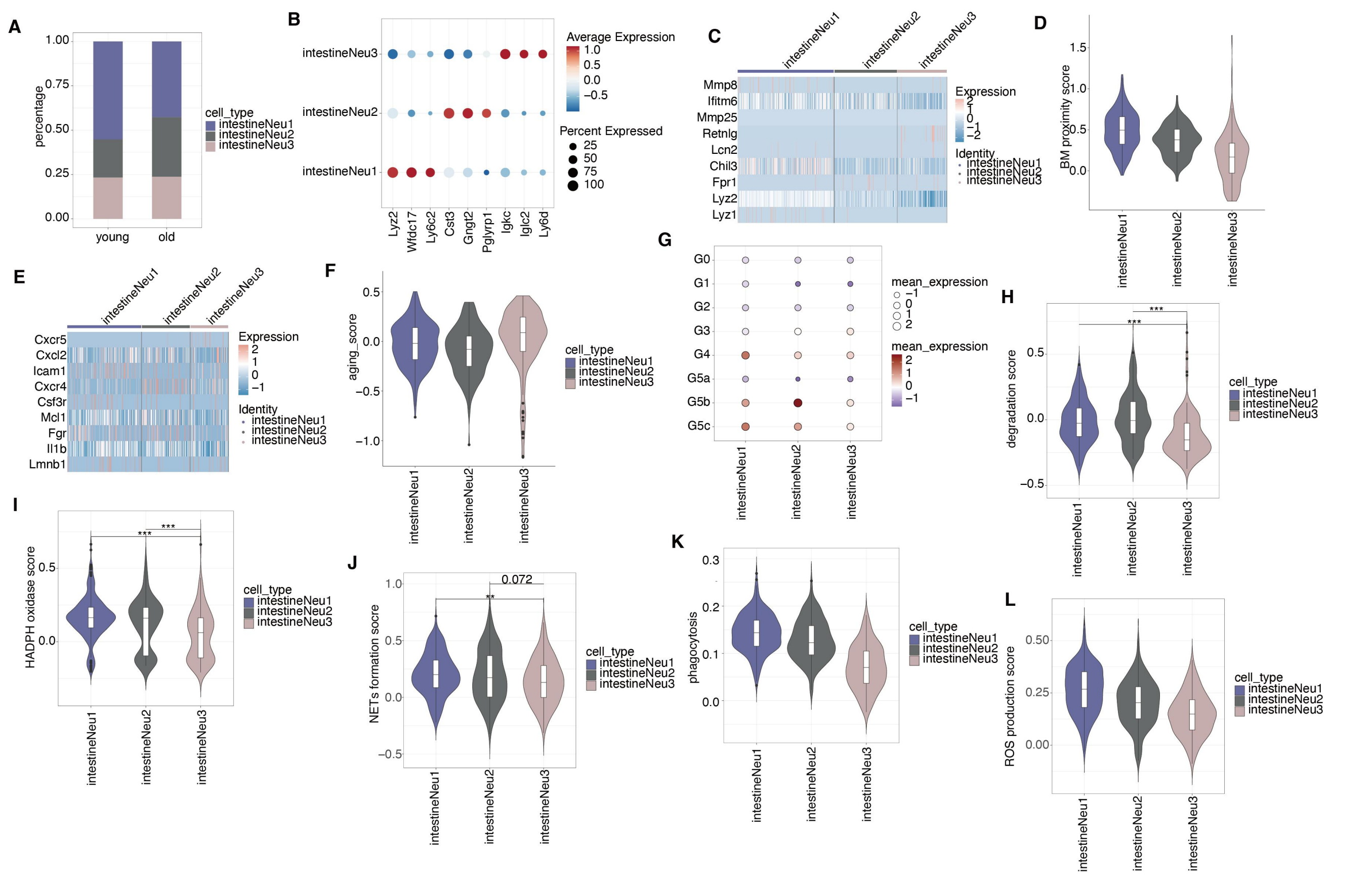

Supplementary Figure 10. Functional characterization of aging-related neutrophils in intestine. A, Bar plot showing the relative proportions of neutrophil subsets. Bar height indicates the percentage of neutrophil subsets, and colors denote different neutrophil subsets. **B,** Dot plot showing the top three marker genes for each neutrophil subset. Dot size indicates the percentage of cells expressing the gene, and color reflects average expression levels. **C,** Heatmap displaying the top 3 marker genes for each neutrophil subset. Colors in the bar indicate subsets, while heatmap colors represent normalized gene expression levels. **D,** violin plot showing the corresponding BM similarity scores across subsets. Embedded boxplots display median scores, with whiskers denoting non-outlier ranges. **E,** Heatmap showing the expression of aging-related clock genes across cells. Color scale represents expression intensity. **F,** Violin plot (right) showing aging scores across neutrophil subsets. Embedded boxplots indicate median values with whiskers denoting non-outlier ranges. **G,** Dot plot showing the similarity scores of Xie's neutrophil signatures across different subsets. Both color and dot size represent mean similarity scores. **H,** Violin plot showing degranulation scores across neutrophil subsets, with embedded boxplots showing median values and whiskers denoting non-outlier ranges. **I,** Violin plots showing NADPH oxidase activity scores across neutrophil subsets. Embedded boxplots indicate medians with whiskers denoting non-outlier ranges. **J,** Violin plot showing neutrophil extracellular trap (NET) formation scores across subsets. Embedded boxplots indicate medians with whiskers denoting non-outlier ranges. **K,** Violin plot showing phagocytosis scores across neutrophil subsets, with embedded boxplots showing median values and whiskers denoting non-outlier ranges. **L,** Violin plots showing reactive oxygen species (ROS) generation scores across neutrophil subsets. Embedded boxplots indicate medians with whiskers denoting non-outlier ranges.

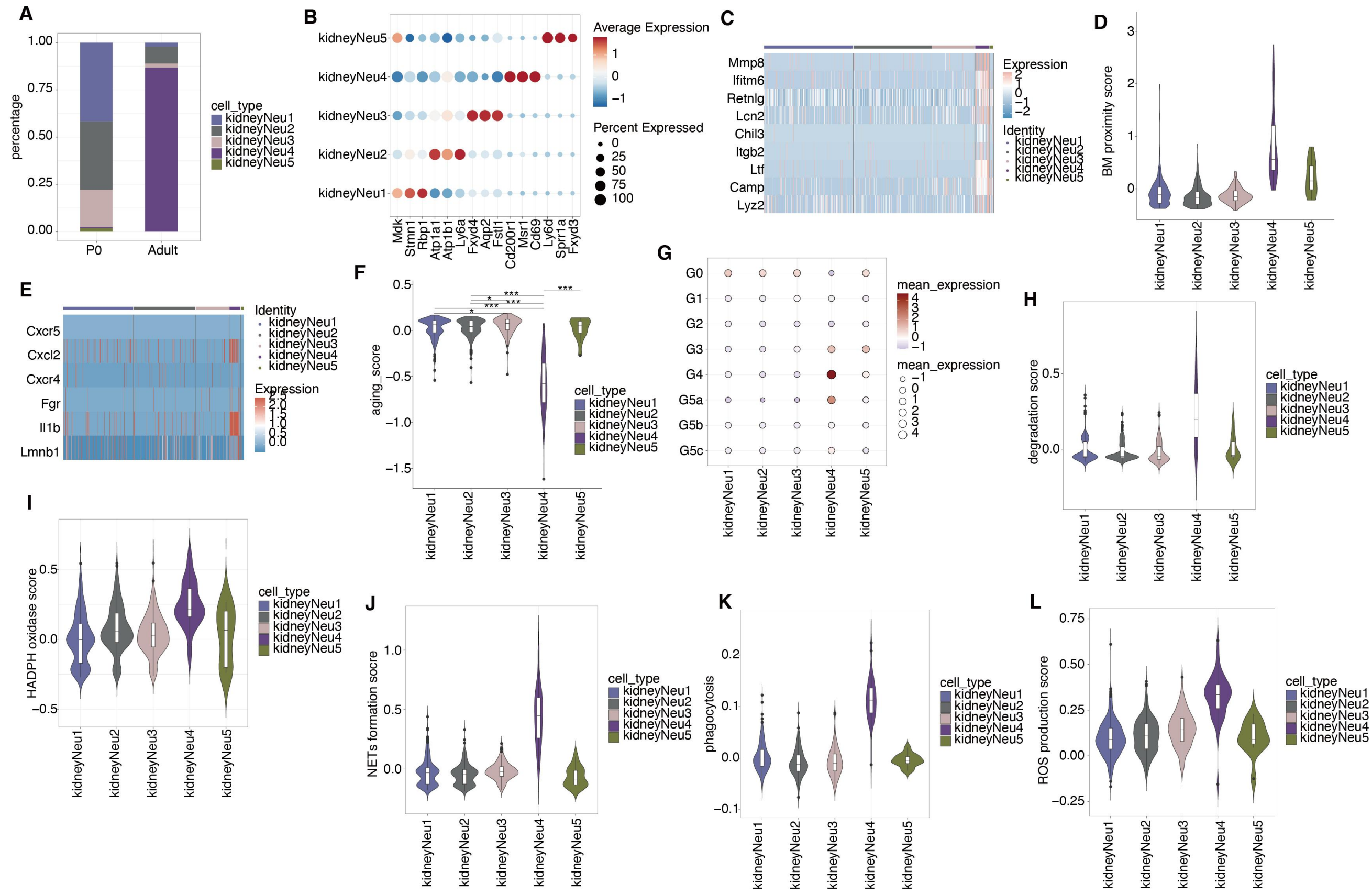

Supplementary Figure 11. Functional characterization of aging-related neutrophils in kidney. A, Bar plot showing the relative proportions of neutrophil subsets. Bar height indicates the percentage of neutrophil subsets, and colors denote different neutrophil subsets. **B,** Dot plot showing the top three marker genes for each neutrophil subset. Dot size indicates the percentage of cells expressing the gene, and color reflects average expression levels. **C,** Heatmap displaying the top 3 marker genes for each neutrophil subset. Colors in the bar indicate subsets, while heatmap colors represent normalized gene expression levels. **D,** violin plot showing the corresponding BM similarity scores across subsets. Embedded boxplots display median scores, with whiskers denoting non-outlier ranges. **E,** Heatmap showing the expression of aging-related clock genes across cells. Color scale represents expression intensity. **F,** Violin plot (right) showing aging scores across neutrophil subsets. Embedded boxplots indicate median values with whiskers denoting non-outlier ranges. **G,** Dot plot showing the similarity scores of Xie's neutrophil signatures across different subsets. Both color and dot size represent mean similarity scores. **H,** Violin plot showing degranulation scores across neutrophil subsets, with embedded boxplots showing median values and whiskers denoting non-outlier ranges. **I,** Violin plots showing NADPH oxidase activity scores across neutrophil subsets. Embedded boxplots indicate medians with whiskers denoting non-outlier ranges. **J,** Violin plot showing neutrophil extracellular trap (NET) formation scores across subsets. Embedded boxplots indicate medians with whiskers denoting non-outlier ranges. **K,** Violin plot showing phagocytosis scores across neutrophil subsets, with embedded boxplots showing median values and whiskers denoting non-outlier ranges. **L,** Violin plots showing reactive oxygen species (ROS) generation scores across neutrophil subsets. Embedded boxplots indicate medians with whiskers denoting non-outlier ranges.

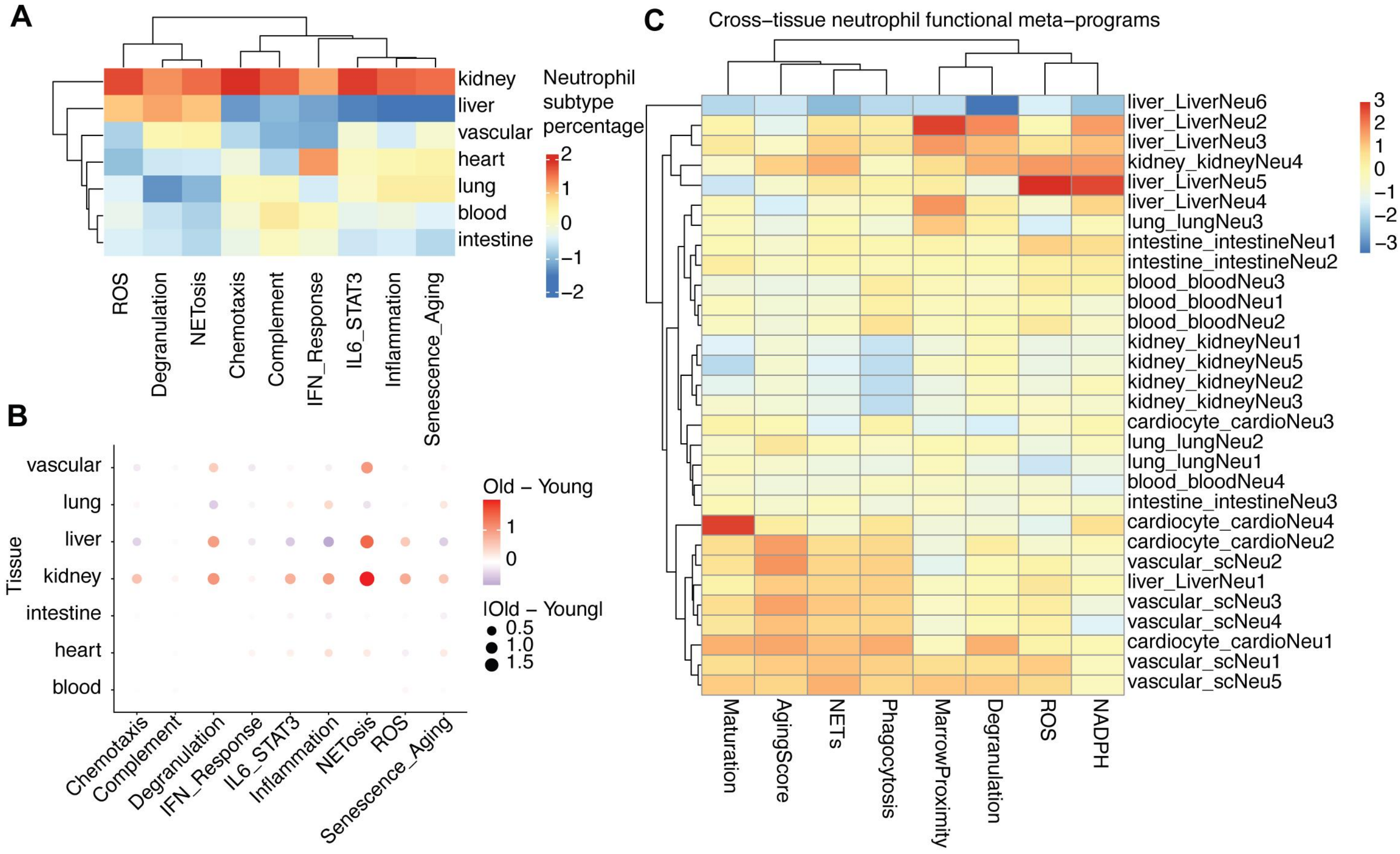

Supplementary Figure 12. Tissue-specific enrichment of aging-associated neutrophil programs and neutrophil states across organs. A.Heatmap showing the relative enrichment of neutrophil functional signatures across tissues. Functional programs, including reactive oxygen species (ROS) production, degranulation, NETosis, complement activation, interferon (IFN) response, IL-6/STAT3 signaling, inflammation, and cellular aging/senescence, were scored in neutrophils from each tissue and averaged at the tissue level. Colors represent scaled signature scores (Z-scores), with red indicating higher enrichment and blue indicating lower enrichment. Hierarchical clustering was performed on both tissues and functional signatures. **B.** Dot plot illustrating age-associated changes in neutrophil functional programs across tissues. Dot color represents the difference in signature scores between aged and young mice (Old − Young), whereas dot size indicates the absolute magnitude of the change. Positive values (red) denote increased pathway activity in aged neutrophils, and negative values (purple) indicate reduced activity. Notably, NETosis, IL-6/STAT3 signaling, inflammatory responses, and ROS-related programs were preferentially increased in aged neutrophils across multiple tissues. **C.** Identification of cross-tissue neutrophil functional meta-programs. Neutrophil subclusters from individual tissues were grouped according to their functional signature profiles, including maturation, aging score, NETosis, phagocytosis, bone marrow proximity, degranulation, ROS production, and NADPH oxidase activity. Each row represents a tissue-specific neutrophil subtype, and columns represent functional programs. Values are shown as scaled signature scores (Z-scores). Hierarchical clustering revealed conserved functional meta-programs shared across tissues, highlighting common neutrophil activation states characterized by inflammatory, oxidative, and maturation-associated signatures.

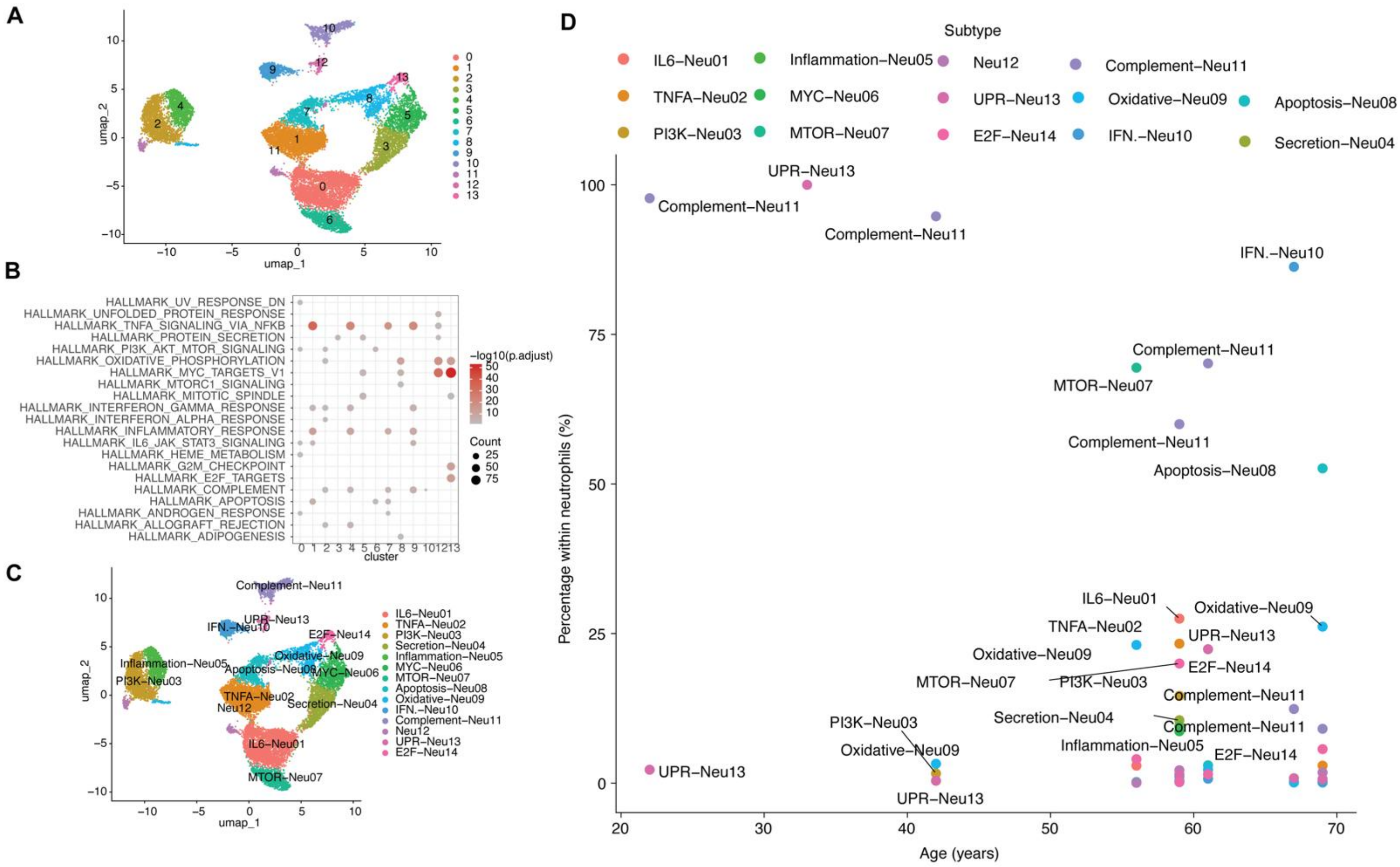

Supplementary Figure 13. Human single-cell atlas analysis validates conserved aging-associated neutrophil states across tissues. A. UMAP visualization of integrated human neutrophils following Harmony-based batch correction and graph-based clustering. 14 transcriptionally distinct neutrophil states were identified and numbered according to cluster identity. **B.** Hallmark pathway enrichment analysis of cluster-specific marker genes. Dot size represents the number of enriched genes within each pathway, and color intensity indicates enrichment significance (−log10 adjusted P value). Distinct neutrophil states were characterized by pathways involved in inflammatory signaling, oxidative stress, complement activation, the unfolded protein response, cell-cycle regulation, interferon signaling, and metabolic remodeling. **C.** Functional annotation of human neutrophil states based on dominant enriched pathways. Identified states included IL6-associated neutrophils (IL6–Neu01), TNFA-associated neutrophils (TNFA–Neu02), PI3K-signaling neutrophils (PI3K–Neu03), secretory neutrophils (Secretion–Neu04), inflammatory neutrophils (Inflammation–Neu05), MYC-associated neutrophils (MYC–Neu06), MTOR-associated neutrophils (MTOR–Neu07), apoptosis-associated neutrophils (Apoptosis–Neu08), oxidative stress-associated neutrophils (Oxidative–Neu09), interferon-responsive neutrophils (IFN–Neu10), complement-associated neutrophils (Complement–Neu11), Neu12, unfolded protein response-associated neutrophils (UPR–Neu13), and E2F-associated neutrophils (E2F–Neu14). **D.** Age-dependent distribution of human neutrophil states. Each point represents the percentage of a given neutrophil state within an individual donor. Several aging-associated neutrophil populations, including Complement–Neu11, Oxidative–Neu09, IL6–Neu01, and UPR–Neu13, showed increased abundance with advancing age, whereas other states remained relatively stable.

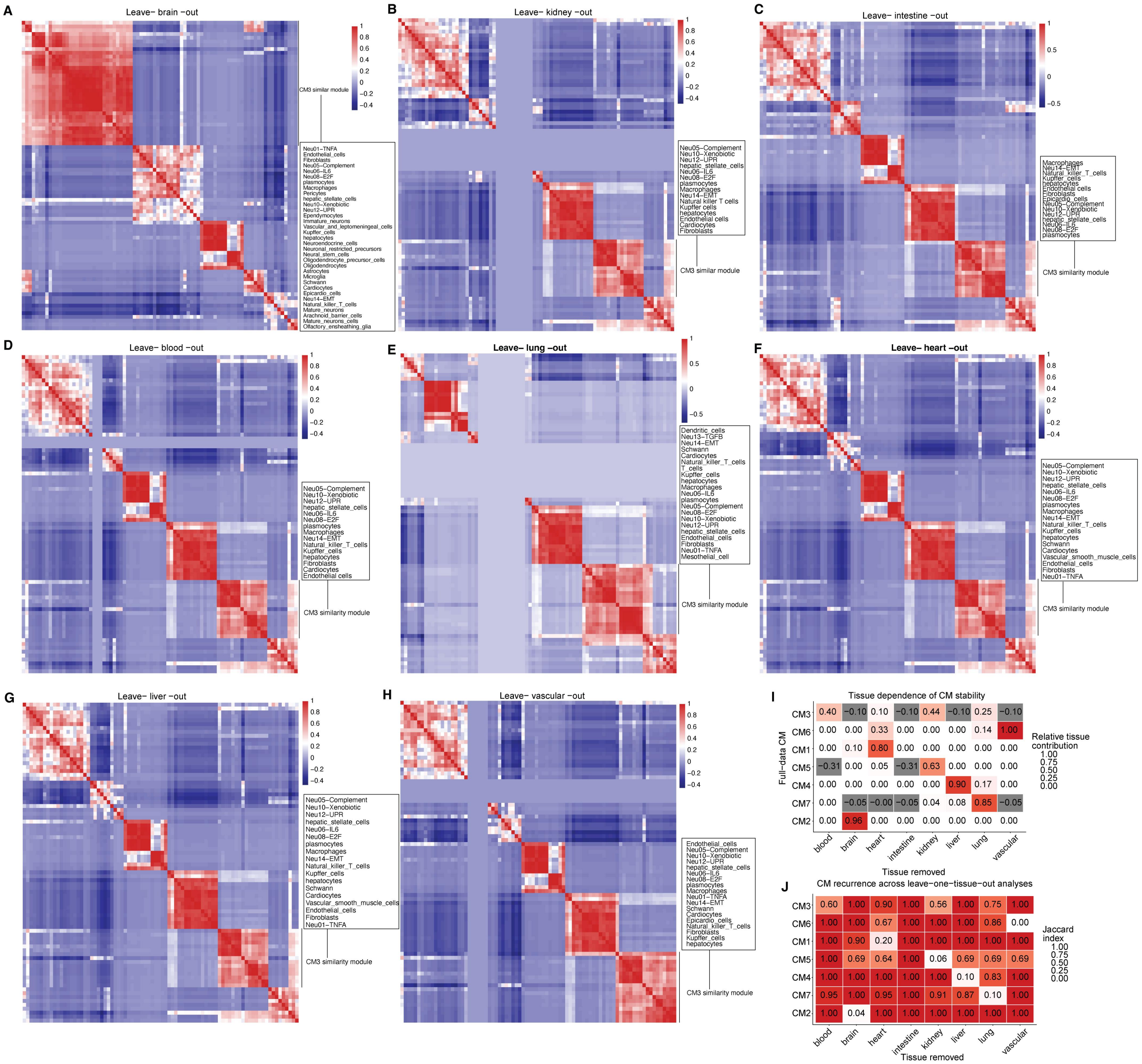

Supplementary Figure 14. Leave-one-tissue-out analysis demonstrates the robustness of neutrophil transcriptional programs across organs. A-H, Pairwise correlation matrices of aging-associated neutrophil transcriptional programs generated using a leave-one-tissue-out strategy. For each analysis, neutrophils from one tissue were excluded before recalculating gene–gene correlation networks: brain (**A**), kidney (**B**), intestine (**C**), blood (**D**), lung (**E**), heart (**F**), liver (**G**), and vascular tissue (**H**). Genes are ordered by hierarchical clustering based on the correlation structure. Red indicates positive correlations, whereas blue indicates negative correlations. **I,** Tissue-dependency scores for each full-data co-abundance module (CM) following sequential removal of individual tissues. The tissue-dependency score represents the reduction in CM recurrence associated with removal of the indicated tissue relative to the other LOTO iterations; larger positive values indicate greater dependence of a CM on that tissue. **J,** Recurrence of full-data CMs across LOTO iterations. For each iteration, one tissue was excluded, CMs were independently reconstructed using the same clustering procedure, and each full-data CM was matched to the LOTO-derived module with the greatest membership overlap. Values indicate the Jaccard similarity between the full-data CM and its best-matched LOTO module.

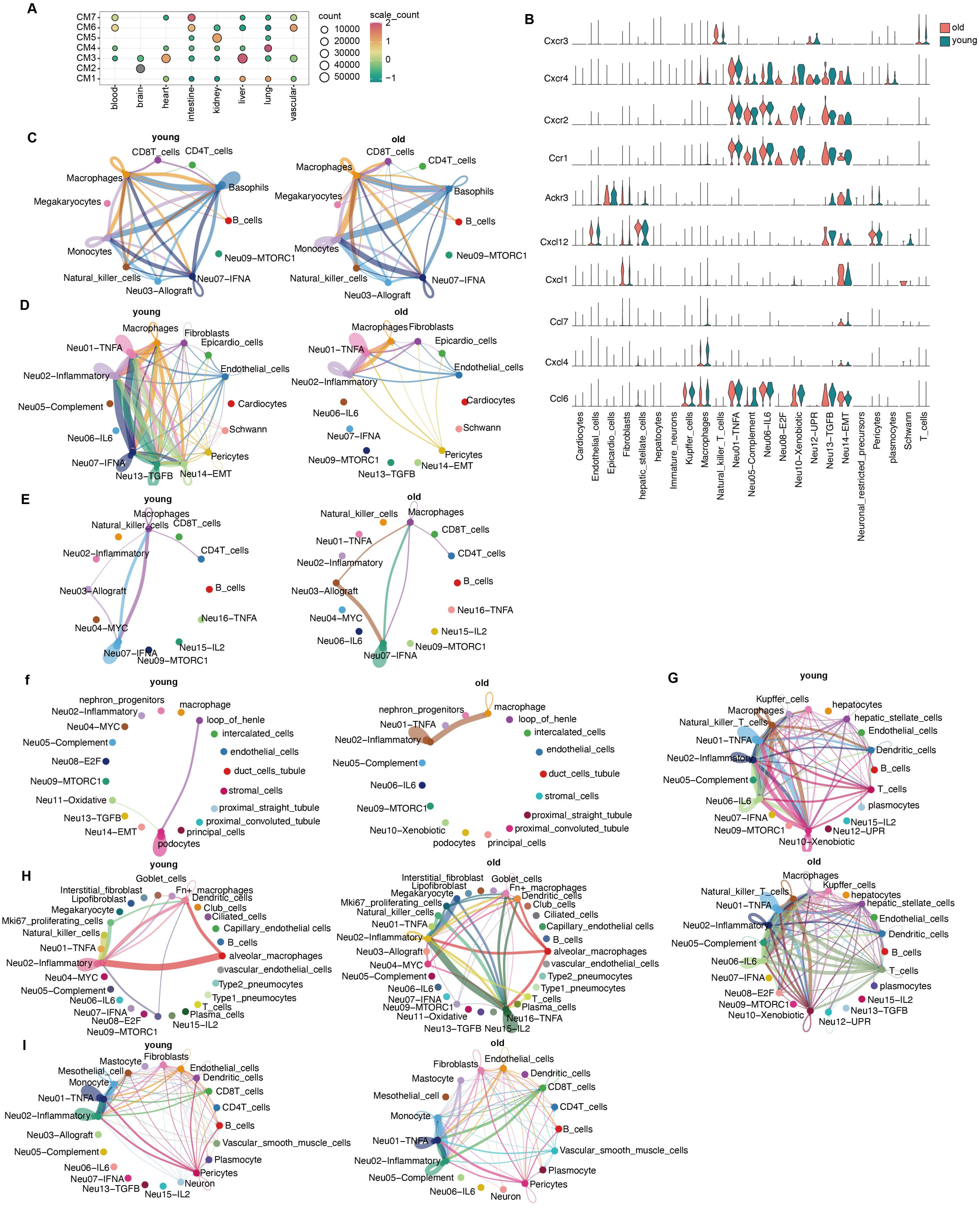

Supplementary Figure 15. The cell-cell communication networks across different neutrophils. A, The dot plot showing different cell component expression across different tissues. The size of the dot represent cell count in tissues. The color of the dot represents the gene expression in each tissue. **B,** The violin plot of the chemokine expression in different cell populations and neutrophil subsets. Color representing the old and young groups. **C – I,** The chordal plot of different cell populations across different groups in blood (**C**), heart (**D**), intestine (**E**), kidney (**F**), liver (**G**), lung (**H**), and vascular (**I**) tissues. Color representing the

different cell types. The width of the edges shows the strength of the communication among cells.

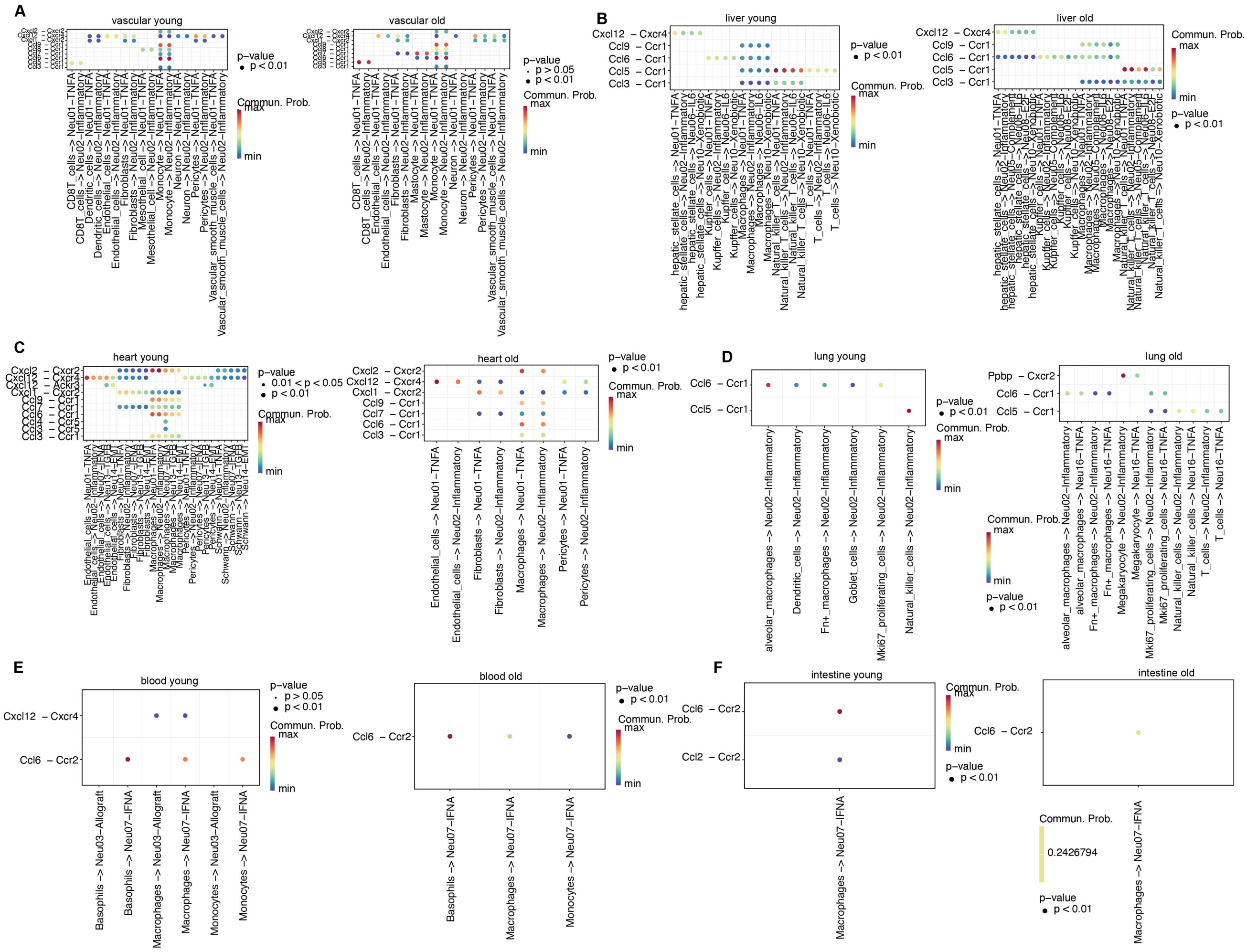

Supplementary Figure 16. The communication of chemokines between different cell types in tissues. A-**F,** the dot plot showing cell-cell communication strength of chemokines in vascular (**A**), liver (**B**), heart (**C**), lung (**D**), blood (**E**), and intestine (**F**). The color represents chemokine strength between two cell types.

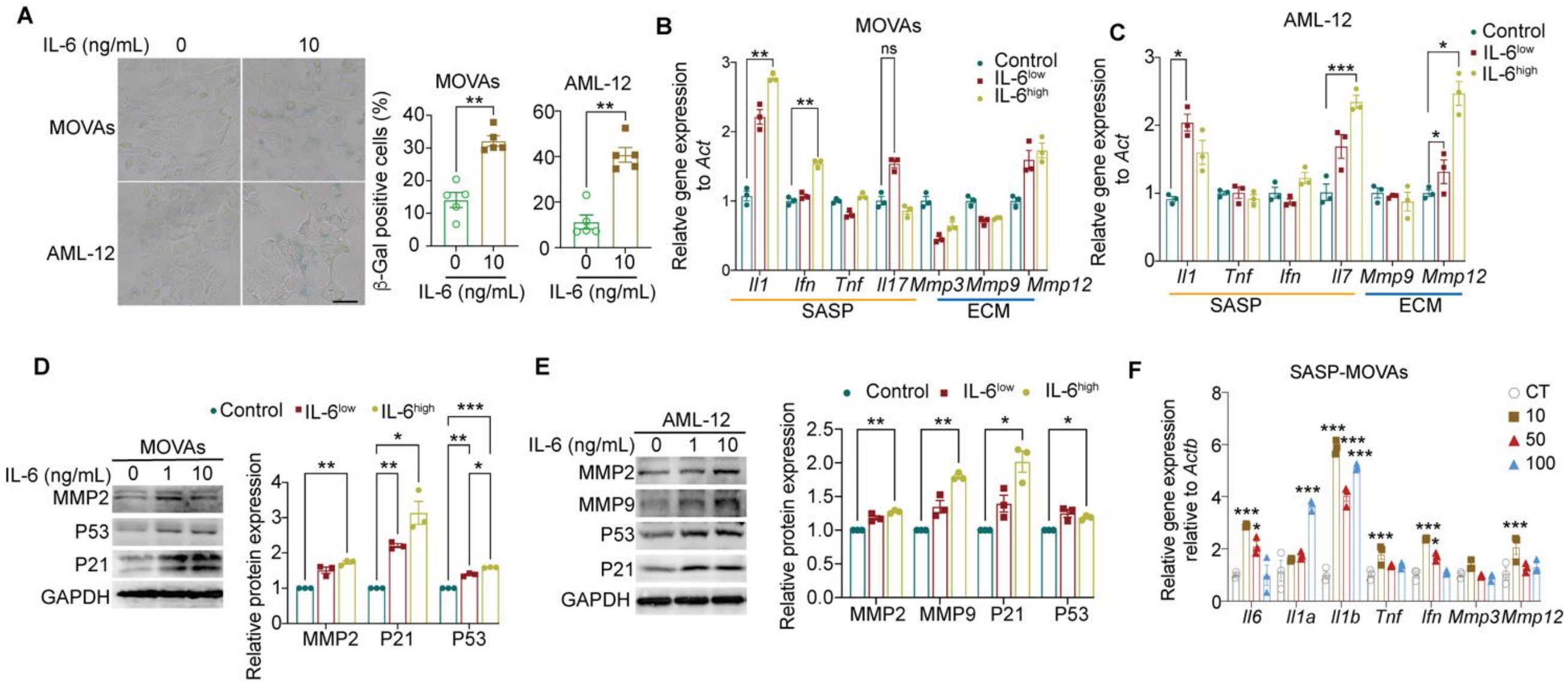

Supplementary Figure 17. IL-6 stimulation induces MOVAS and AML-12 cell senescence-associated response. A. b-Gal staining of MOVAS and AML-12 cells after treatment with IL-6 at indicated dose. bar=20 mm. **B-C.** RNA levels of SASP and ECM-related genes (*Il1, Tnf, Ifn, Il17, Mmp3, Mmp9 and Mmp12*) in MOVAS following treatment with IL-6. IL-6^{high}=10ng/mL, IL-6^{low}=1ng/mL. **D-E.** Protein expression of indicated markers in MOVAS and AML-12 cells after treatment with IL-6 at the indicated dose. **F.** RNA levels of SASP and ECM-related genes (*Il6, Il1a, Il1b, Tnf, Ifn, Mmp3, Mmp12*) in MOVAS following treatment with conditioned medium from HL-60 after stimulation with IL-6 at the indicated dose. Data are shown as the mean ± s.e.m., *P < 0.05; **P < 0.01; ***P < 0.001. t-test was used in **A**. *Two-way ANOVA* followed by Tukey's multiple comparisons test was used in **B-F**.

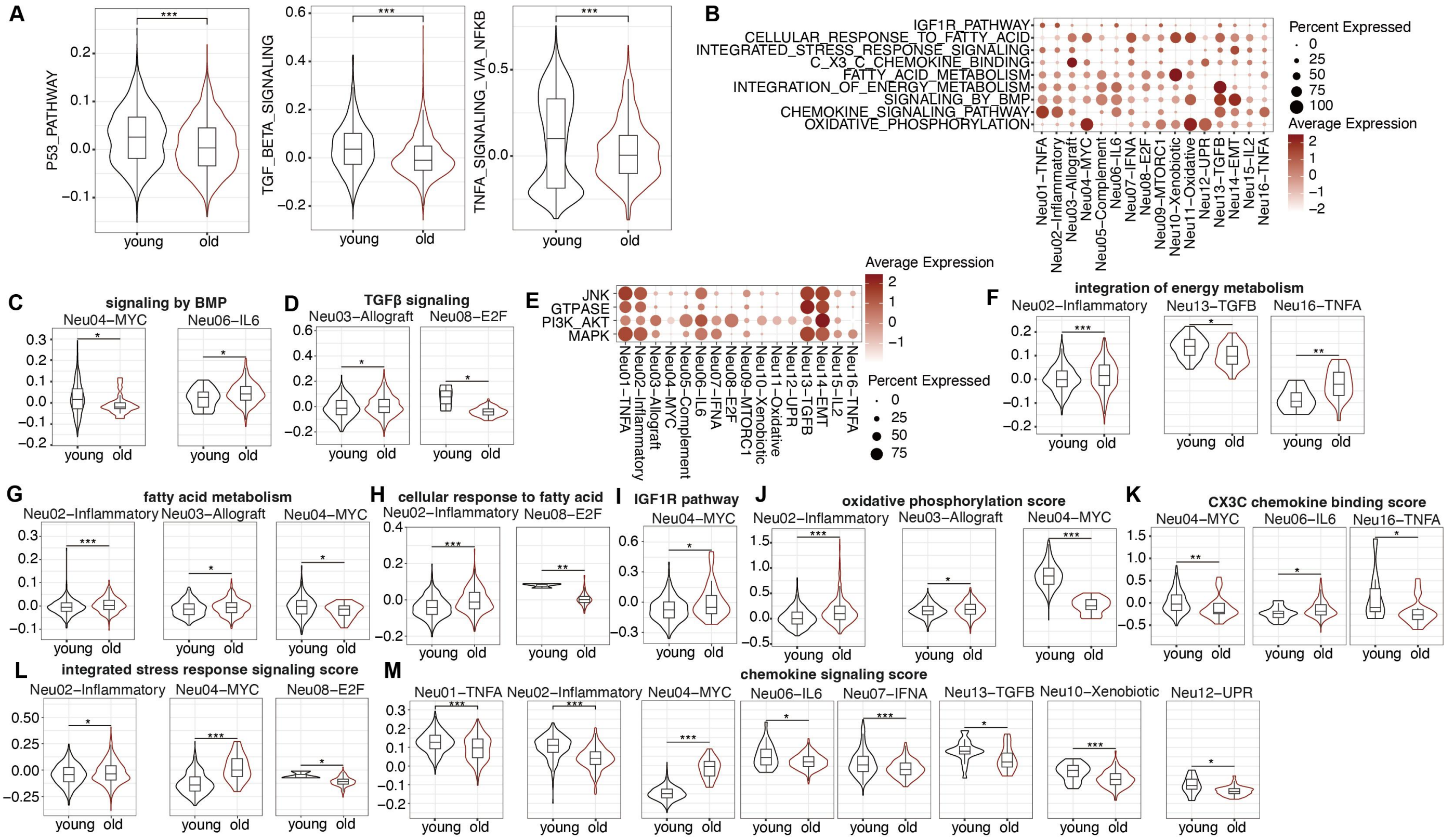

Supplementary Figure 18. The important aging-related pathway alterations in neutrophil subtypes. A, The violin plot showing p53 (left), TGFβ (middle), and TNFα (right) alterations in Neu06-IL6 from young and aged neutrophils, with embedded boxplots showing median values and whiskers denoting non-outlier ranges. **B,** The dot plot showing important pathway alterations in different neutrophil subtypes. The size indicates the percentage of pathway-positive cells. The color represents the pathway average expression of neutrophil subtypes. **C,** The violin plot showing BMP signaling expression in Neu04-MYC and Neu06-IL6, with embedded boxplots showing median values and whiskers denoting non-outlier ranges. **D,** The violin plot showing TGFβ signaling expression in Neu03-Allograft and Neu08-E2F, with embedded boxplots showing median values and whiskers denoting non-outlier ranges. **E,** The dot plot showing different inflammation pathway expression in different neutrophil subtypes. The size represents the expression of the inflammatory pathway, and the color shows the average expression across different neutrophil subtypes. **F,** The violin plot showing integration of energy metabolism in Neu02-inflammatory and Neu13-TGFB, and Neu16-TNFA, with embedded boxplots showing median values and whiskers denoting non-outlier ranges. **G,** The violin plot showing fatty acid metabolism in Neu02-inflammatory and Neu3-Allograft, and Neu04-MYC, with embedded boxplots showing median values and whiskers denoting non-outlier ranges. **H,** The violin plot showing cellular response to fatty acid in Neu02-inflammatory and Neu08-E2F, with embedded boxplots showing median values and whiskers denoting non-outlier ranges. **I,** The violin plot showing IGF1R pathway in Neu04-MYC, with embedded boxplots showing median values and whiskers denoting non-outlier ranges. **J,** The violin plot showing oxidative phosphorylation score in Neu02-inflammatory, Neu3-Allograft and Neu04-MYC, with embedded boxplots showing median values and whiskers denoting non-outlier ranges. **K,** The violin plot showing CX3C chemokine binding score in Neu04-MYC, Neu06-IL6 and Neu16-TNFA, with embedded boxplots showing median values and whiskers denoting non-outlier ranges. **L,** The violin plot showing integrated stress response signaling score in Neu02-inflammatory, Neu04-MYC,

and Neu08-E2F, with embedded boxplots showing median values and whiskers denoting non-outlier ranges. **M,** The violin plot showing chemokine signaling score in Neu01-TNFA, Neu02-inflammatory, Neu04-MYC, Neu06-IL6, Neu07-IFNA, Neu13-TGFB, Neu10-Xenobiotic and Neu12-UPR, with embedded boxplots showing median values and whiskers denoting non-outlier ranges.

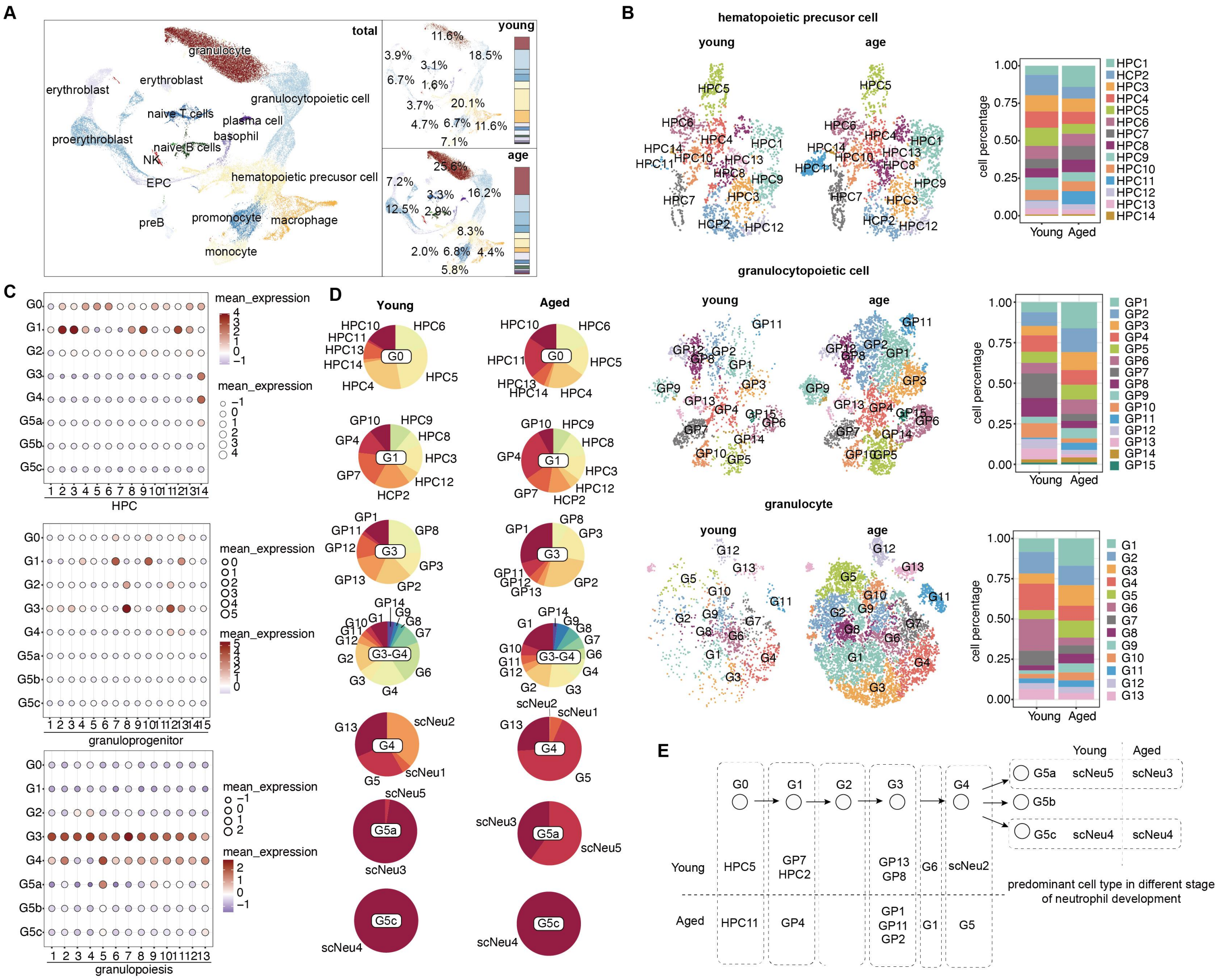

Supplementary Figure 19. Bone marrow single-cell landscape of young and aged mice. A, UMAP plot showing cell population distribution across different groups. Bar plot showing the cell populations in young and aged mice. Color represents the cell populations in a mouse's bone marrow. **B,** UMAP plot showing hematopoietic precursor cells, granulocytopoiesis cells, and granulocytes in bone marrow of a mouse (left panel). Bar plot showing the relative proportions of cell populations. Bar height indicates the percentage of cells, and colors denote different cell subsets (right panel). **C,** Dot plot showing the similarity scores of Xie's neutrophil signatures across different subsets in HPC, granulocyte progenitor, and granulocyte. Both color and dot size represent mean similarity scores. **D,** The pie chart showing the cell percentages across different neutrophil stages in young and aged mice, with colors representing the different bone marrow cell types. **E,** The landscape of neutrophil development in bone marrow.
